# M133S mutation possibly involve in the ER stress and mitophagy pathway in maintenance hemodialysis patients with occult hepatitis B infection

**DOI:** 10.1038/s41598-024-64943-3

**Published:** 2024-06-17

**Authors:** Yurong Zou, Sipei Chen, Yiyuan Cui, Yang Zou

**Affiliations:** 1 Department of Nephrology and Institute of Nephrology, Sichuan Provincial People’s Hospital, School of Medicine, University of Electronic Science and Technology of China, Sichuan Clinical Research Centre for Kidney Diseases, Chengdu, 610072 Sichuan China; 2 Laboratory of Neurodegenerative Disorders, Department of Neurology, Rare Disease Center, West China Hospital, Sichuan University, Chengdu, 610041 Sichuan China

**Keywords:** Occult hepatitis B virus infection (OBI), Maintenance hemodialysis (MHD), Gene mutations, Endoplasmic reticulum (ER), Mitophagy, Diseases, Pathogenesis

## Abstract

Occult hepatitis B virus infection (OBI) is characterized by the presence of HBV DNA in the absence of detectable HBsAg. OBI is an important risk factor for cirrhosis and hepatocellular carcinoma, but its pathogenesis has not been fully elucidated. Mutations in the HBV preS/S genes can lead to impaired secretion of either HBsAg or S-protein resulting in the accumulation of defective viruses or S protein in cells. In our previous work, the M133S mutation was present in the HBV S gene of maintenance hemodialysis (MHD) patients with OBI. In this study, we investigated the potential role of amino acid substitutions in S proteins in S protein production and secretion through the construction of mutant S gene plasmids, structural prediction, transcriptome sequencing analysis, and in vitro functional studies. Protein structure prediction showed that the S protein M133S mutant exhibited hydrophilic modifications, with greater aggregation and accumulation of the entire structure within the membrane phospholipid bilayer. Differential gene enrichment analysis of transcriptome sequencing data showed that differentially expressed genes were mainly concentrated in protein processing in the endoplasmic reticulum (ER). The expression of heat shock family proteins and ER chaperone molecules was significantly increased in the wild-type and mutant groups, whereas the expression of mitochondria-associated proteins was decreased. Immunofluorescence staining and protein blotting showed that the endoplasmic reticulum-associated protein PDI, the autophagy marker LC3, and the lysosome-associated protein LAMP2 co-localized with the S proteins in the wild-type and mutant strains, and their expression was increased. The mitochondria-associated TOMM20 protein was also co-expressed with the S protein, but expression was significantly reduced in the mutant. The M133S mutation in the S gene is expressed as a defective and misfolded protein that accumulates in the endoplasmic reticulum causing secretion-impaired endoplasmic reticulum stress, which in turn triggers mitochondrial autophagy and recruits lysosomes to fuse with the autophagosome, leading to mitochondrial clearance. This study preliminarily demonstrated that the mutation of M133S in the S gene can cause OBI and is associated with disease progression, providing a theoretical basis for the diagnosis and treatment of OBI.

## Introduction

Hepatitis B virus (HBV) infection is a major global health concern, affecting over 296 million people worldwide^1^. Despite efforts to detect and treat the virus, there is still a significant portion of people who are unaware of their infection^2^. Screening tests, such as those for hepatitis B surface antigens (HBsAg) and anti-hepatitis B core (HBc) antibodies, have reduced the spread of HBV infection, but fail to detect occult HBV infection (OBI)^3^. OBI is defined as the presence of replication-competent HBV DNA in the liver or blood of individuals who have tested negative for HBsAg using laboratory-based immunoassays or rapid diagnostic tests^4^.This form of clinical entity of HBV infection can appear in two forms: seropositive OBI, where serum HBV DNA is detectable and both anti-HBc/anti-hepatitis B surface (HBs) IgGs are positive, or seronegative OBI, where only HBV DNA is detectable in serum/or liver tissue, but anti-HBc IgG/anti-HBs IgGs are negative^5^. Patients undergoing maintenance hemodialysis (MHD) represent a susceptible population for hepatitis B virus (HBV) infection due to factors such as compromised immune response, frequent blood transfusions, prolonged MHD duration, shared dialysis equipment, repeated blood vessel punctures, and reduced responsiveness to vaccines^6–8^. Concurrently, individuals with OBI face an elevated risk of progressing to liver disease, a condition with potential transmission through dialysis, blood transfusion, and organ transplantation, leading to manifestations like explosive hepatitis and hepatocellular carcinoma^9^. Studies around the world have shown that the prevalence of HBV infection is higher in MHD patients than in the general population^10^. In addition, the prevalence of OBI in patients with MHD ranges from 0 to 58%, varies by country, and depends primarily on the prevalence of HBV infection in the general population of that country^11,12^.

The primary reason for OBI is the presence of mutations in the HBsAg gene, which is responsible for coding the surface antigen of the virus. The mutations can alter the structural arrangement of the protein, leading to low binding affinity with the monoclonal antibody used in commercially available HBsAg test kits^13,14^. Additionally, the mutations can result in the production of escape mutants that go undetected by the host's immune system^15^. Research has implied that the development of OBI is related to mutations in the S gene of the virus, particularly in the "a" determinant of the HBsAg^16^. Some mutations in the "a" determinant, including A159G, K160N, K122R, T126A, P127T, Q129H/R, L134S, K141E, P142S, D144A/E/V and G145R/A, have been shown to result in decreased HBsAg binding and failure of HBsAg tests^17–20^. The "a" determining cluster domain is a highly conserved region of HBsAg, located on the outer surface of the major hydrophilic region (MHR), where changes in amino acids can affect its hydrophilicity^21^. Although previous studies have suggested that some amino acid mutations in the “a” determinant may lead to decreased HBsAg binding and test failure, these findings have been limited by the small number of clones or patients analyzed and the unknown prevalence of naturally occurring mutation in OBI patients^22^. Additional studies are required to fully understand the underlying causes of OBI and to develop more efficient methods of diagnosis. Based on previous observations of mutations in the S genes Q129R, T131N, M133S, F134L, and D144E in MHD patients with OBI^23^, the current study aimed to perform transcriptome sequencing and in vitro functional analysis of the M133S mutation, which has not been reported in studies, have been carried out to study its role in the development of OBI.

## Materials and methods

### Reagents

The Fetal bovine serum (FBS), trypsin, streptomycin-penicillin, Dulbecco's modified Eagle's medium (DMEM), Opti-MEM, and Lipofectamine 2000 used for cell culture were obtained from Thermo Fisher. HEK293 cells were purchased from the Shanghai Institute of Biochemistry and Cell Biology and their authenticity was confirmed by Short Tandem Repeat (STR) analysis. Plasmids of wildtype or mutated S-gene were designed and synthesized for overexpression cloned in pcDNA3.1 vectors, with both HA- and GFP- tags. The kit for total RNA extraction was purchased from Foregene (Chengdu, China).The antibodies of PDI, TOMM20 and LAMP2 were purchased from Abcam Company, and the antibody of LC3 was obtained from Cell Signaling Technology Company.

### Construction of pEGFP-C1 small SP-HA overexpression vector (HBsAg (S))

The small SP-HA fragment (Hepatitis B virus (strain ayw, genotypes B) gene, NC:003977.2, synthesized by Hangzhou Youkang Biotechnology Company) was cloned into the pEGFP-C1 plasmid (VT1118, youbio) by using the restriction endonuclease SalI/BamHI.

Small SP-HA sequence:

atggagaacatcacatcaggattcctaggaccccttctcgtgttacaggcggggtttttcttgttgacaagaatcctcacaataccgcagagtctagactcgtggtggacttctctcaattttctagggggaactaccgtgtgtcttggccaaaattcgcagtccccaacctccaatcactcaccaacctcttgtcctccaacttgtcctggttatcgctggatgtgtctgcggcgttttatcatcttcctcttcatcctgctgctatgcctcatcttcttgttggttcttctggactatcaaggtatgttgcccgtttgtcctctaattccaggatcctcaacaaccagcacgggaccatgccggacctgcatgactactgctcaaggaacctctatgtatccctcctgttgctgtaccaaaccttcggacggaaattgcacctgtattcccatcccatcatcctgggctttcggaaaattcctatgggagtgggcctcagcccgtttctcctggctcagtttactagtgccatttgttcagtggttcgtagggctttcccccactgtttggctttcagttatatggatgatgtggtattgggggccaagtctgtacagcatcttgagtccctttttaccgctgttaccaattttcttttgtctttgggtatacattTACCCCTATGATGTGCCTGACTACGCA(HA)taa

**Figure 1:**
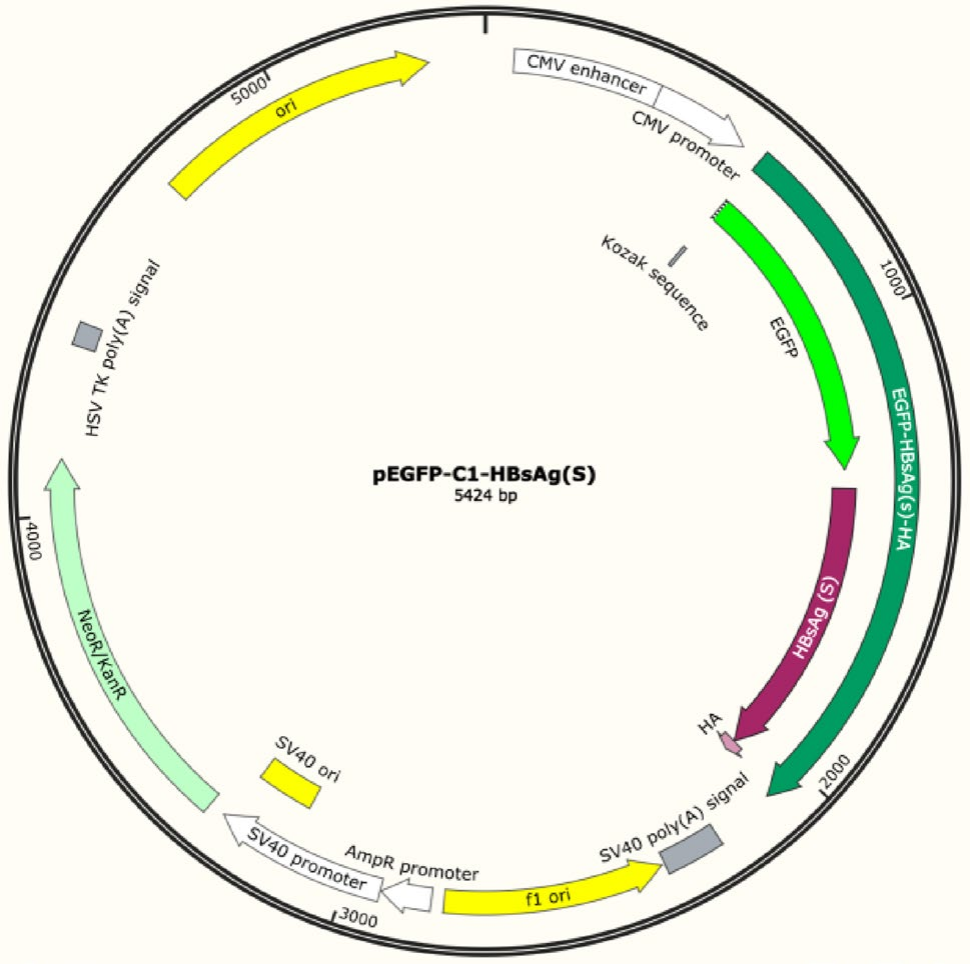
pEGFP-C1 small SP-HA overexpression vector (HBsAg (S)).

The completed plasmid structure is shown in the Fig. 1.

### Cell culture and transfection

HEK293 cells were maintained in DMEM medium supplemented with 10% FBS, streptomycin at 100 mg/mL, and penicillin at 100 U/mL concentrations. Cells were grown in a humidified atmosphere containing 5% CO_2_ at 37 °C. To overexpress the wildtype or mutated S gene, HEK293 cells were plated in six-well plates or 24-well plates and grown overnight with complete medium. For immunostaining, cells were transfected with the 1 μg plasmids in 24-well plates for 24 h, and the 3 μg plasmids were transfected for 48 h for transcriptomics. Transfection of plasmids was performed by Lipofectamine 2000 according to the manufacturer's recommendations. Transfected cells with the GFP vector served as controls.

### Immunostaining

For immunostaining, HEK293 cells were fixed with 4% paraformaldehyde for 10 min at room temperature followed by permeabilization with 0.25% Triton X-100 and blocking with 2% bovine serum albumin (BSA) in PBS for 1 h at 37 °C. Immunostaining was conducted using primary antibodies (diluted 1:500) and Alexa Fluor secondary antibody (diluted 1:1000), and 4′,6-diamidino-2-phenylindole (DAPI) at a concentration of 1.5 μg/μL for nuclear visualization. At least three fields from each stain were chosen randomly for analysis, and image acquisition was performed using a confocal imaging (S3000, HOOKE Instruments). The NIH ImageJ software was used to process the images.

### Western blotting

Cells were collected, proteins were extracted after cell lysis, separated using 10–15% sodium dodecyl sulfate–polyacrylamide gel electrophoresis gels, and transferred to nitrocellulose filter membranes. After blocking with 5% bovine serum albumin, the membranes were incubated with the corresponding primary and secondary antibodies. After processing with the Ultra High Sensitivity ECL Kit (HY-K1005, MCE, USA), the images were exposed, saved, and quantitatively analyzed using Image J software (ImageJ, https://imagej.net/ij/). β-actin was used as the loading control.

### Prediction software

Phyre2 online analysis software for tertiary structure prediction of S mutated proteins (http://www.sbg.bio.ic.ac.uk/phyre2). Once the amino acid sequence to be predicted has been input, select a strengthening analysis to give a three-dimensional map of the protein structure. Expasy online analysis software (http://www.expasy.org) is used to analyse the hydrophilicity and hydrophobicity of mutants of the S protein. Once the ProtScale analysis software (https://web.expasy.org/protscale/) is selected, the amino acid sequence and protein name are entered, and to select the hydrophilicity and hydrophobicity analysis to obtain the hydrophobicity score for each site as well as the hydrophobicity of the entire sequence.

### Transcriptomics

The RNA transcriptomics were performed with the help of Novogene (Tianjin, China). The RNA Nano 6000 Assay Kit of the Bioanalyzer 2100 system was used to assess the total amount and integrity of RNA. After qualifying the library, different libraries were pooled according to their effective concentration and the target amount of data off the machine, and then sequenced by the Illumina NovaSeq 6000. Fordata analysis, differentially expressed genes were identified through DESeq2 analysis by setting the significance threshold of P value less than 0.05 and fold-change greater than 2 or fold-change less than 0.5. For enrichment analysis, the differentially expressed genes were subjected to analysis using the DAVID Bioinformatics Resources v6.8 online server. Functional enrichment analysis was performed mainly using the Kyoto Encyclopedia of Genes and Genomes (KEGG) genetic function database^24–26^.

### Statistical analysis

All experiments were independently performed in triplicate at least three times. Statistical analyses were performed by GraphPad Prism 8.3.0. P values less than 0.05 were regarded as statistically significant (*p < 0.05, **p < 0.01, ***p < 0.001).

### Ethics statement

This study was approved by the Ethics Committee of Sichuan Provincial People’s Hospital. The protocol was carried out in accordance with relevant guideline and regulation.

## Results

### 1. Prediction of the potential mechanism of S protein mutation

**Figure 2:**
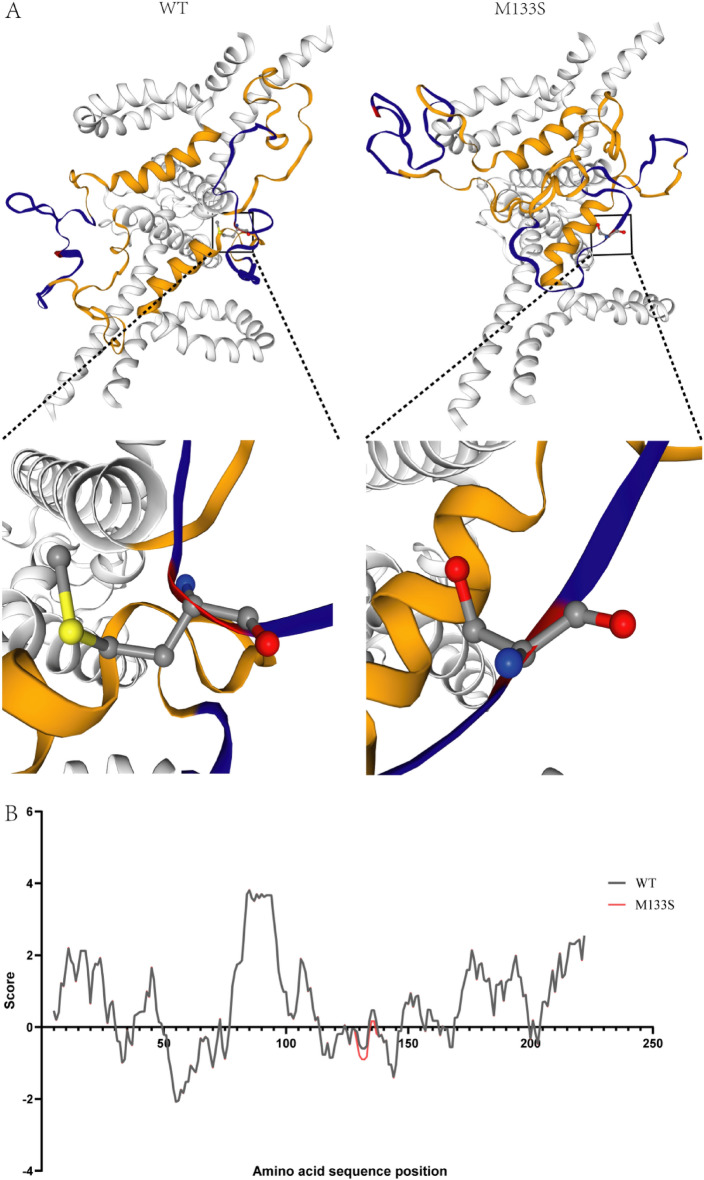
Structure prediction of S proteins. (**A**) Structure prediction of S protein wild type and M133S mutant. The blue area is the MHR region, the yellow area is the "a" antigenic determinant cluster, and the red area is the 133 site. (**B**) Hydrophilic characterization of S protein wild type and M133S mutant. The hydrophobicity score of each locus was determined using ExPASyProtScale online software. The black line is wild type and the red line is M133S.

The overall structure of the S-protein was predicted using Phyre2 software. As shown in Fig. 2A, 133 sites in the "a" antigenic determinant cluster located in the major hydrophilic region (MHR) were replaced by Ser, resulting in a more concentrated overall structure and a significant secretion disruption. The hydrophobic nature of the S-protein were then predicted using Expasy software (Fig. 2B, the original data can be found in Supplementary Information 1), and the presence of M133S transformed the site from hydrophobic to hydrophilic, affecting the hydrophilicity around the site and reducing the hydrophobicity of the MHR. Based on these predictions, we hypothesized that the M133S mutation may alter the nature and structure of S proteins and may lead to dysregulation of intracellular S protein aggregation and secretion.

### 2. S gene mutation transcriptomic analysis

**Figure 3:**
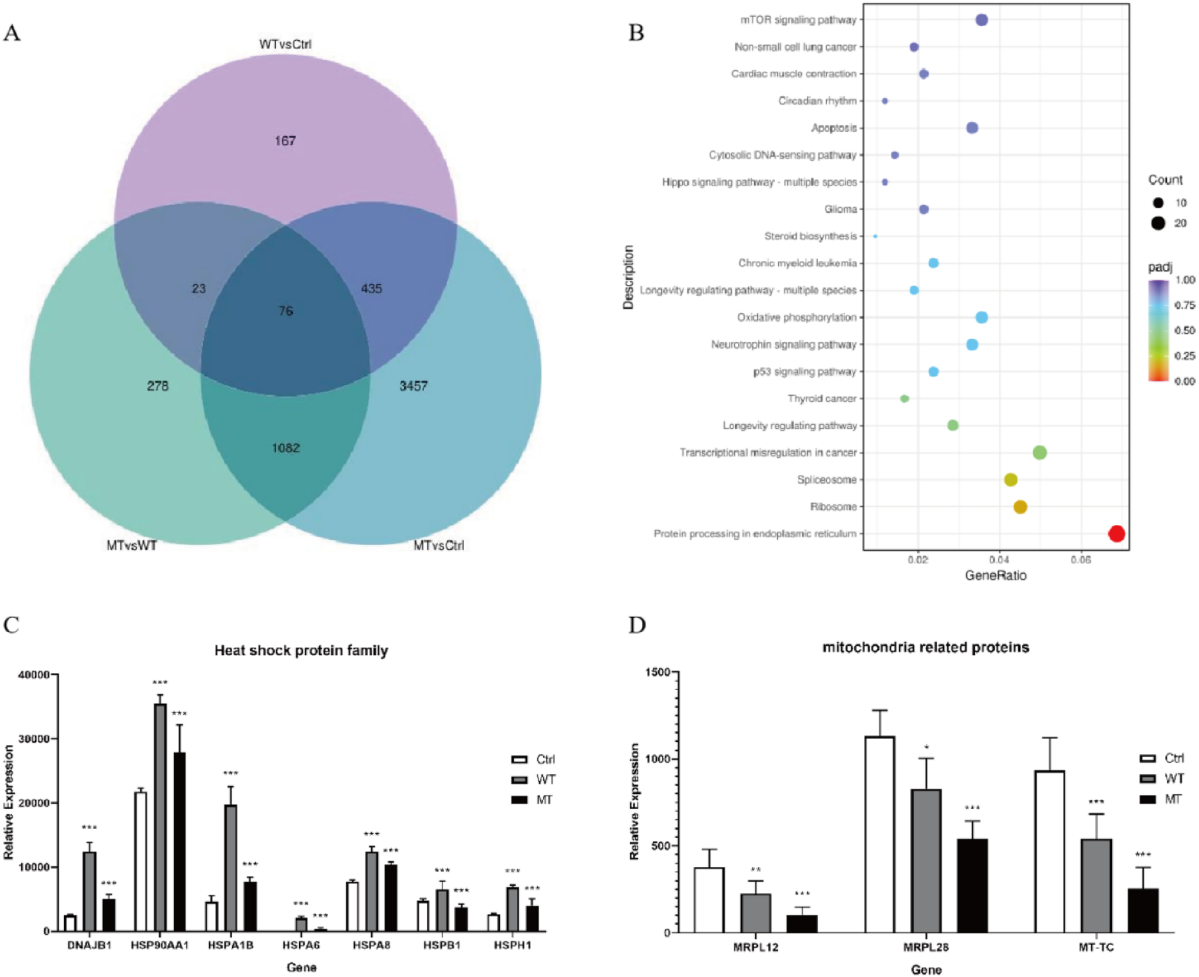
Transcriptomic analysis of S-gene mutations. Differentially expressed genes were analyzed by DESeq2 with a significance threshold of P value < 0.05. (**A**) Venn diagram of the intersection of differentially expressed genes in the control, wild-type, and mutant groups. Purple is the control group vs wild type, blue is the control group vs mutant, and green is wild type vs mutant. (**B**) KEGG enrichment plots of differentially expressed genes between the three groups. (**C,D**) Histograms of gene expression of the heat shock protein family and mitochondria-associated proteins between the three groups. Each assay was repeated three times (technical repeats) in three independent experiments (biological repeats). Compared with the control (Ctrl) group: ^*^P < 0.05, ^**^P < 0.01, ^***^P < 0.001.

HEK293 cells that had been successfully transfected with wild type and mutant S-gene plasmids were subjected to transcriptome sequencing, and genes differentially expressed between control (Ctrl), wild type (WT) and mutant (MT) groups were analysed by DESeq2. The Venn diagram (Fig. 3A) revealed a total of 76 differentially expressed genes that intersected between the three clusters. We performed an enrichment analysis and the KEGG enrichment map (Fig. 3B, the original data can be seen in Supplementary Information 2) showed that the differentially expressed genes were primarily enriched in the processing of proteins in the endoplasmic reticulum. As can be seen in Fig. 3C, D (The original data can be shown in Supplementary Information 3 and 4), gene expression of the heat shock protein family and proteins related to mitochondria were significantly different. There was a significant increase in the expression of heat shock protein family genes in both the wild type and mutant groups compared to the control group, although expression was significantly decreased in the mutant group relative to the wild type group. At the same time, the expression of mitochondrial-related protein-coding genes showed a decreasing trend in the wild type and mutant groups, while the lowest expression was observed in the mutant group. Through transcriptomic analysis, it can be inferred that mutations in the S gene alter the expression of endoplasmic reticulum-related proteins and mitochondrial related-proteins, which may affect protein maturation and mitochondrial function within the endoplasmic reticulum, leading to their accumulation in the endoplasmic reticulum and resulting in secretion disorders.

### 3. In vitro functional analysis of S gene mutation

**Figure 4:**
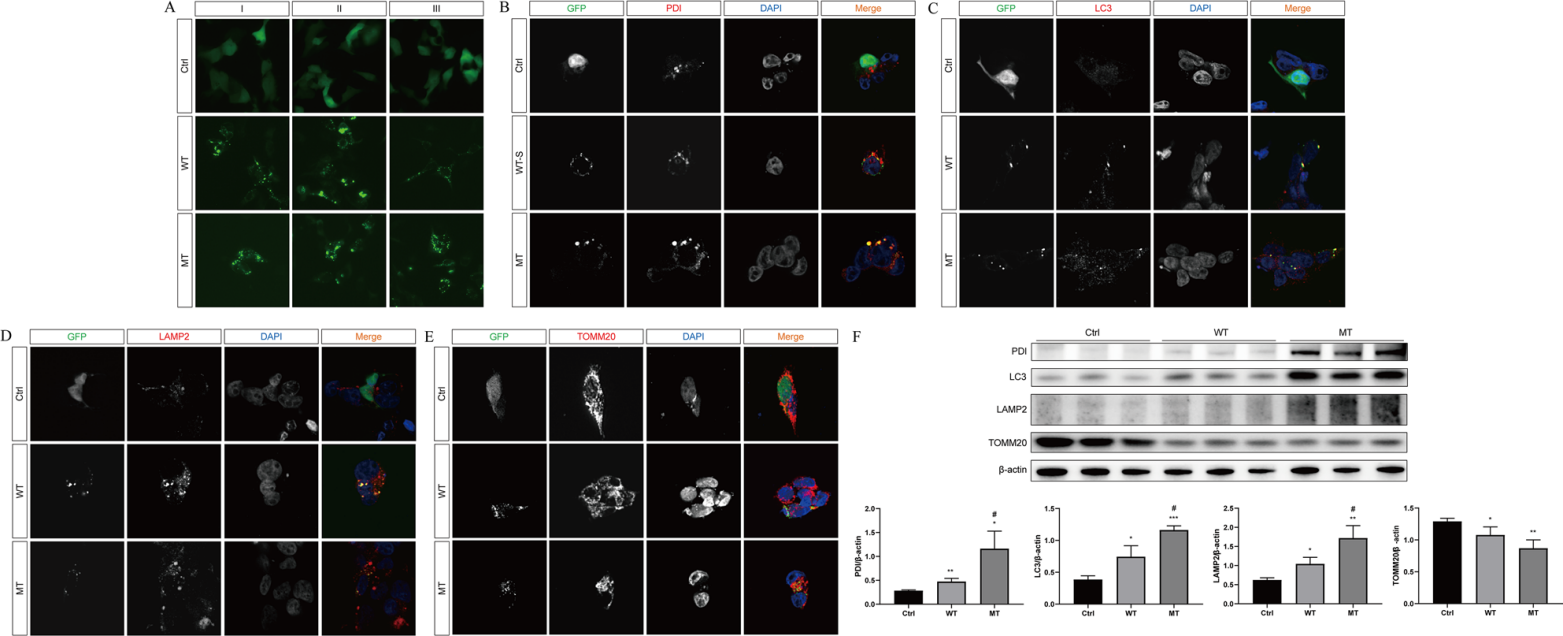
Functional analysis of S gene mutation in vitro. (**A**) Expression of fluorescent tag protein GFP in HEK293 cells transfected with S-gene wild-type and M133S mutant plasmids. (**B–E**) Expression of PDI, LC3, LAMP2, and TOMM20 proteins was detected by immunofluorescence in HEK293 cells transfected with S-gene wild-type and M133S mutant plasmids. GFP is in green, related proteins in red, and DAPI in blue. (**F**) Expression and quantification of proteins. Each assay was repeated three times (technical repeats) in three independent experiments (biological repeats). Compared with the control (Ctrl) group: ^*^P < 0.05, ^**^P < 0.01, ^***^P < 0.001; compared with wild-type plasmid group: ^*#*^*P* < 0.05, ^*##*^*P* < 0.01, ^*###*^*P* < 0.001.

The S gene wild-type and M133S mutant plasmids were transfected into HEK293 cells and the transfection was observed by fluorescence microscopy. As shown in Fig. 4A, the control, wild-type, and mutant groups all showed expression of the fluorescent tag protein GFP, indicating that the plasmid was successfully transfected into the cells and expressed. Immunofluorescence staining and protein blotting were used to detect the localization and expression of the associated proteins. The S proteins in the wild-type and mutant groups were co-localized and expressed with the endoplasmic reticulum-associated protein PDI (protein disulfide isomerase, which detects endoplasmic reticulum stress markers), suggesting that the S proteins were localized and contained in the endoplasmic reticulum. Meanwhile, the expression of PDI was significantly increased in the wild-type and mutant groups compared with the control group, while the highest expression level was observed in the mutant group (Fig. 4F). Similarly, the expression of autophagy markers LC3 (microtubule-associated protein light chain 3) and LAMP2 (lysosomal-associated membrane protein 2) showed similar trends among the three groups. The expression of LC3 and LAMP2 increased in both the wild-type and mutant groups, with the highest expression observed in the mutant group (Fig. 4F). In addition, the S protein of the wild-type and mutant groups was also co-localized and expressed with LC3 and LAMP2, clearly demonstrating the expression of S protein in autophagosomes and lysosomes (Fig. 4C, D). Similarly, the S protein of the wild-type and mutant groups was co-localized and expressed with TOMM20 (mitochondrial outer membrane translocation enzyme 20), indicating the expression of S protein in mitochondria. The expression of TOMM20 protein in both wild-type and mutant groups decreased, and the expression in the mutant group was significantly lower than that in the wild-type group (Fig. 4E, F, the original data can be found in Supplementary Information 5 and 6). Based on the above results, we predict that the M133S mutation in the S gene is expressed as a defective and misfolded protein that accumulates and exhibits impaired secretion in the endoplasmic reticulum, leading to endoplasmic reticulum stress, triggering mitochondrial autophagy and recruiting lysosomes to fuse with autophagosomes, resulting in mitochondrial clearance.

## Discussion

OBI as a specific form of HBV infection is characterized by the presence of replication-competent HBV DNA (usually less than 200 IU/ml) in the liver of individuals with negative results on currently available HBsAg assays^27^. The classification of OBI as seropositive or seronegative is based on whether antibodies to the core antigen of HBV (anti-HBc) and/or anti-HBsAg (anti-HBs) are detected in the serum, with seropositive OBI accounting for about 80% of all cases of OBI^28^. Chronic and persistent OBI infections have been reported throughout the world, and their prevalence varies across geographic regions, populations, and genotypes^29,30^. Infection with OBI can be transmitted by blood transfusion and organ transplantation, including liver transplantation, and the clinical outcome of OBI is not known, and most of the results suggest that OBI is a significant risk factor for accelerated liver disease progression and the development of cirrhosis and hepatocellular carcinoma^31^.The molecular mechanism of OBI is not completely understood, and several possible mechanisms have been proposed, including mutations in the HBV gene resulting in undetectable HBsAg commercial assays; robust host suppression of HBV replication and transcription as well as immune surveillance; and coinfection with other viruses such as hepatitis C virus (HCV)^32,33^. Indeed, it has been reported that deletion of HBsAg in serum can be associated with mutations in the HBV-borne gene S^34–36^. Our preliminary study identified mutations in the S genes Q129R, T131N, M133S, F134L, and D144E in 7 serologically positive patients with MHD in combination with OBI, and no mutations in M133S have been reported to date. Thus, in the present study, transcriptome sequencing and in vitro functional analysis were carried out to investigate the role of the M133S mutation in the development of OBI.

HBV is a 42 nm enveloped DNA virus with a circular partially double stranded DNA genome of 3.2 kb in size^37^. L, M, and S proteins are encoded by the preS/S ORF^38^. The S protein has been shown to be involved not only in the secretion of HBV viral particles but also in viral infectivity^39^. The aa99–169 region of HBsAg is the MHR, which contains the major conformational epitopes exposed to the outer surface of the viral particles^40^. The MHR has a relatively conserved "a" determinant cluster (aa 124–147) that is a target for neutralization of the B cell response^41^. Amino acid substitutions in the MHR region can cause conformational changes in the epitope, thereby affecting the hydrophilicity of S proteins and leading to changes in protein properties^42^. We found that the mutated 133 is located in the "a" region of the MHR antigen determinant cluster. Previous studies have found that M133T, M133L, and M133T/Q129R have slight effects on virus transcription, replication, HBeAg secretion, and immune detection^43,44^. M133L induced intracellular retention of S protein, reducing the extracellular levels of viral DNA and S protein, while M133T did not alter the extracellular viral DNA level, but the S protein level decreased. At the same time, the M133T/Q129R double mutant partially rescued the secretion of viral particles but induced a more pronounced S protein secretion deficiency. Through protein structure and hydrophilicity prediction, it was found that the S protein Met at 133 was replaced by a more hydrophilic Ser, causing a conformational change at this site, thereby affecting the overall structure and function. The M133S mutation of the S protein alters the characteristics and structure of the S protein, leading to disruption of intracellular S protein aggregation and secretion.

We have inferred the properties of the amino acids in the MHR region that affect S-protein synthesis and expression from the results of protein structure and hydrophilicity prediction, but the mechanism by which the M133S mutation leads to OBI is not fully understood. In the present study, through transcriptome sequencing and enrichment analysis of HEK293 cells successfully transfected with mutant plasmids from the S gene, our analysis revealed that the differentially expressed genes were primarily enriched for protein maturation in the endoplasmic reticulum. The endoplasmic reticulum (ER) is a major organelle that plays a major role in the synthesis of proteins, membrane proteins, and lipids and is especially important in the proper folding and modification of proteins that are secreted from the body^45^. ER stress occurs when the accumulation of unfolded or misfolded proteins in the ER outstrips the ability of the protein to fold, triggering the unfolded protein response (UPR)^46^. Infection with Hepatitis B virus induces the synthesis of large quantities of viral proteins, leading to protein overload in the ER^47^. Specific mutations in the S gene may result in defective secretion of S protein, leading to its accumulation and aggregation in the endoplasmic reticulum of hepatocytes, disrupting endoplasmic reticulum homeostasis and leading to endoplasmic reticulum stress and hepatocyte injury^48,49^. Specifically, differential gene analysis revealed that expression of genes in the heat shock protein family was upregulated following viral infection of cells. At the same time, viruses with the M133S mutation resulted in increased expression of genes for the heat shock protein family, but to a lesser extent than normal intact viruses. The heat shock family of proteins (HSP) consists of highly conserved molecules whose primary role is to have a role in promoting the folding and assembly of proteins. As a component of the UPR, heat shock proteins have also been shown to play an important role in defense against the accumulation of intracellular misfolded proteins^50^. In the presence of large quantities of viral proteins within the ER, heat shock proteins dissociate from membrane proteins in the ER lumen, resulting in the activation of the UPR signaling pathway^51^. Plasmids bearing S genes were expressed in the endoplasmic reticulum surrounding the nucleus and secreted into the cytoplasm upon entry into the cell. Meanwhile, immunofluorescence staining and protein blotting showed enhanced expression of PDI protein, a marker used to detect endoplasmic reticulum stress, showing activation after transfection of cells with the mutant S gene M133S plasmid^52^. In addition, we found that the defective S protein was located within the endoplasmic reticulum and could not be secreted into the cytoplasm. Based on these results, we hypothesized that after the M133S mutation in the S gene, a large number of defective proteins accumulate in the endoplasmic reticulum, leading to endoplasmic reticulum stress, heat shock protein dissociation, UPR activation, and ultimately impaired S protein secretion.

ER accumulation in S mutants activates other cellular signals, including apoptosis and cellular mitochondrial autophagy, in addition to ER stress^53^. Mitochondrial autophagy is a selective form of autophagy that targets damaged mitochondria. Mitochondrial autophagy maintains cellular homeostasis by removing defective mitochondria^54^. It has been reported that certain viruses can directly or indirectly trigger mitochondrial autophagy, thereby weakening the innate immune response and allowing the virus to persist in infection^55,56^. Hepatitis B virus can eliminate damaged mitochondria; misfolded proteins inhibit apoptosis by activating mitochondrial autophagy to promote viral survival^57^. Autophagy forms autophagosomes that can fuse with lysosomes to form autolysosomes in which the cellular waste contained therein is degraded^58^. HBx in HBV has been shown to induce substantial mitochondrial autophagy^59^. Our fluorescent staining and protein blotting showed that S-protein mutants were present in autophagosomes and lysosomes and that there was increased expression of the autophagy marker LC3 and the lysosomal membrane protein LAMP2 protein. LAMP2 is not only one of the important components of the lysosomal membrane but also plays an important role in autophagy. It can participate in protein synthesis of the lysosomal-endogenous pathway and lysosome-vesicle fusion and is a key regulator of autophagy^60^. It can be hypothesized that defective S proteins may induce cellular autophagy and activate lysosomes. In addition, analysis of transcriptome sequencing data showed that transfection of wild-type and mutant plasmids with the S gene significantly reduced the expression of mitochondria-associated proteins, with the lowest expression in the mutant group. In immunofluorescence and protein blotting results, S proteins were also present in mitochondria and the expression of the mitochondria-associated protein TOMM20 was significantly reduced. Based on the above speculation, S protein mutants activate mitochondrial autophagy and induce selective autophagic degradation of damaged mitochondria. In addition, the defective S proteins are localized and contained in autophagosomes and lysosomes, suggesting that S protein mutants induce mitochondrial autophagy by enhancing the fusion of autophagosomes with lysosomes. We will explore the specific mechanisms in future research endeavors.

Our research has some limitations. Firstly, the specific mechanism of S gene mutations has not been thoroughly studied and fully validated. Due to the limitations of DESeq or in the filtering stage, qPCR can be added for validation. To clarify its specific mechanism, validation methods such as lysosomal activity detection can be used, and animal experiments can be conducted under strict conditions. This will be a part of our future research. In addition, there are multiple mutation sites in the S gene, indicating that multiple site mutations may jointly participate in the occurrence of OBI. We will continue to conduct functional research on multiple S gene mutations and delve deeper into their pathogenesis.

In summary, the S gene M133S is expressed as a defective and misfolded S protein upon mutation. Defective S proteins may impair endoplasmic reticulum secretion and accumulation, leading to endoplasmic reticulum stress, which subsequently triggers autophagy and recruits a large number of lysosomes to fuse with autophagosomes, leading to mitochondrial clearance. This study preliminarily reveals the potential mechanism of M133S Mutation in the “a” Determinant of HBV S Gene in the development of OBI in MHD patients, which provides a new direction for the diagnosis and treatment of OBI in MHD patients.

### Supplementary Information

Supplementary Information 1.

Supplementary Information 2.

Supplementary Information 3.

Supplementary Information 4.

Supplementary Information 5.

Supplementary Information 6.

## Data Availability

All data generated or analysed during this study are included in this published article and its supplementary information files.
