# Supplementary material for: M133S mutation possibly involve in the ER stress and mitophagy pathway in maintenance hemodialysis patients with occult hepatitis B infection
Source: Sci Rep. 2024 Jun 17;14:13981. doi: 10.1038/s41598-024-64943-3 (PMC11183135; doi:10.1038/s41598-024-64943-3)

Original image

Figure 3-A
Ctrl-I

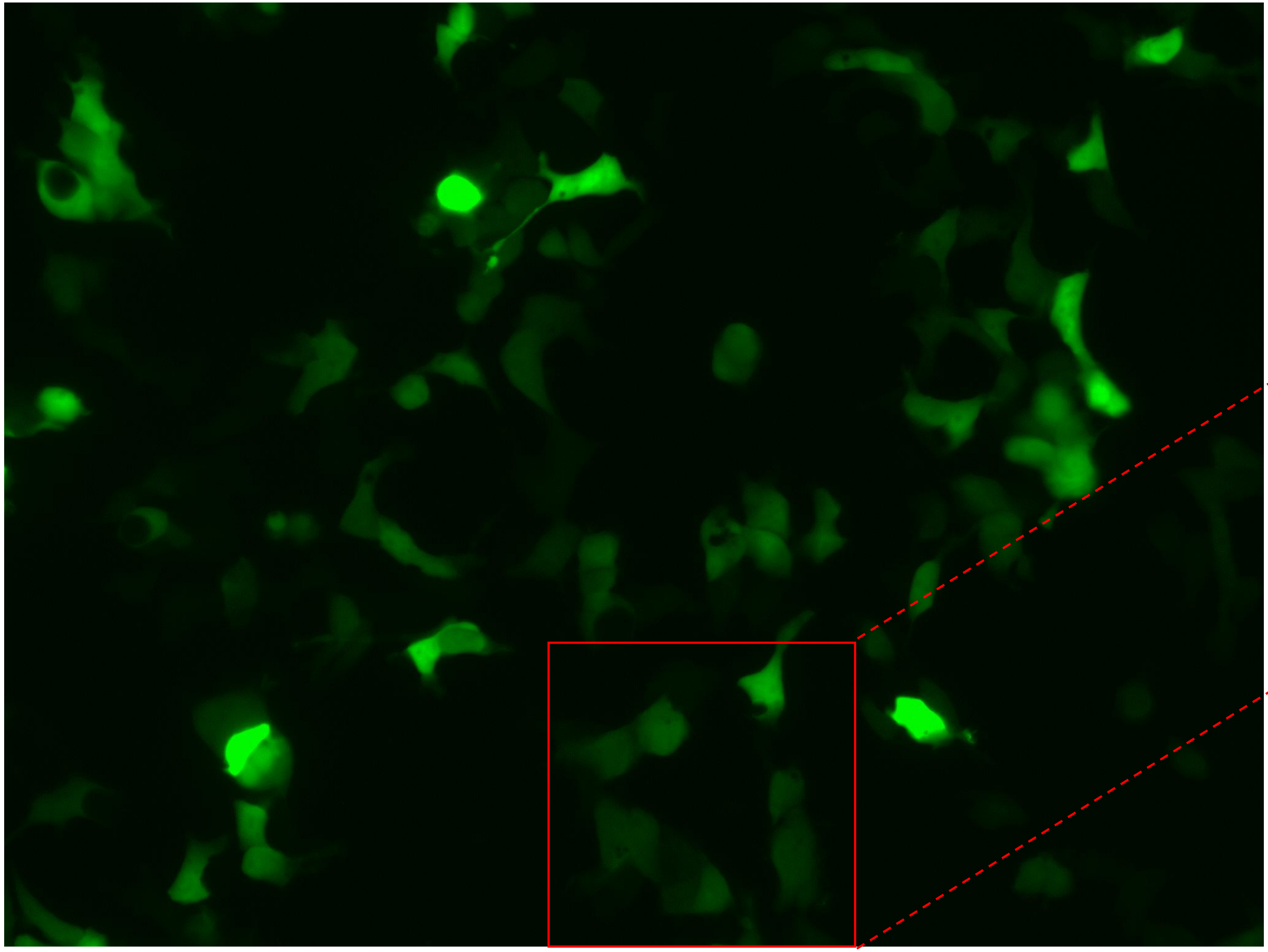

Image in the manuscript

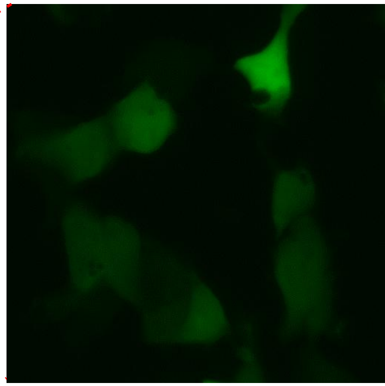

Figure 3-A
Ctrl-II

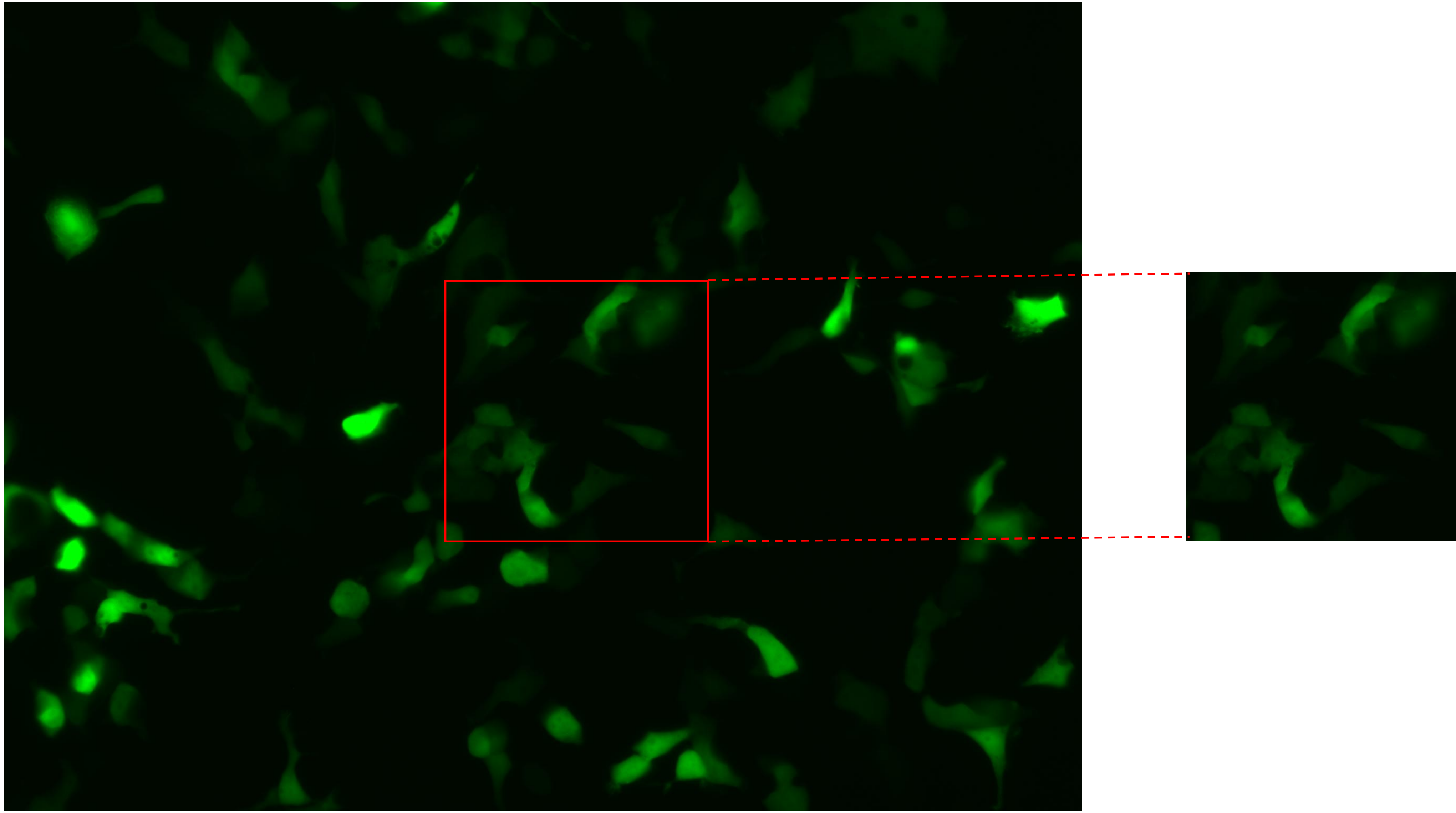

Figure 3-A
Ctrl-III

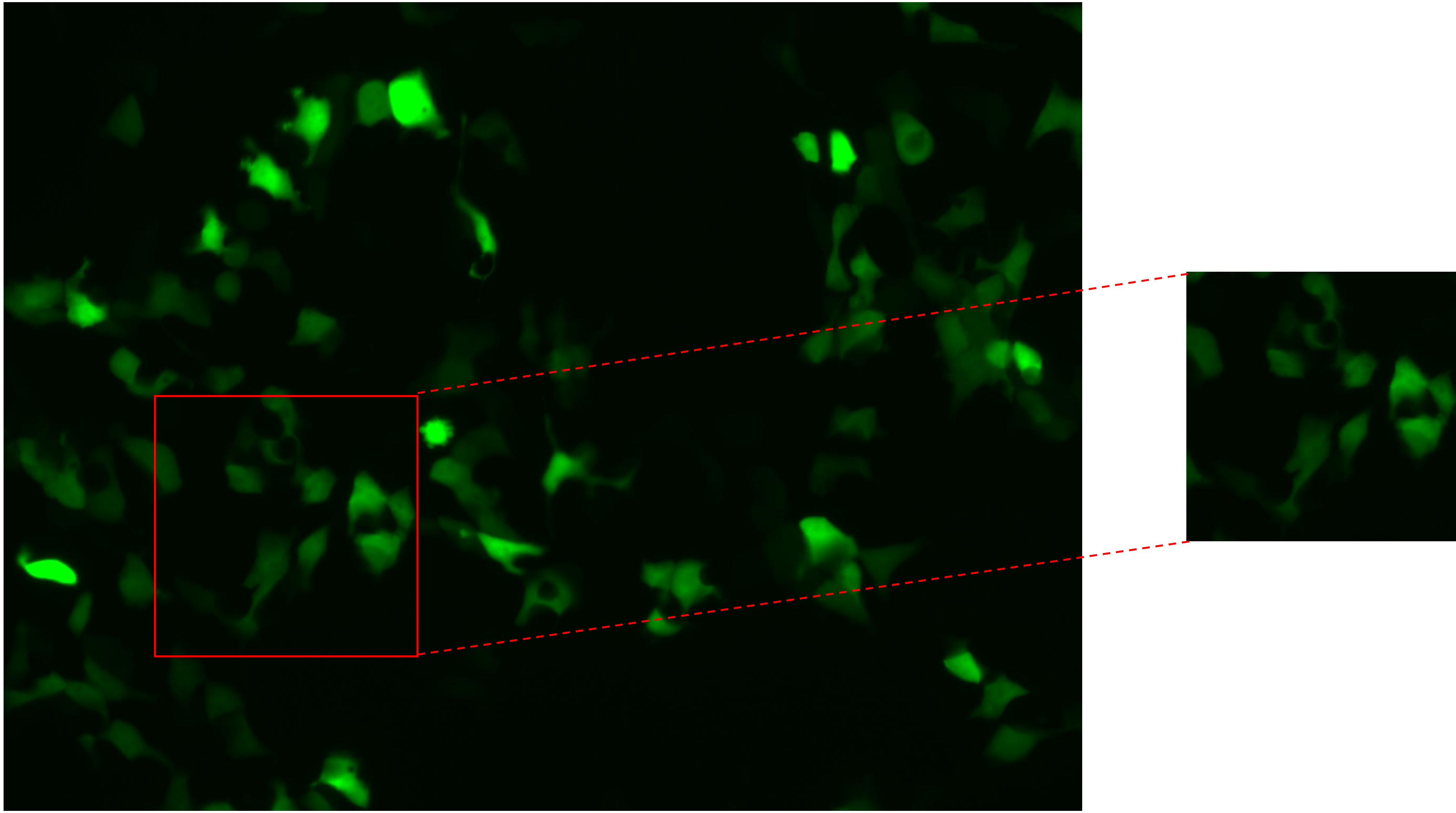

Figure 3-A
WT-I

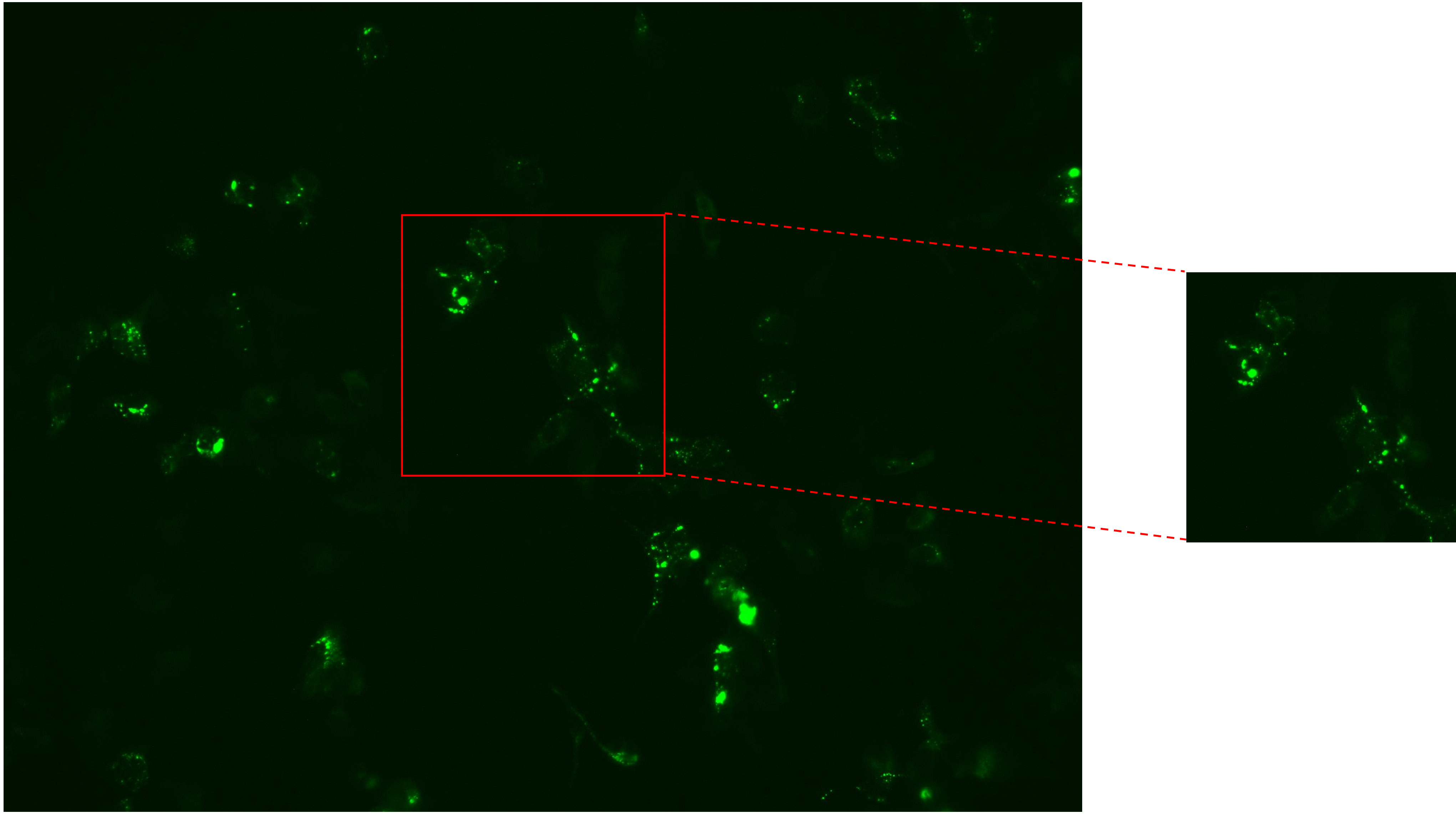

Figure 3-A
WT-II

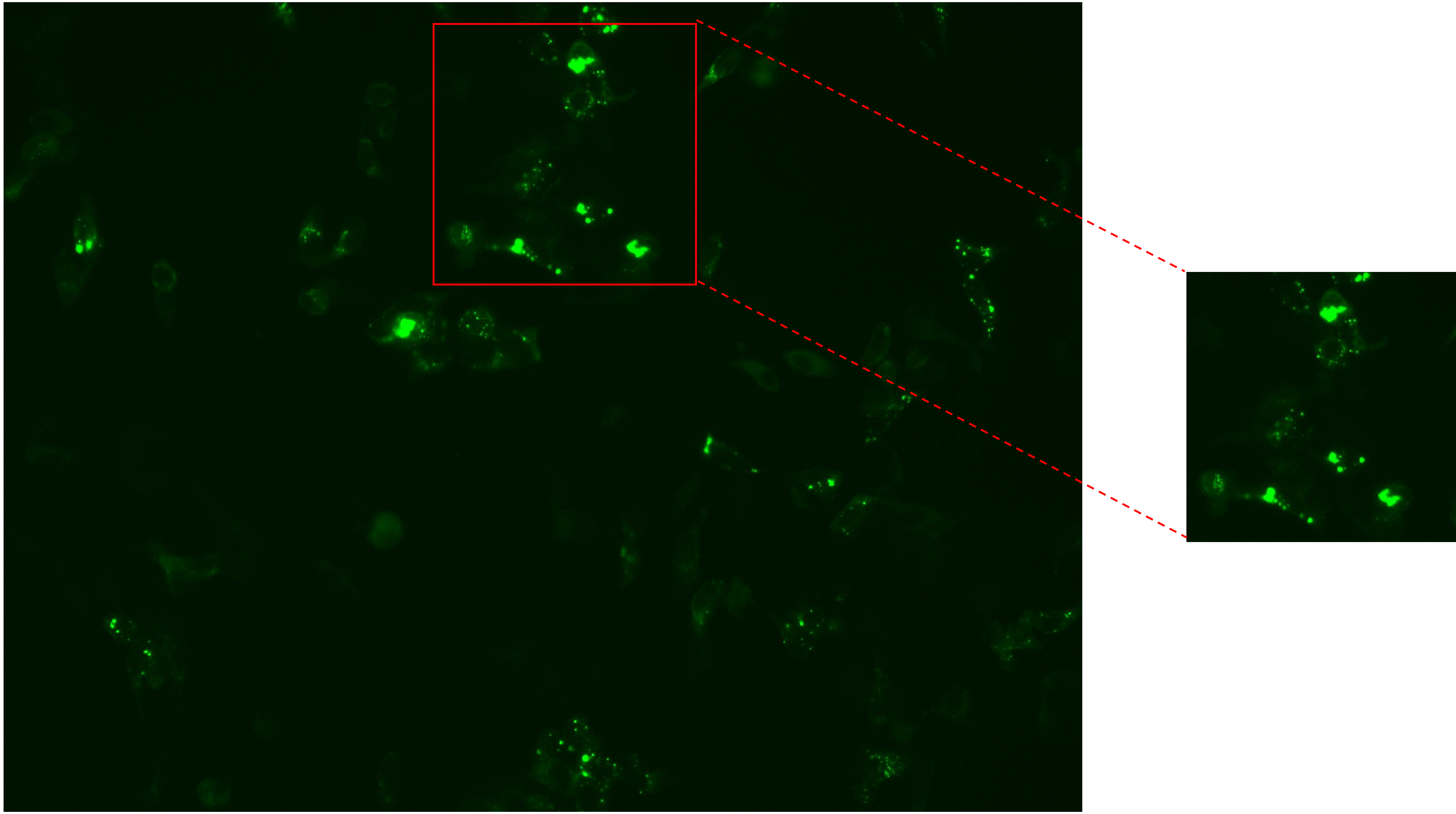

Figure 3-A
WT-III

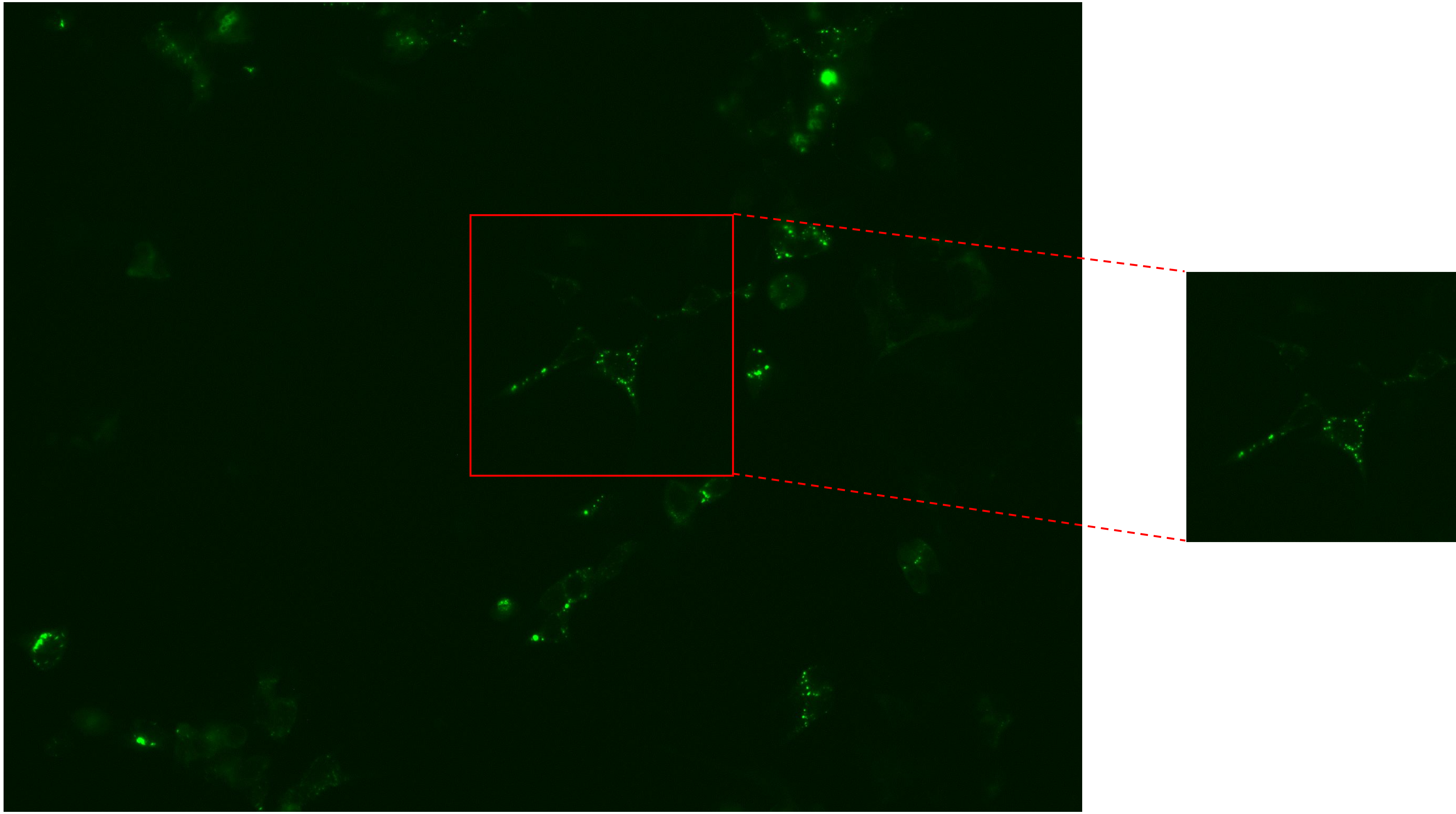

Figure 3-A
MT-I

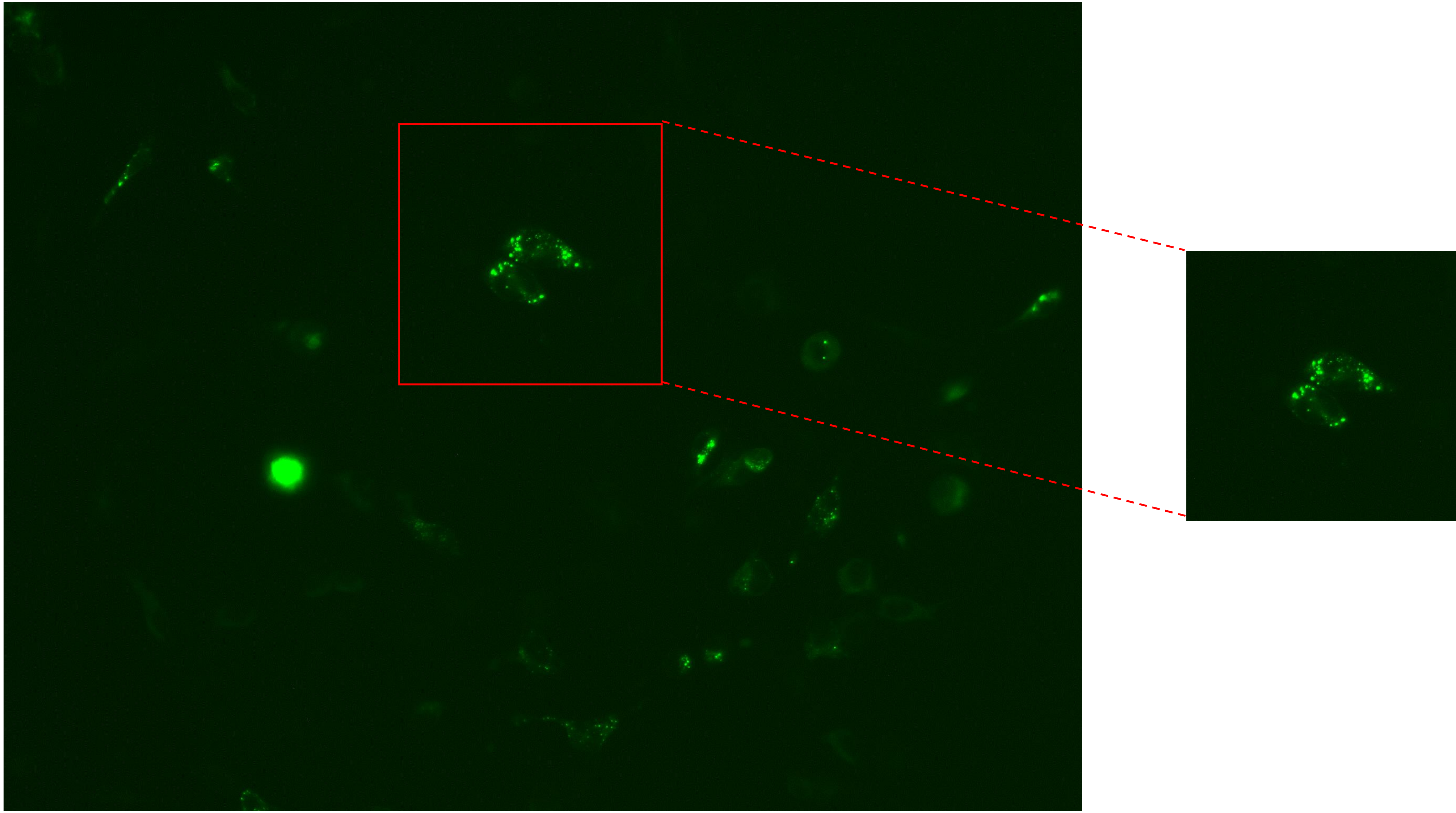

Figure 3-A
MT-II

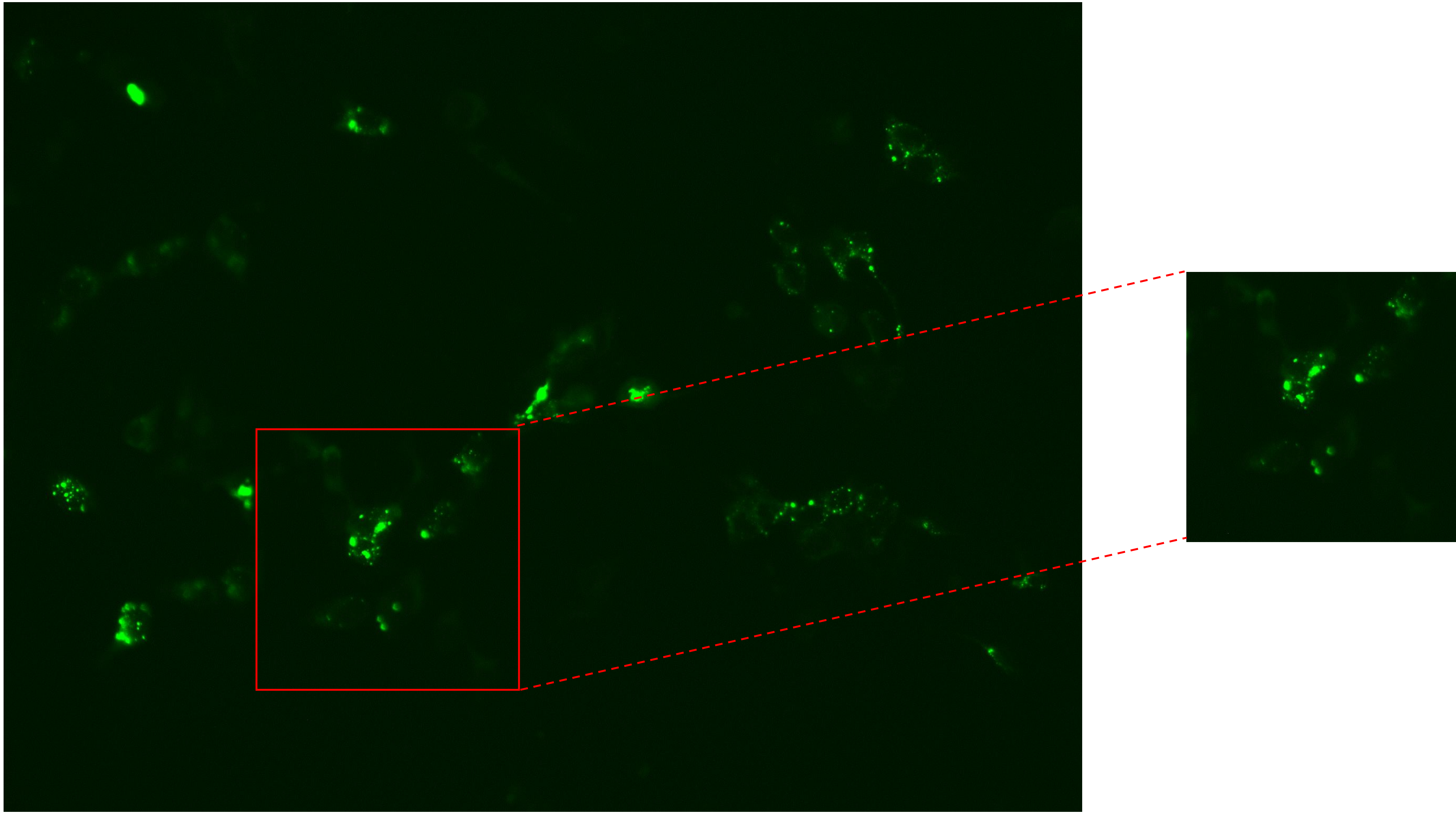

Figure 3-A
MT-III

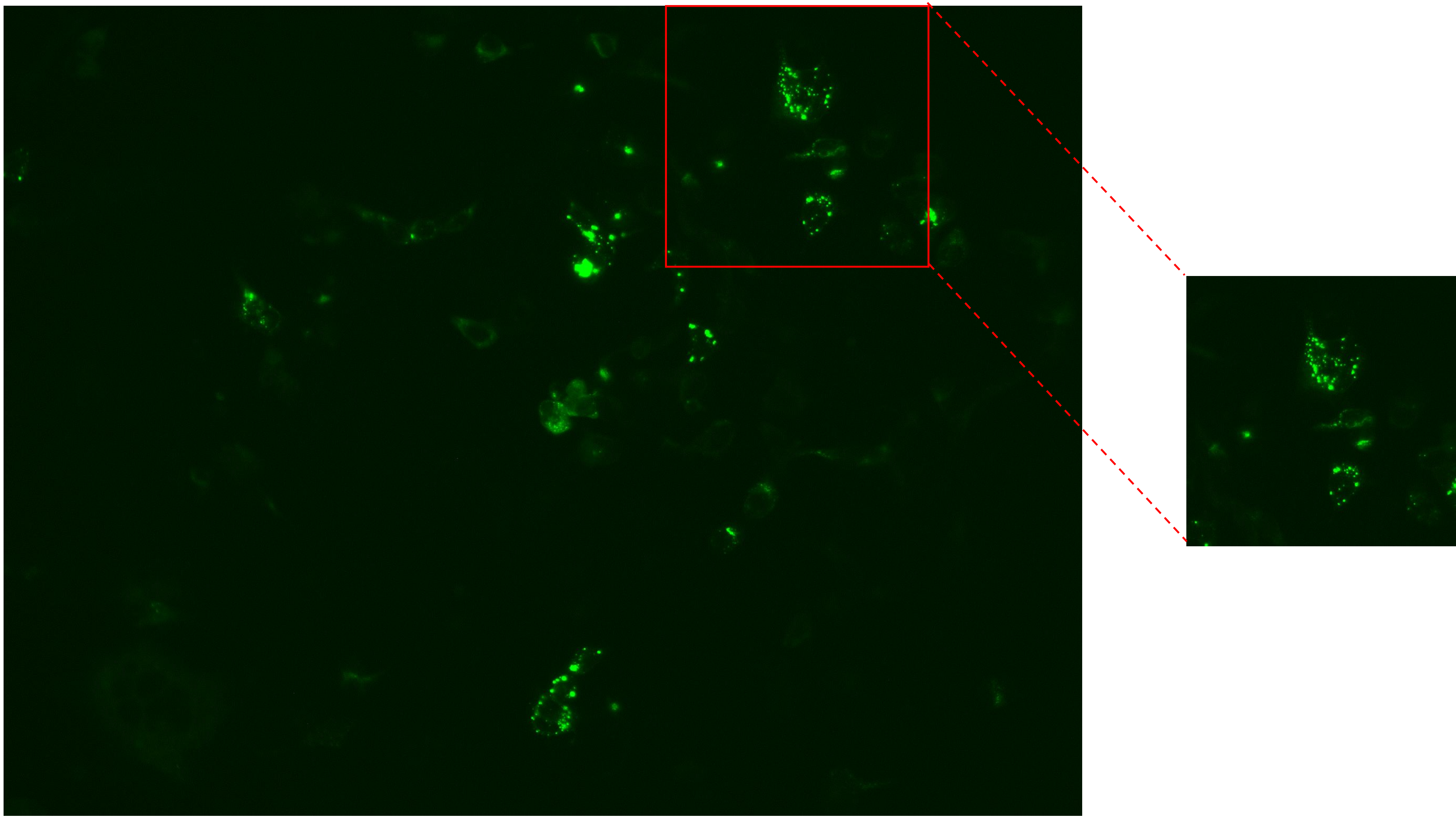

Figure 3-B
Ctrl-GFP

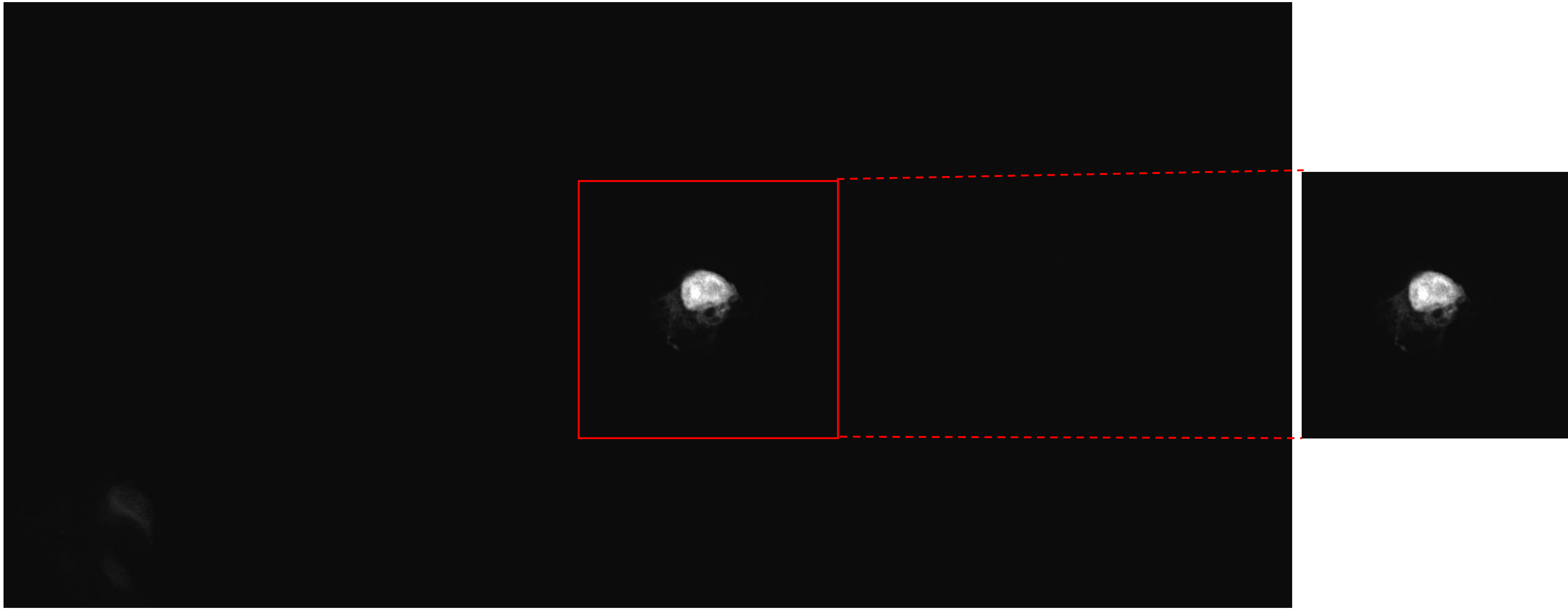

Figure 3-B
Ctrl-PDI

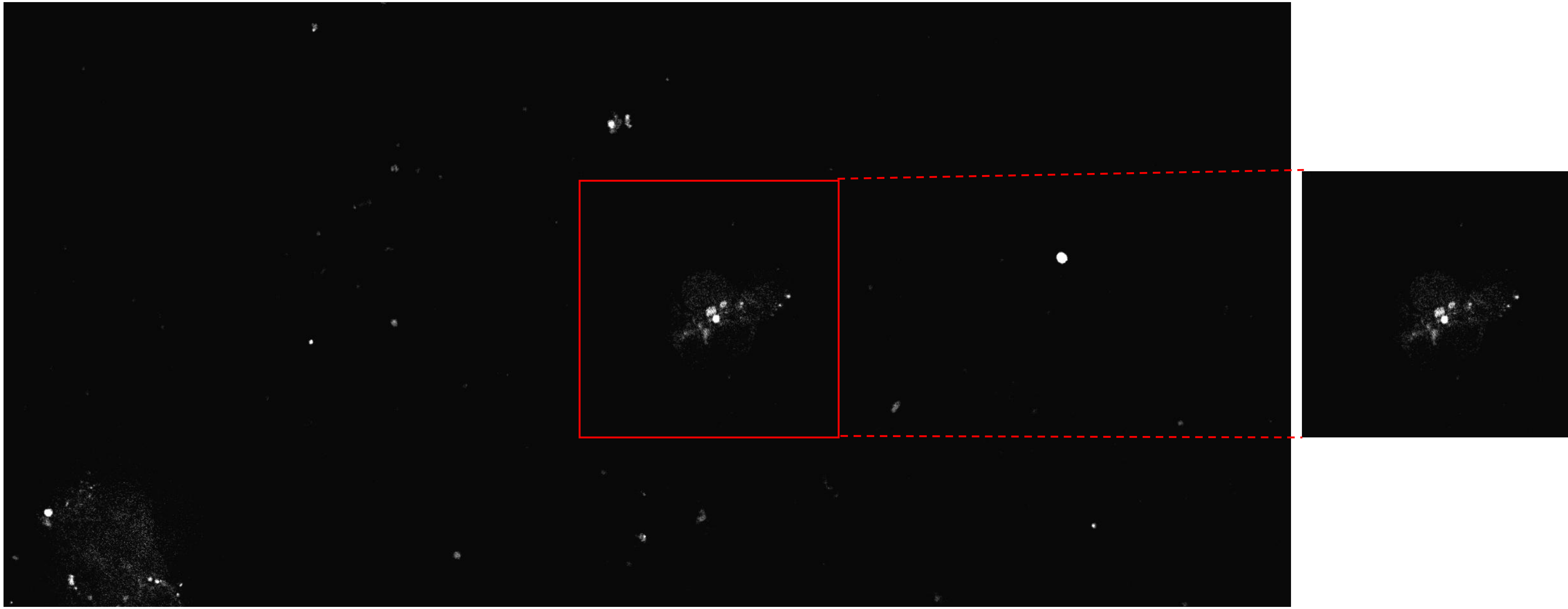

Figure 3-B
Ctrl-DAPI

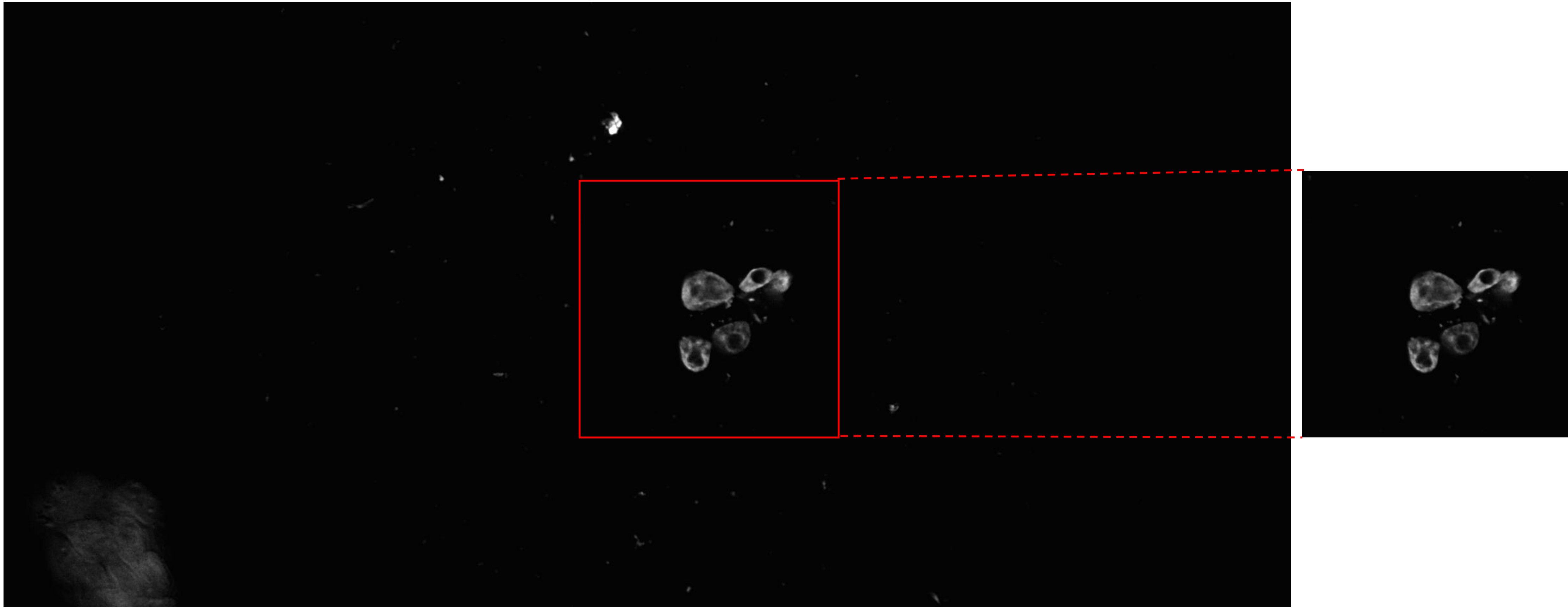

Figure 3-B
Ctrl-Merge

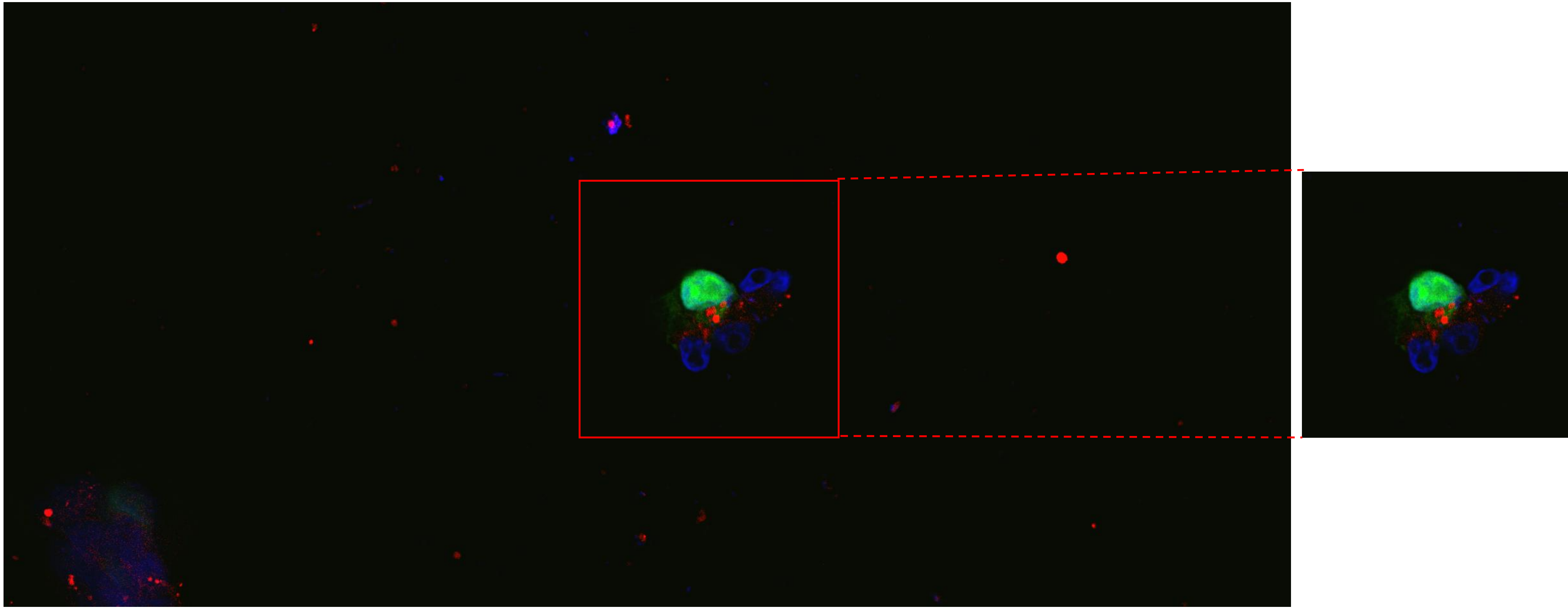

Figure 3-B
WT-GFP

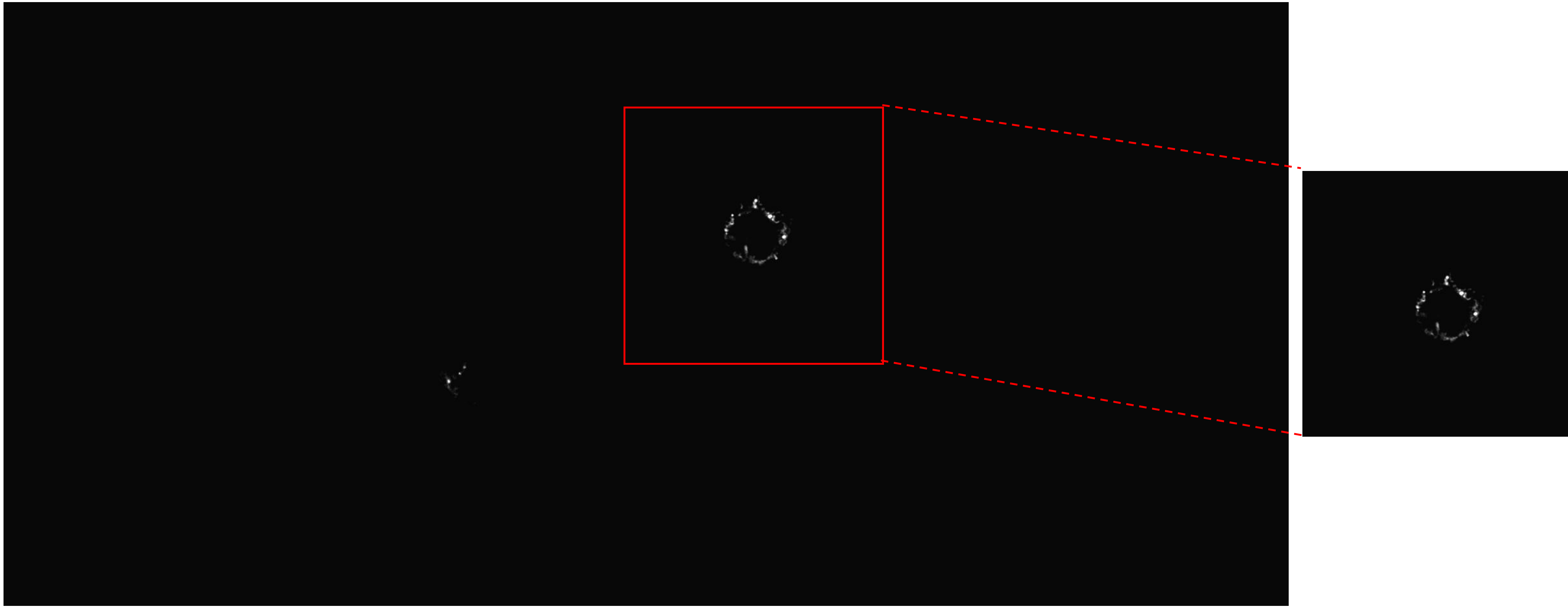

Figure 3-B
WT-PDI

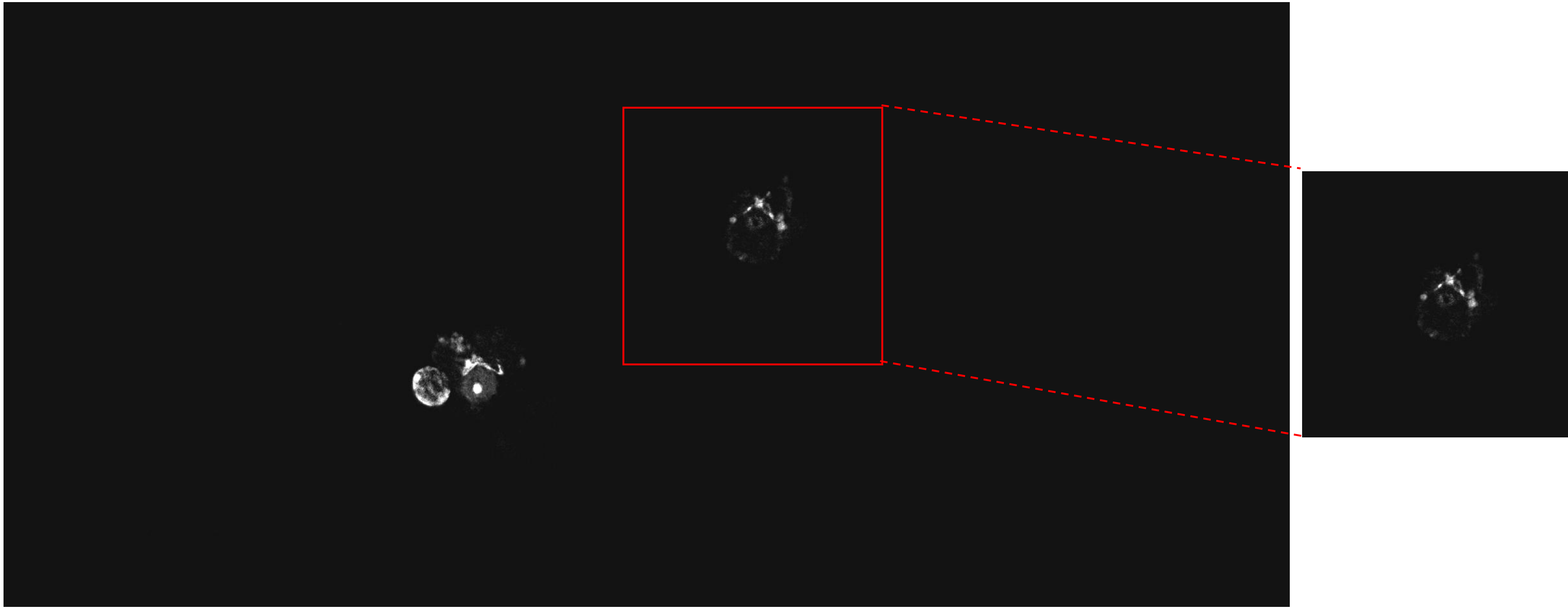

Figure 3-B
WT-DAPI

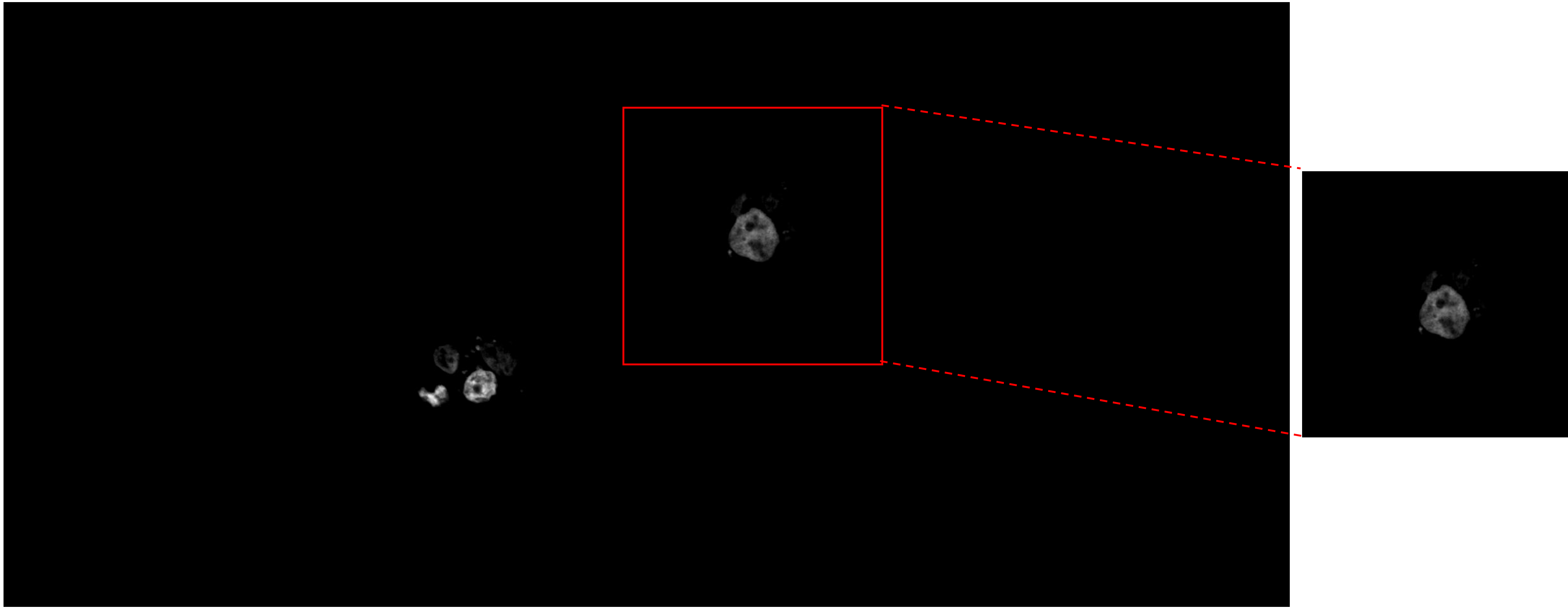

Figure 3-B
WT-Merge

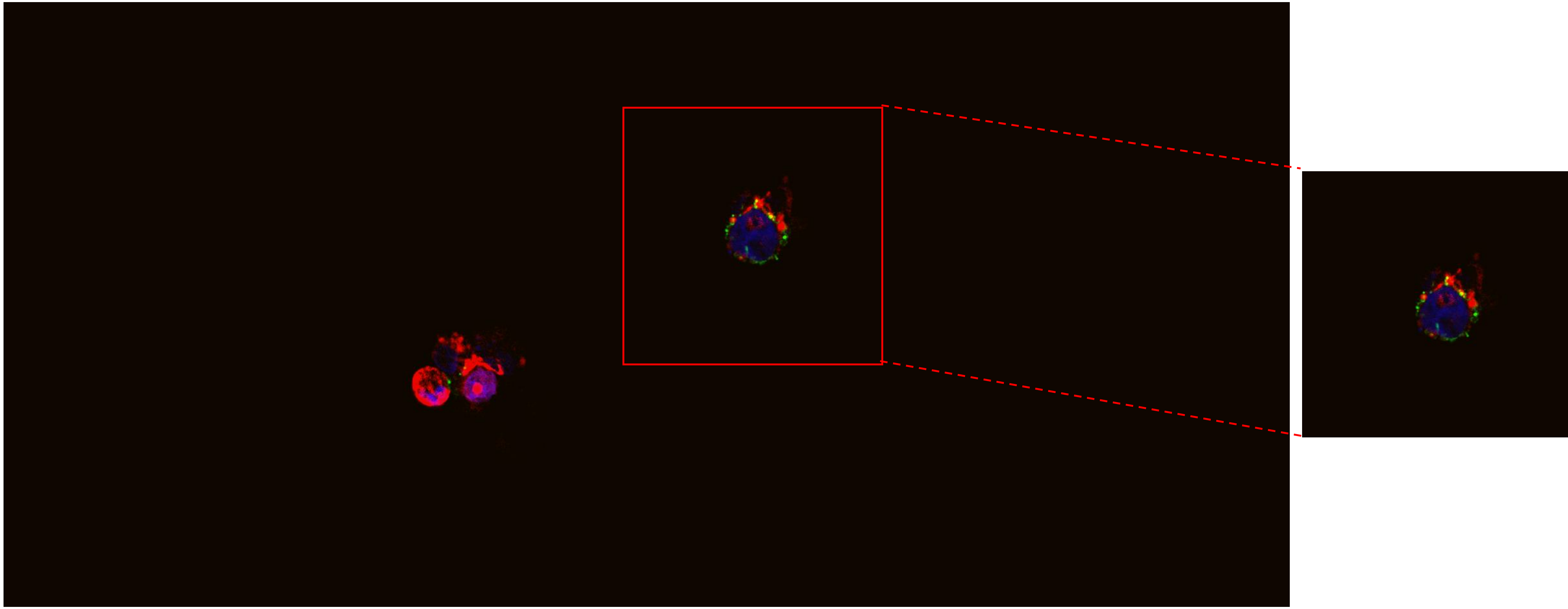

Figure 3-B
MT-GFP

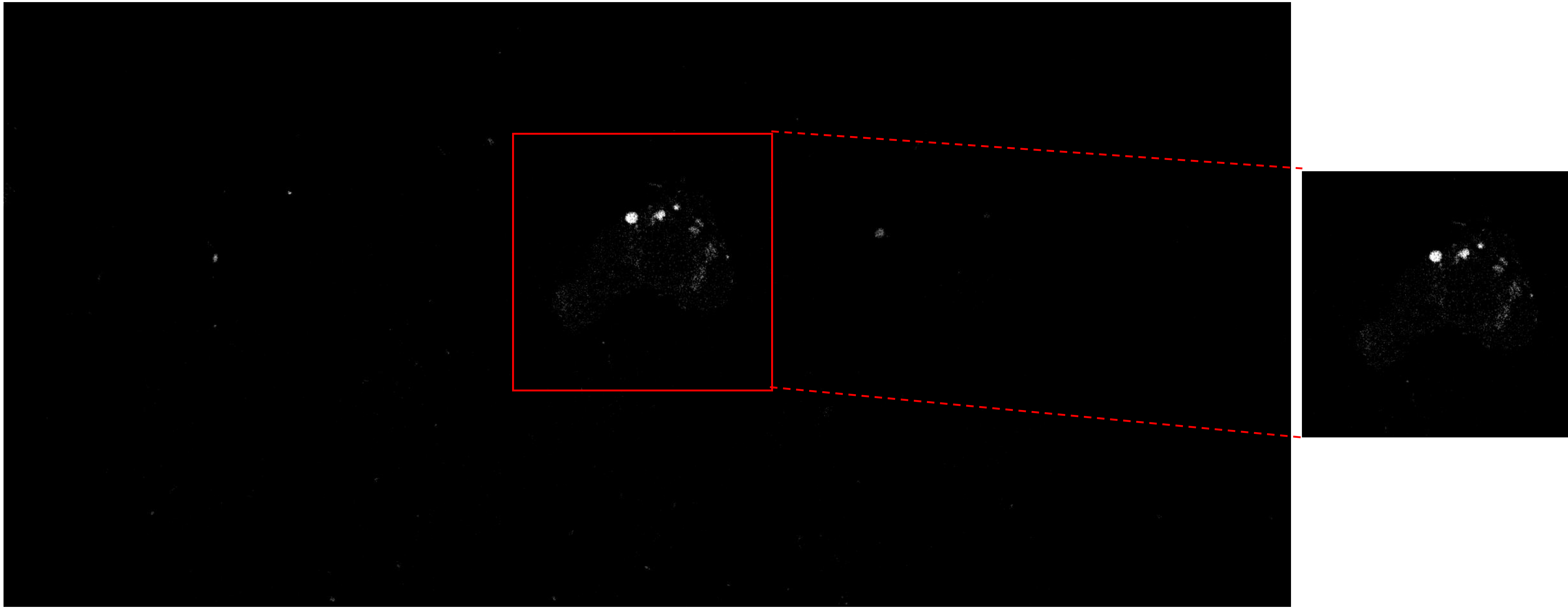

Figure 3-B
MT-PDI

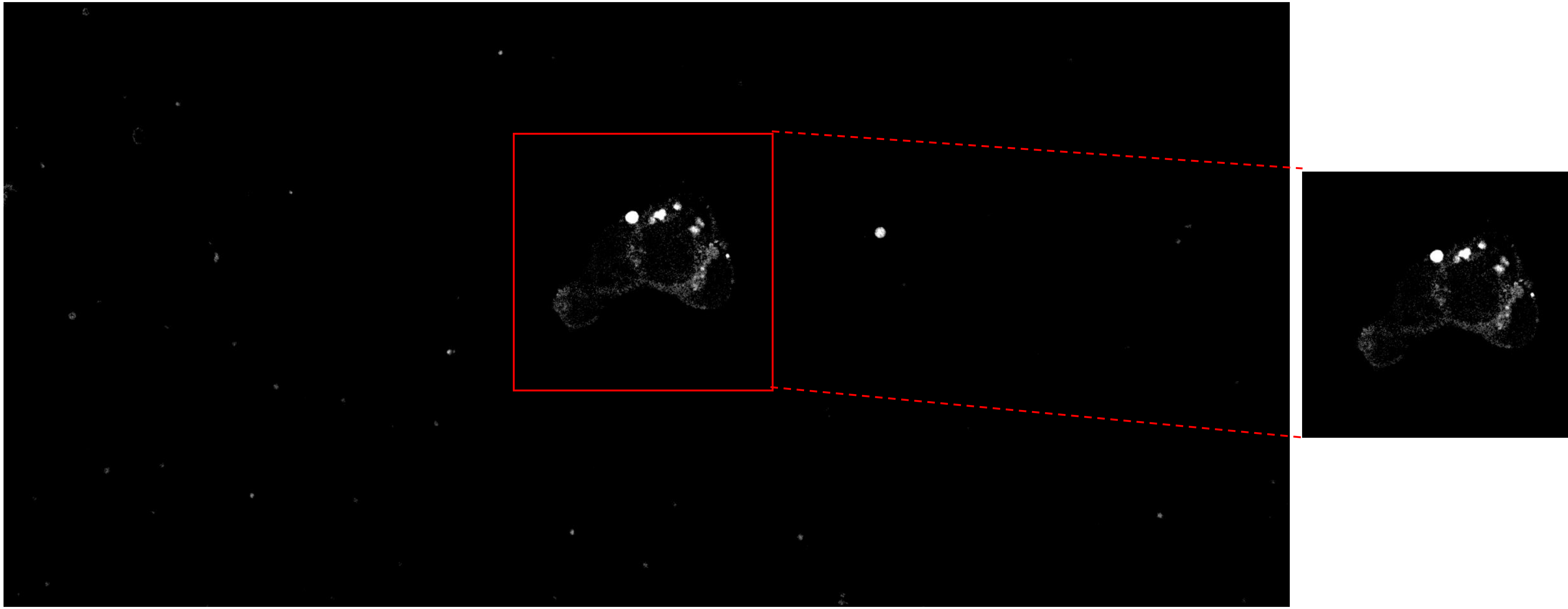

Figure 3-B
WT-DAPI

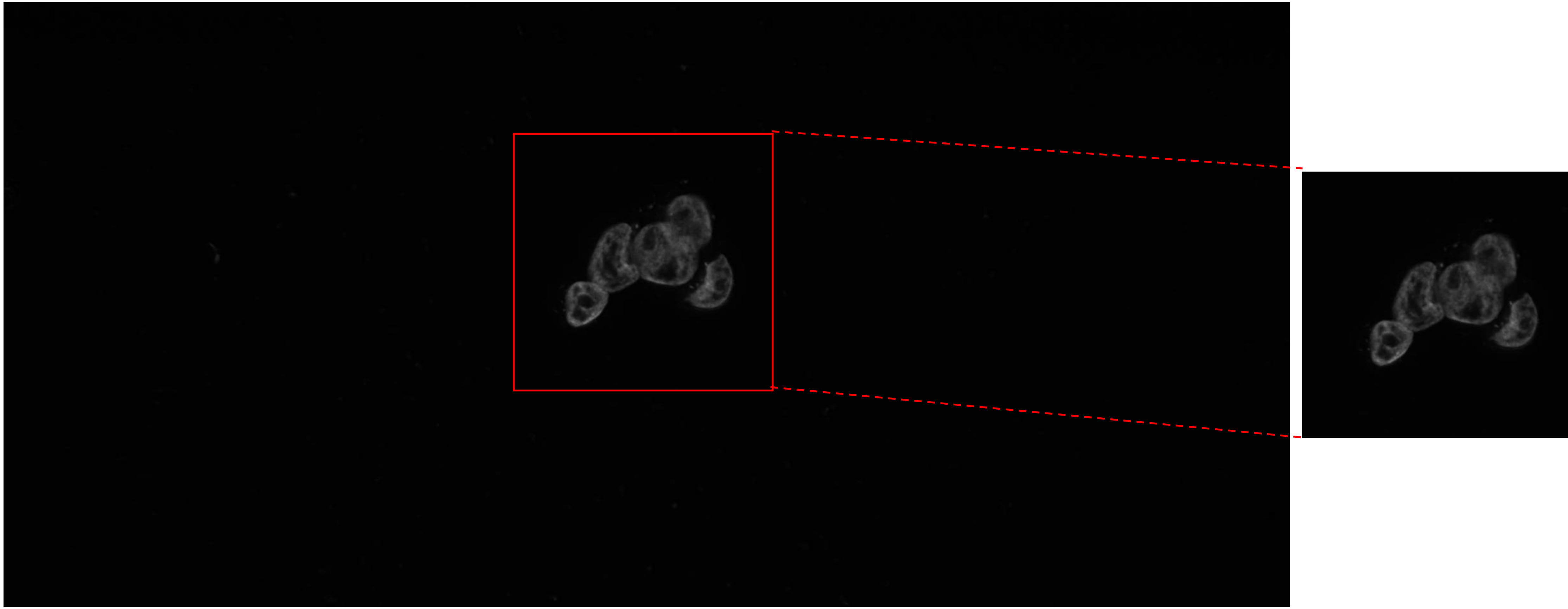

Figure 3-B
MT-Merge

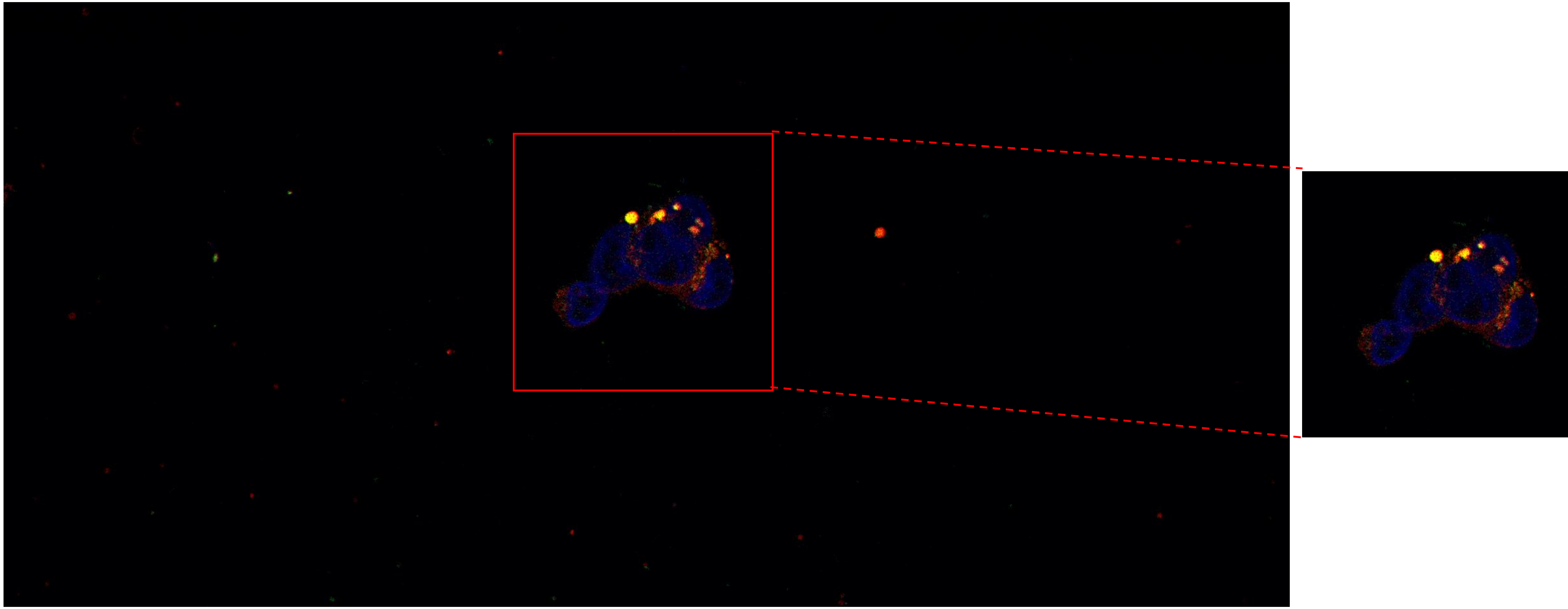

Figure 3-C
Ctrl-GFP

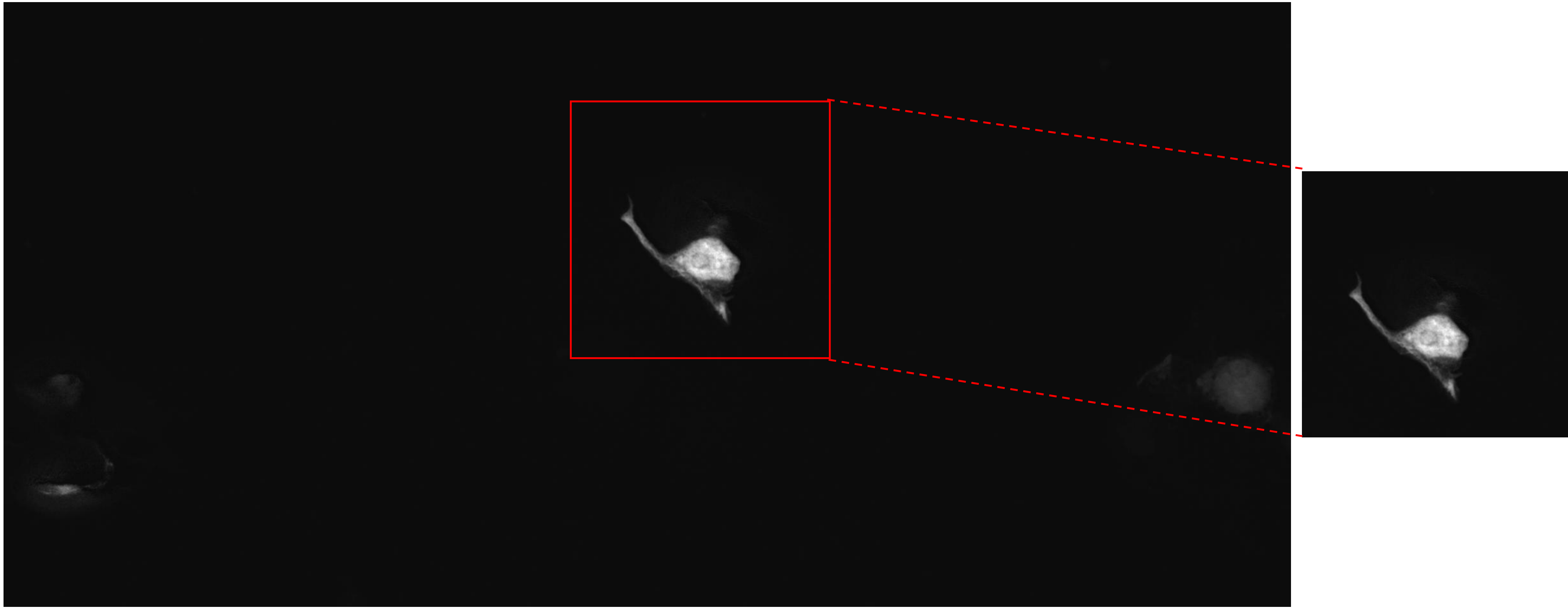

Figure 3-C
Ctrl-LC3

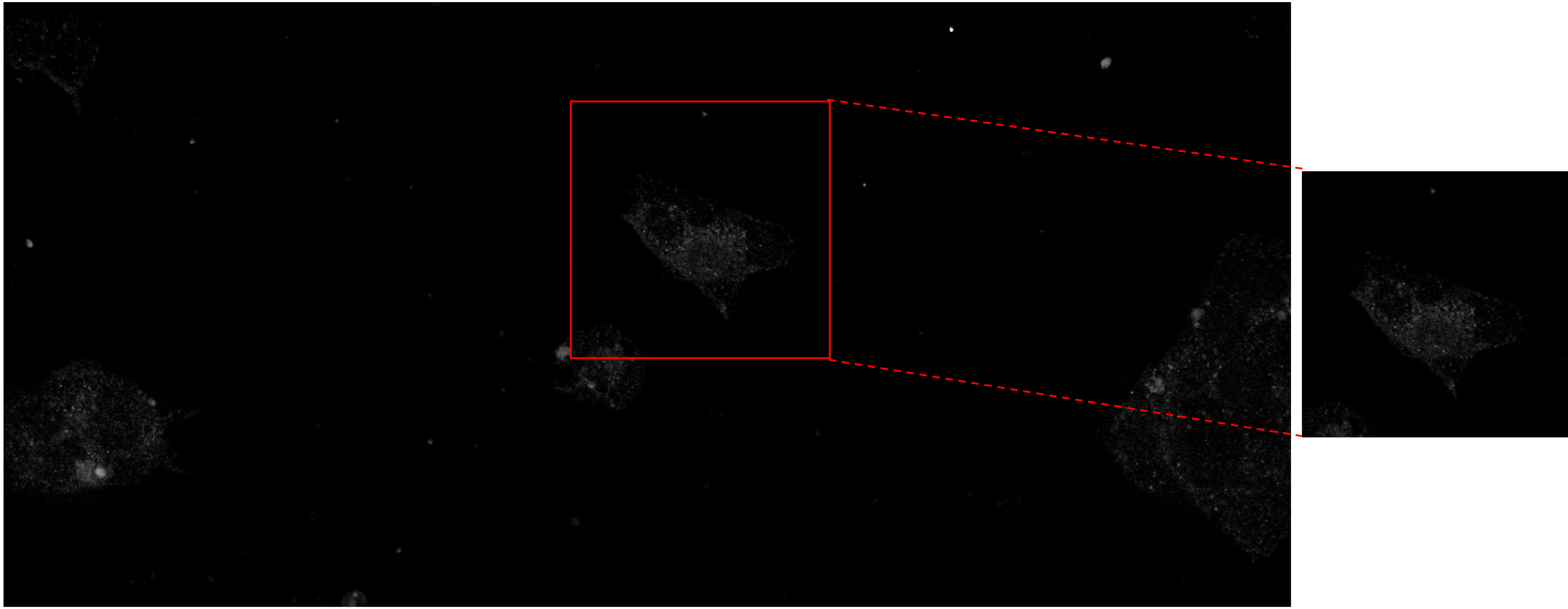

Figure 3-C
Ctrl-DAPI

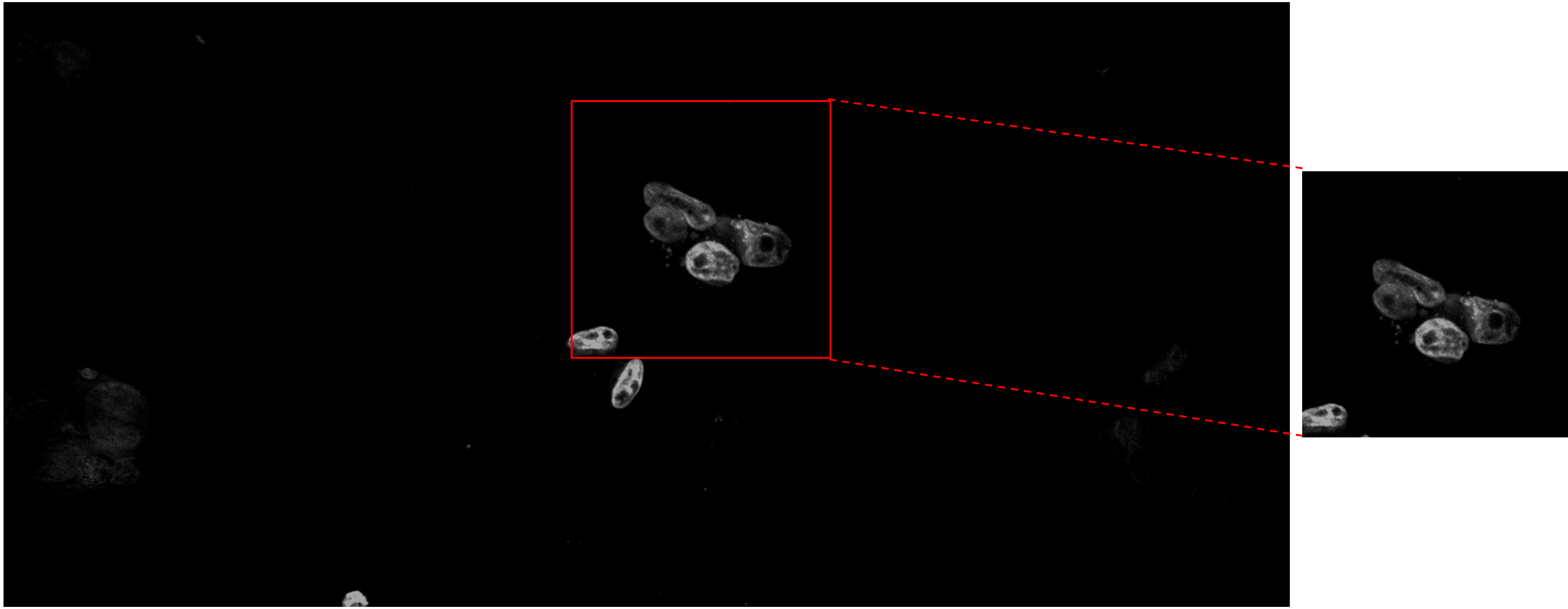

Figure 3-C
Ctrl-Merge

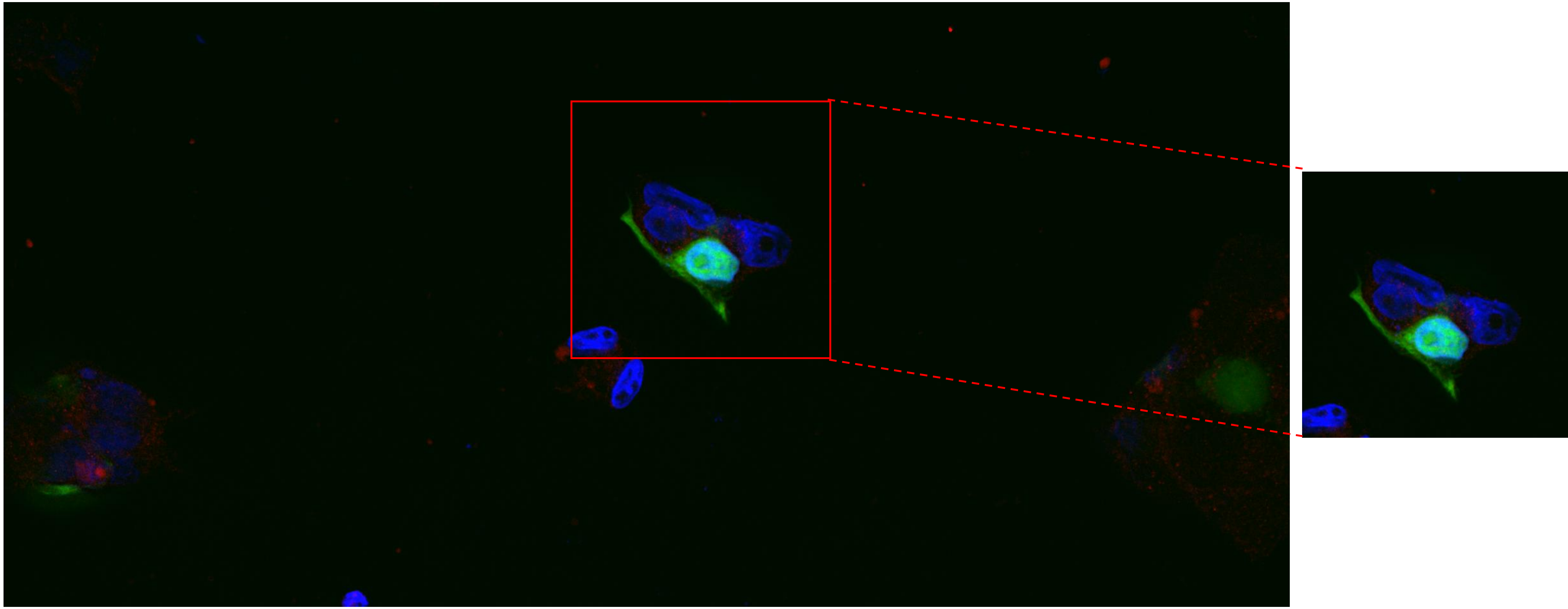

Figure 3-C
WT-GFP

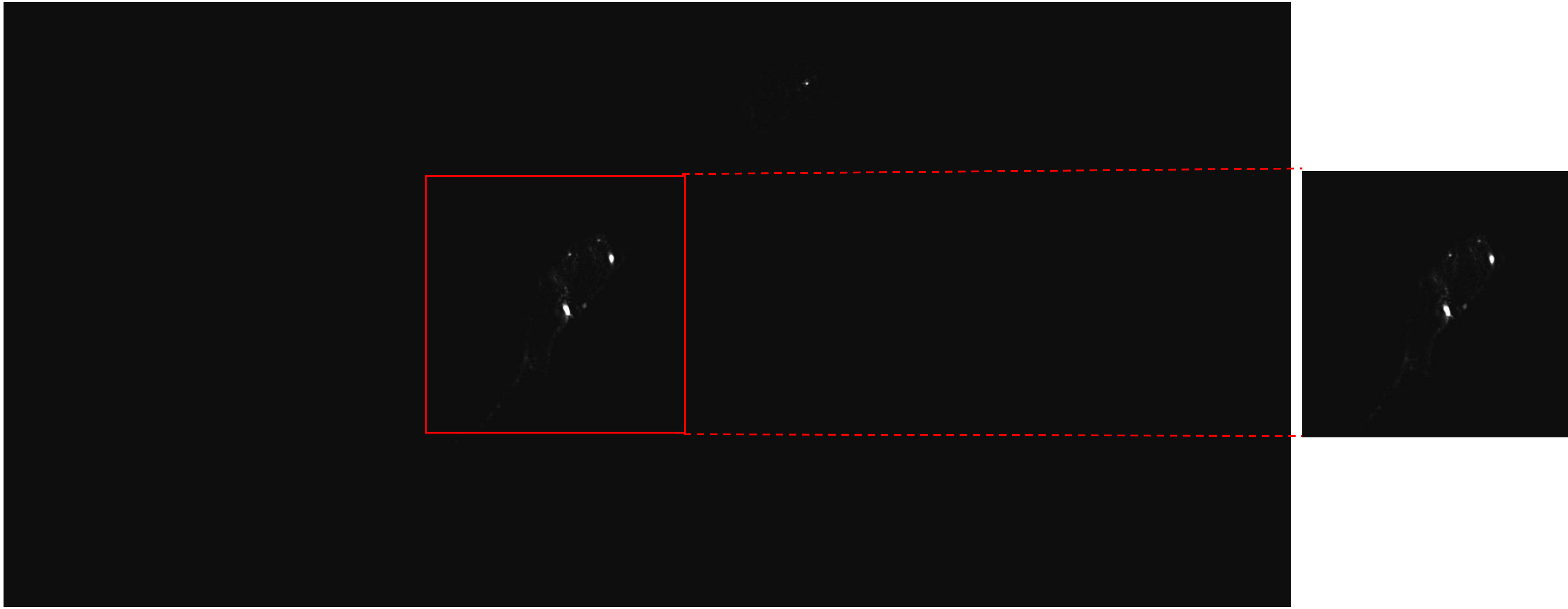

Figure 3-C
WT-LC3

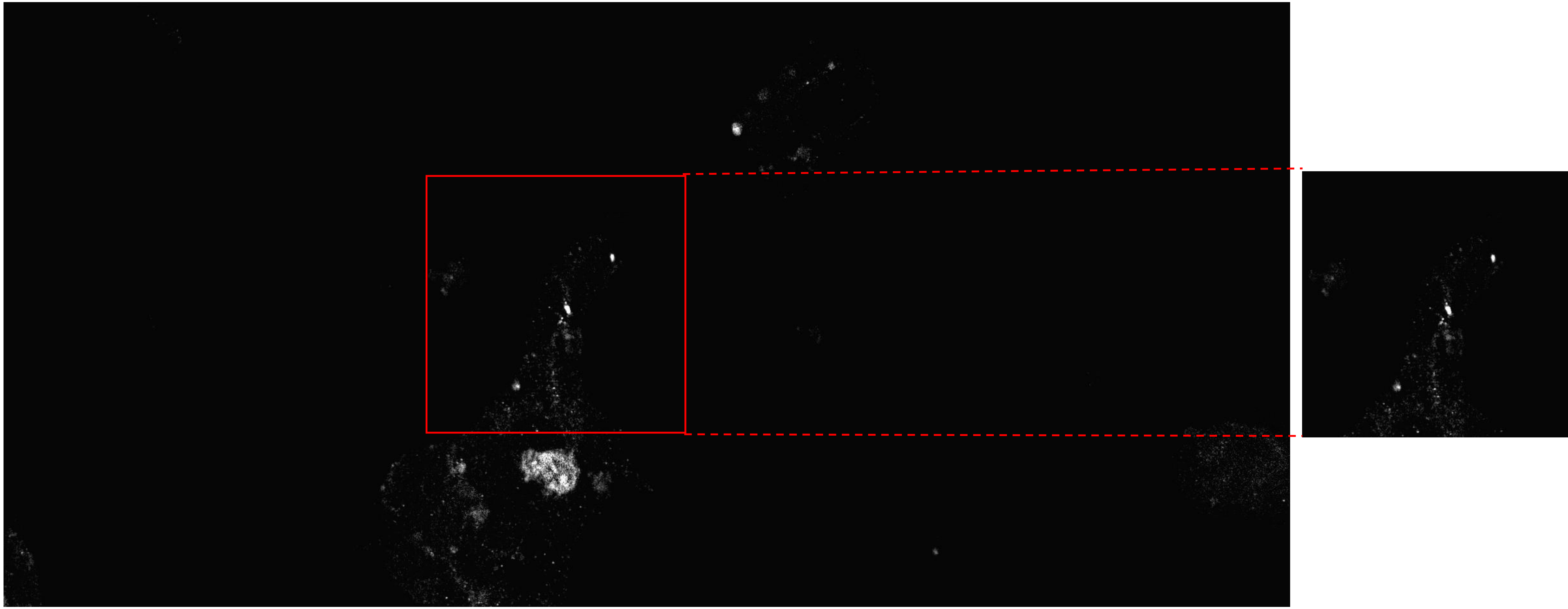

Figure 3-C
WT-DAPI

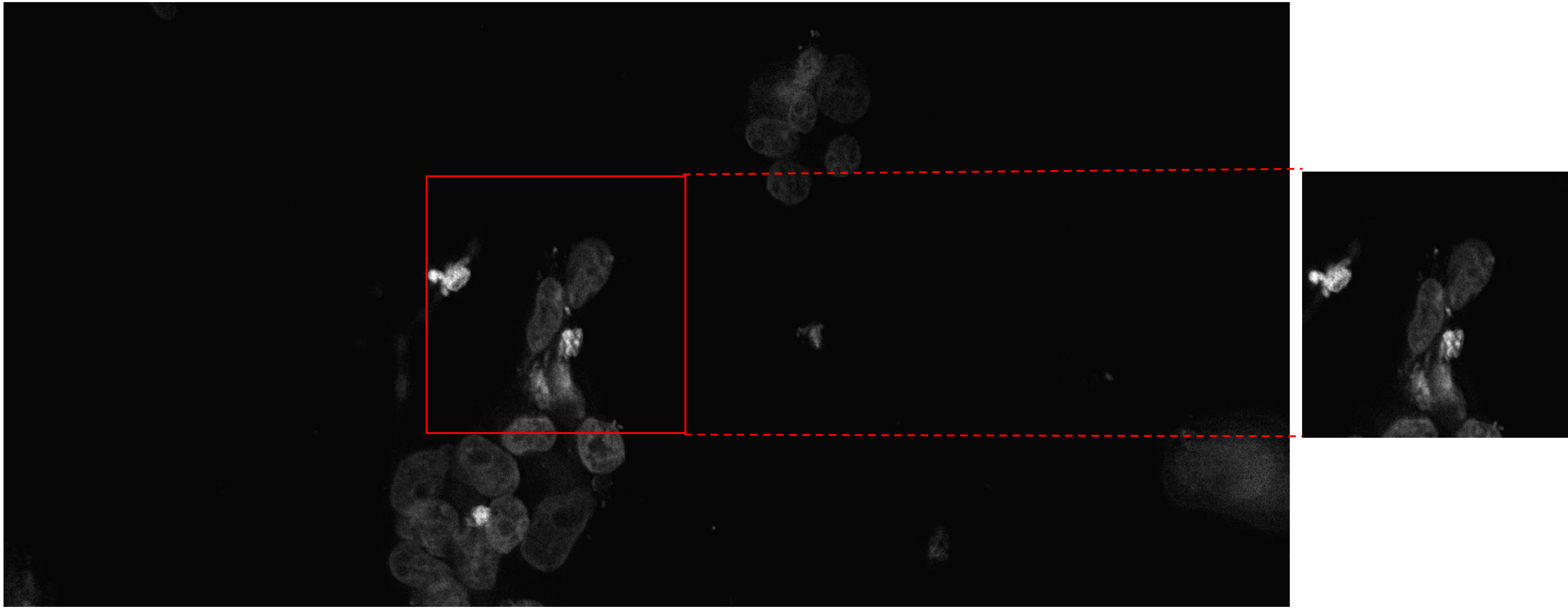

Figure 3-C
WT-Merge

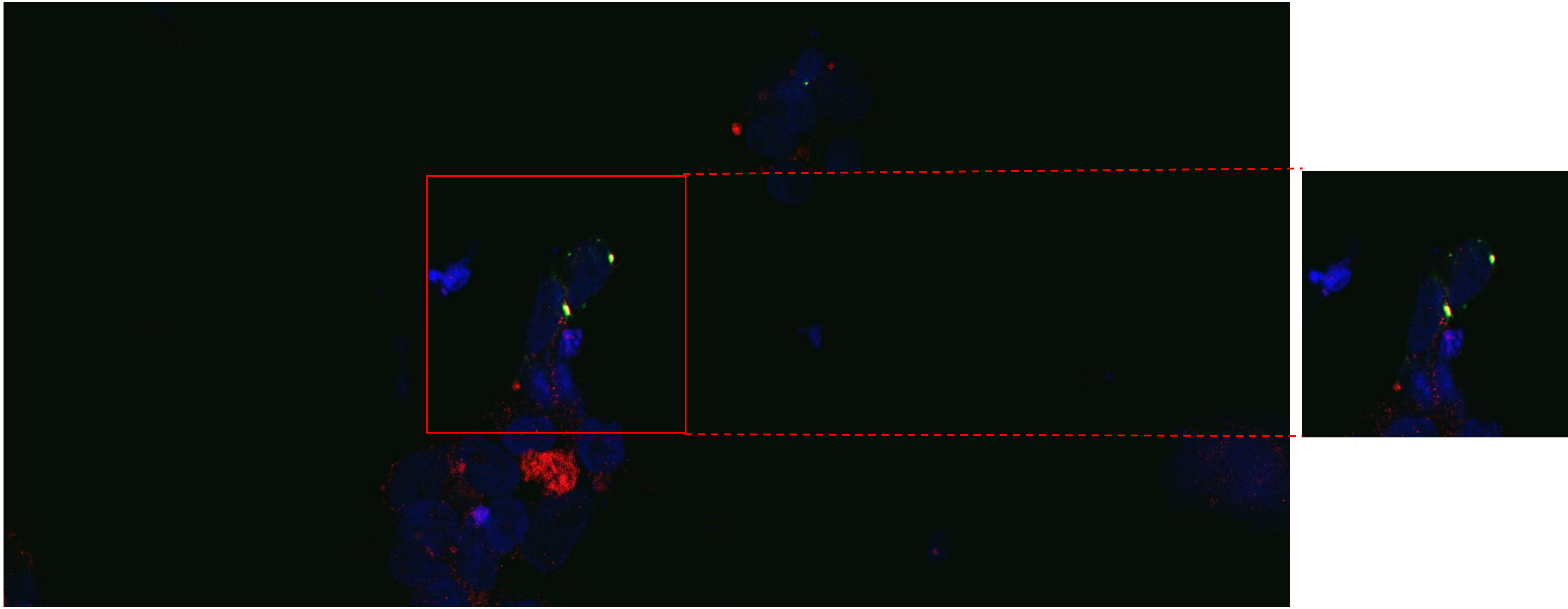

Figure 3-C
MT-GFP

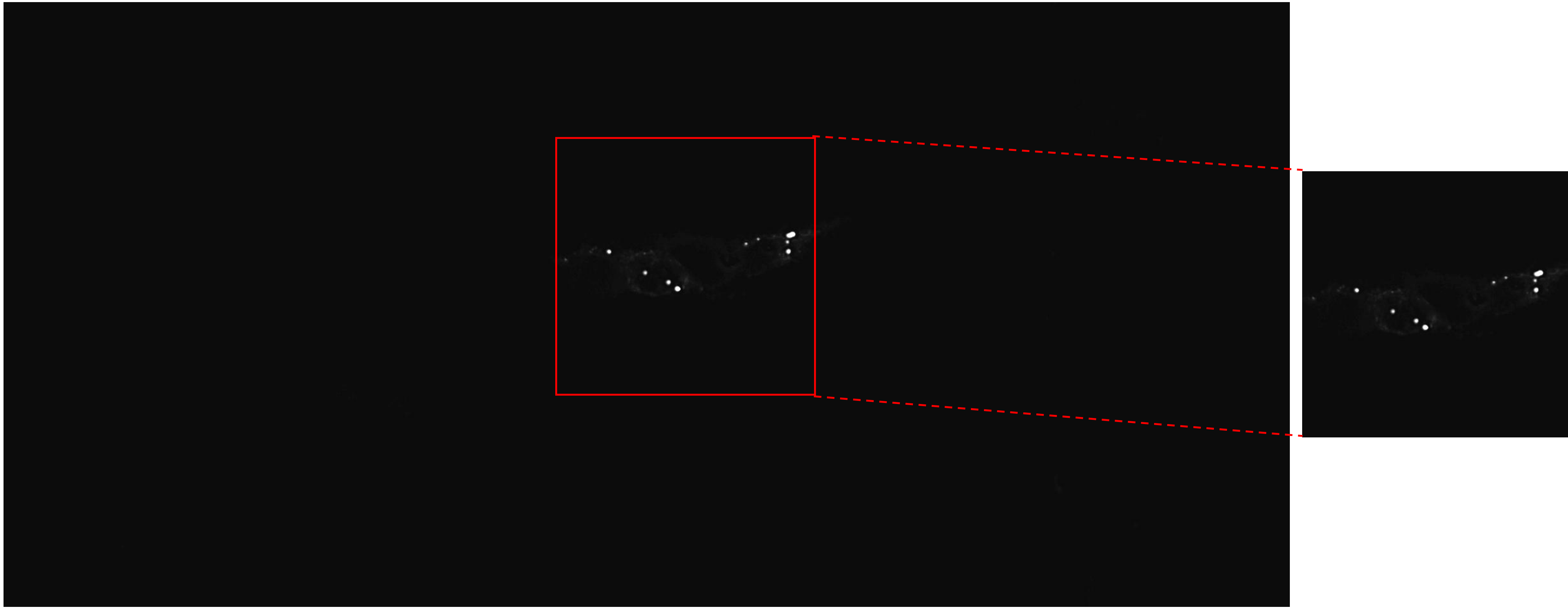

Figure 3-C
MT-LC3

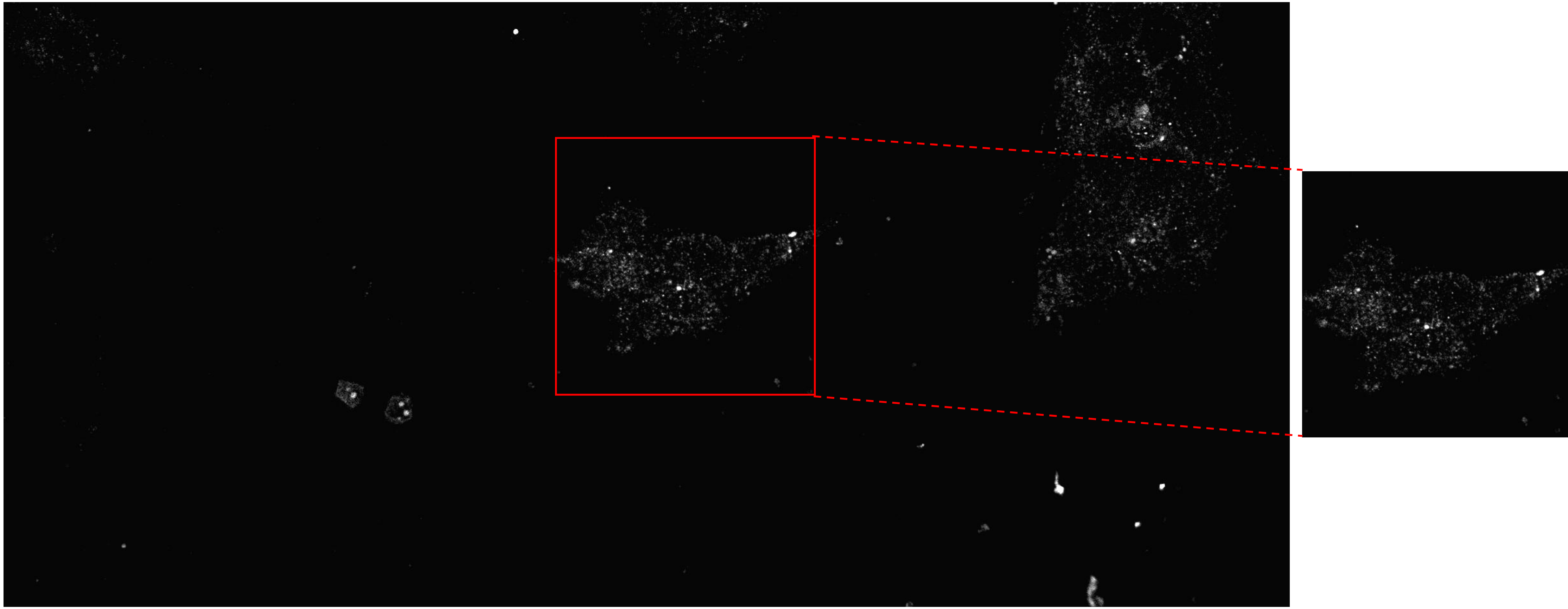

Figure 3-C
MT-DAPI

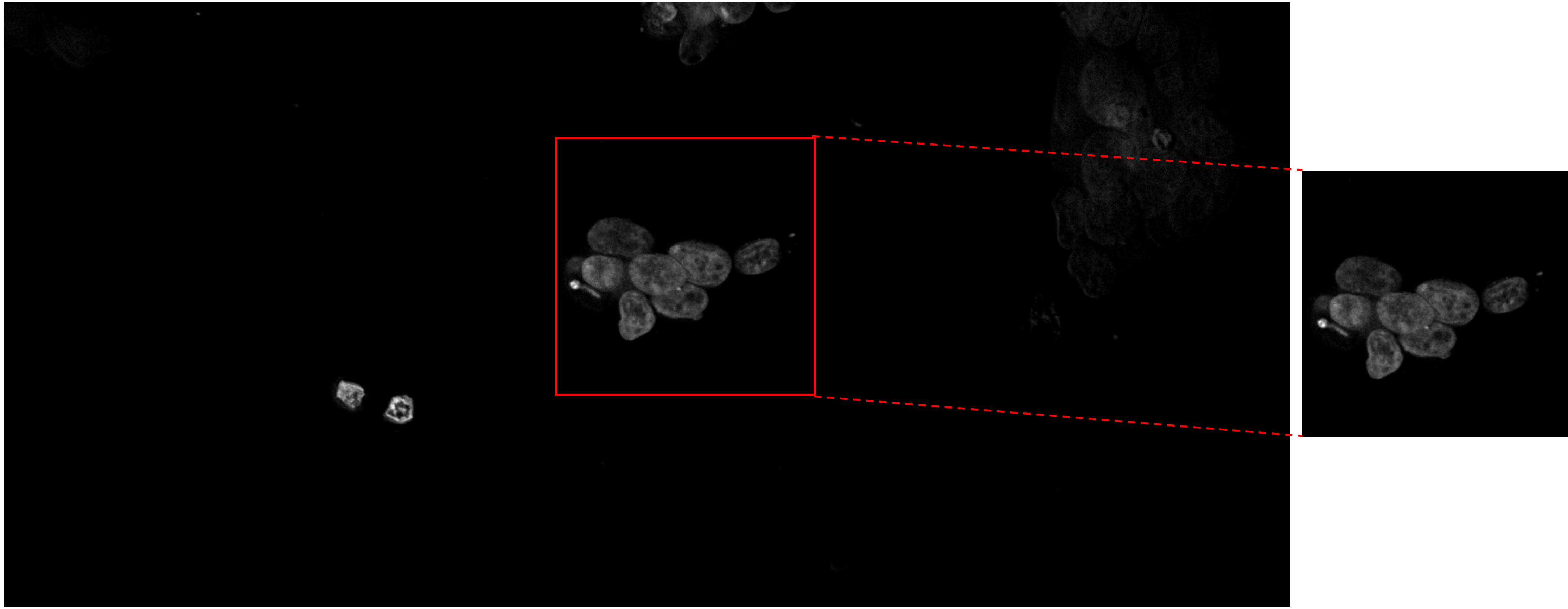

Figure 3-C
MT-Merge

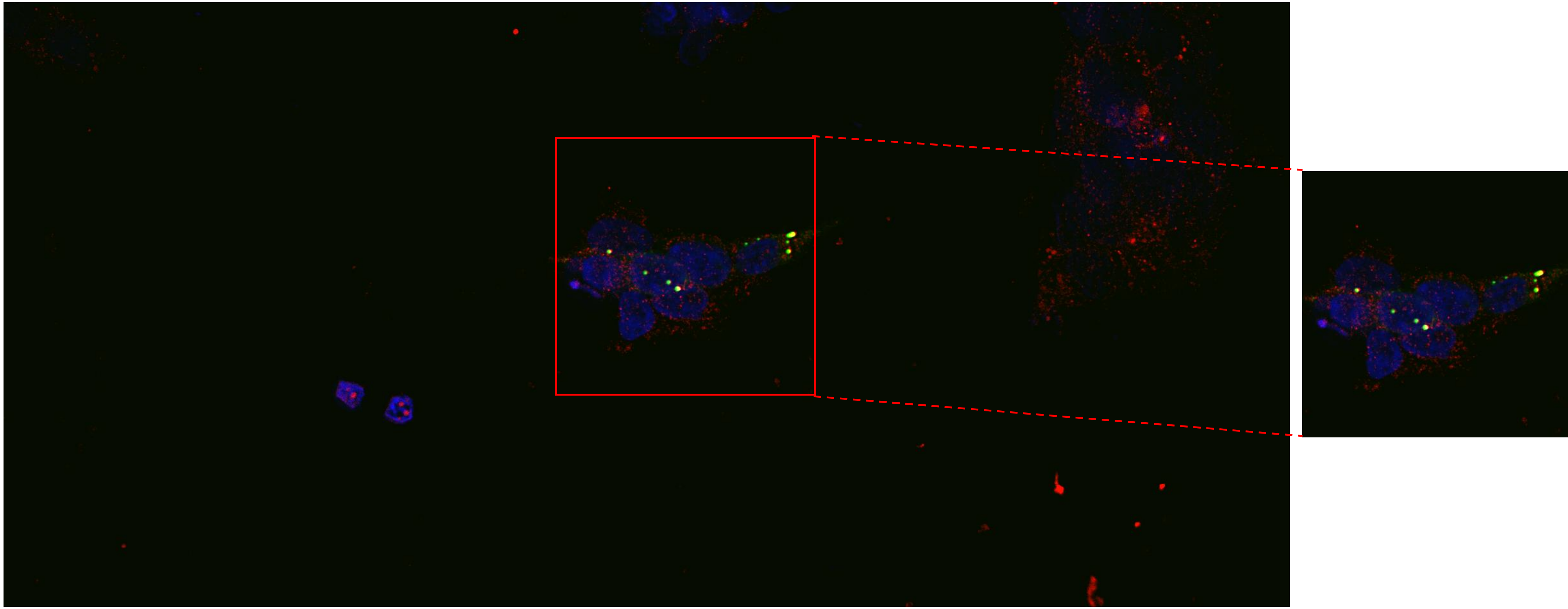

Figure 3-D
Ctrl-GFP

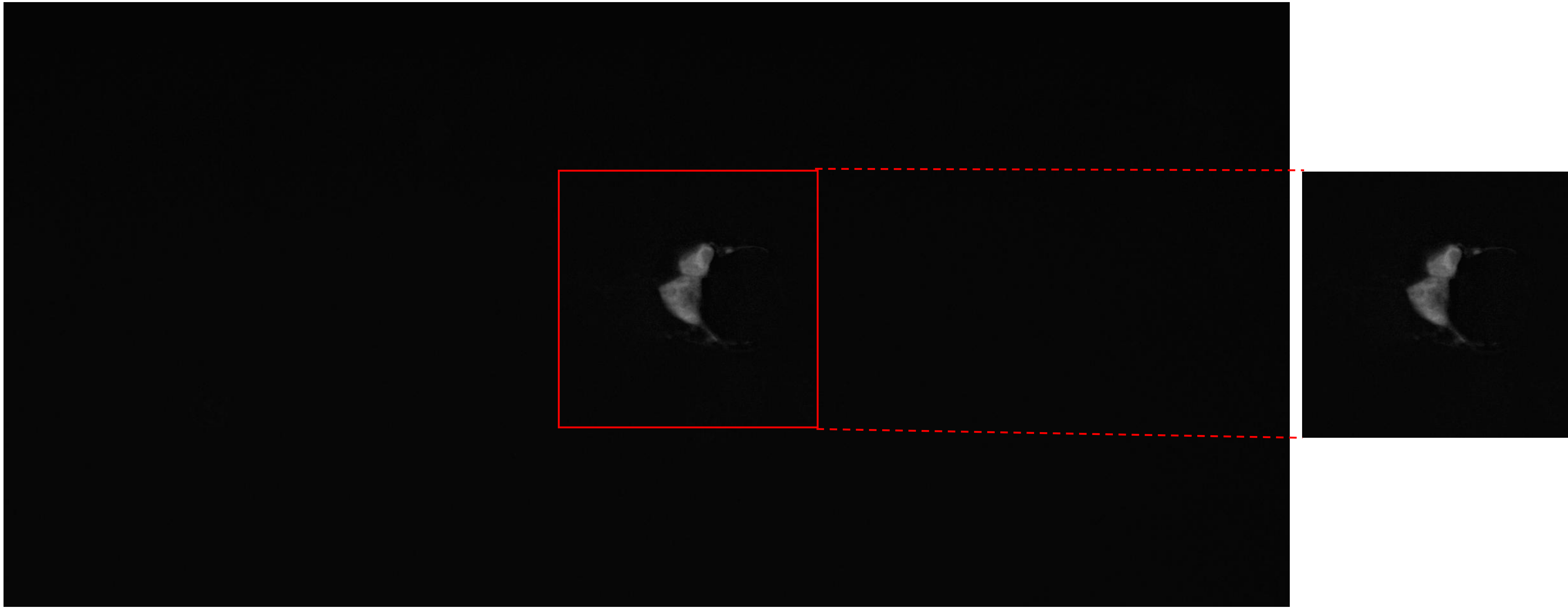

Figure 3-D
Ctrl-LAMP2

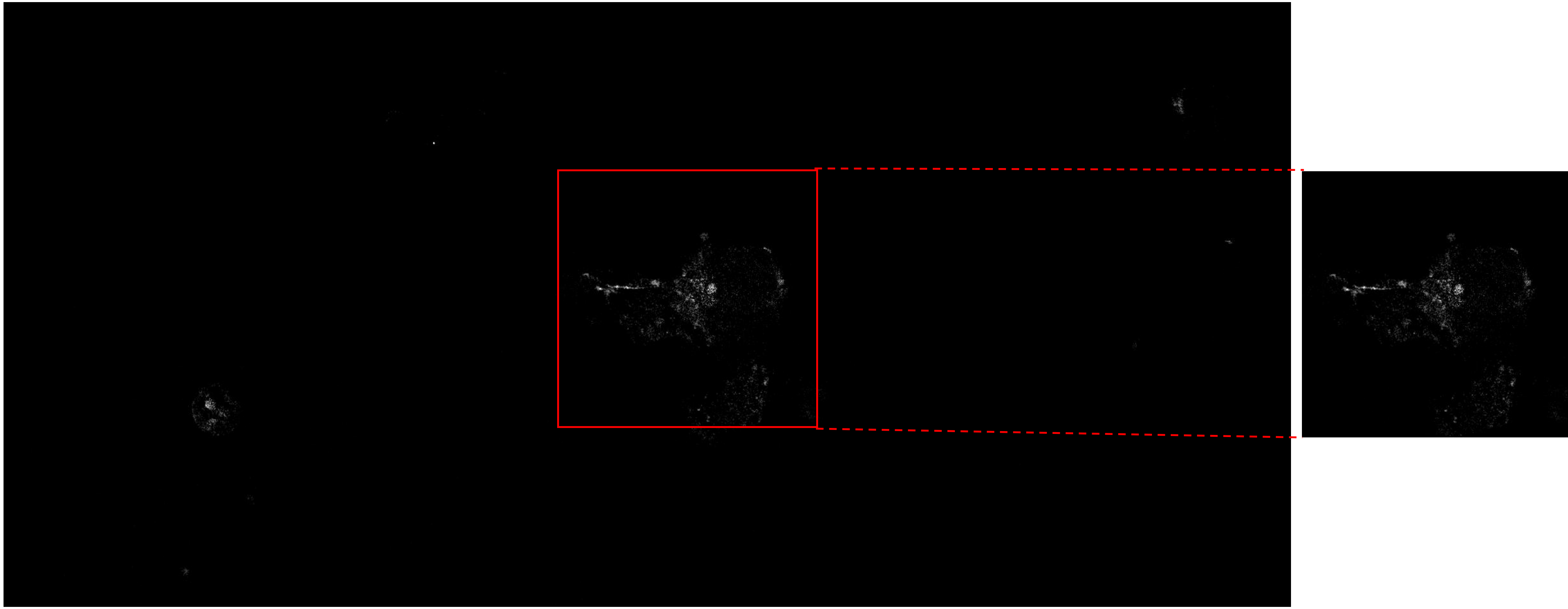

Figure 3-D
Ctrl-DAPI

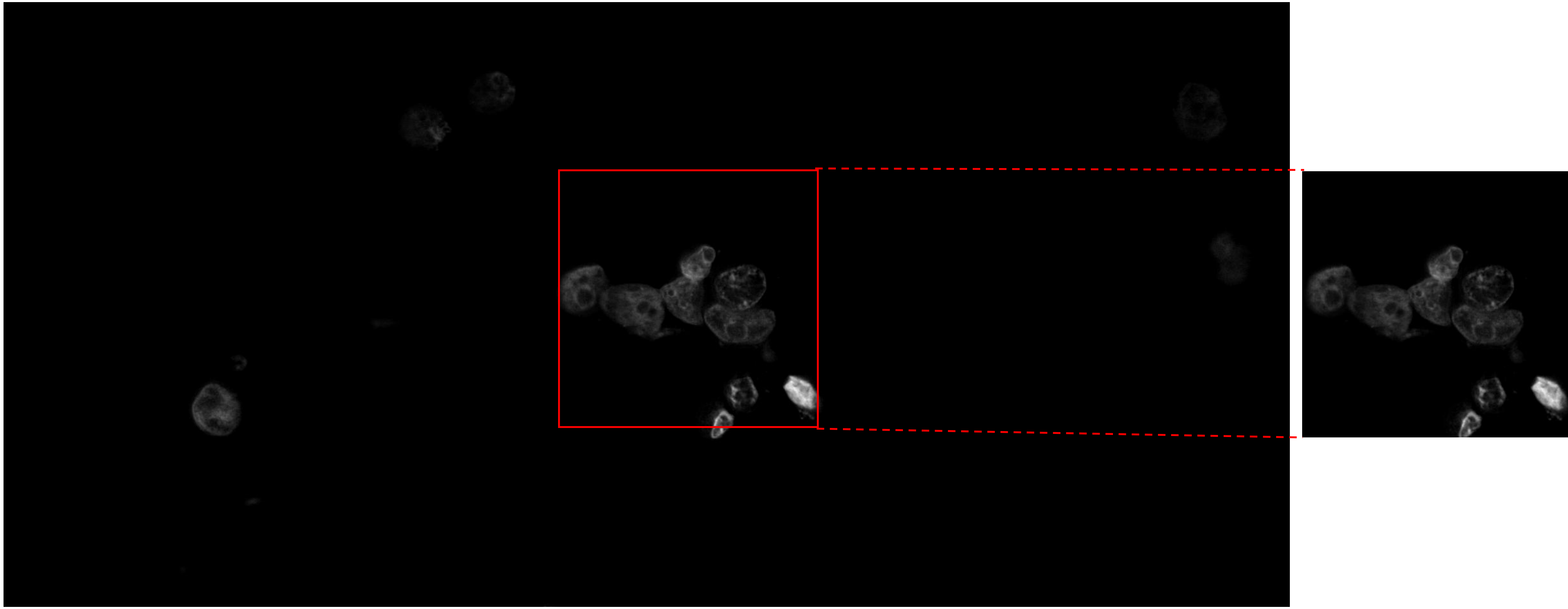

Figure 3-D
Ctrl-Merge

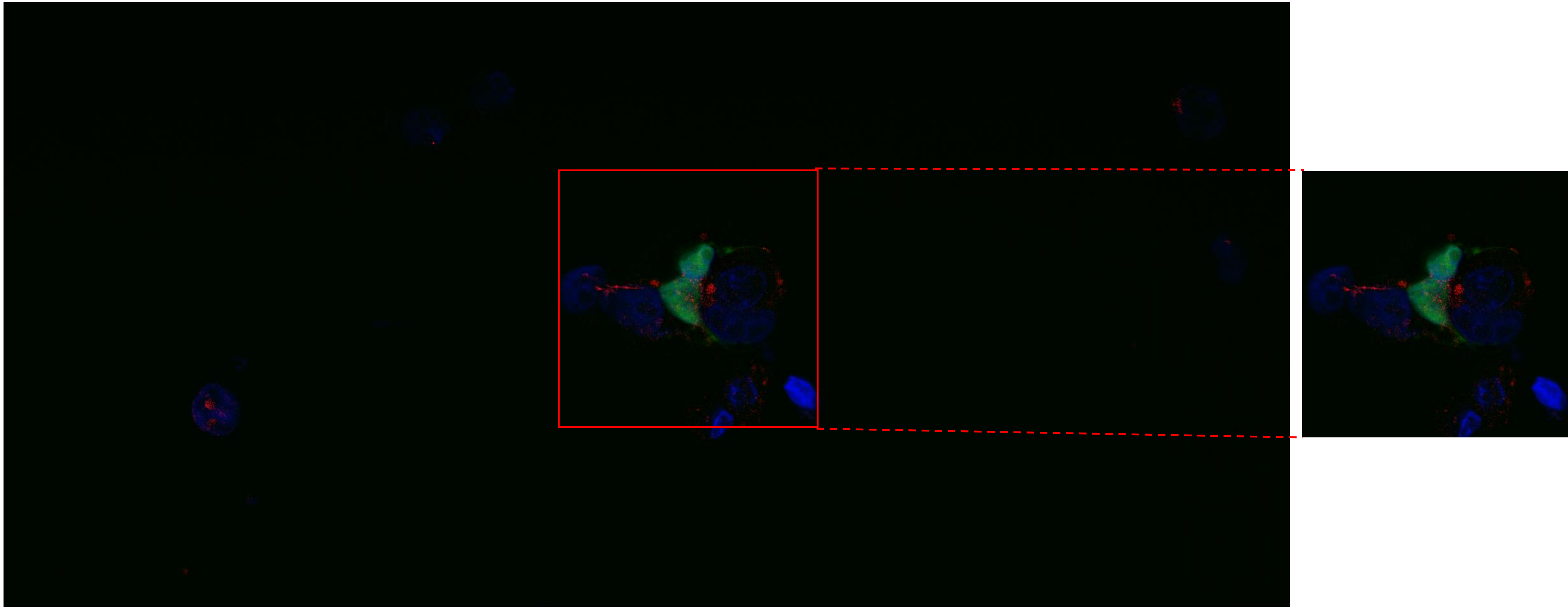

Figure 3-D
WT-GFP

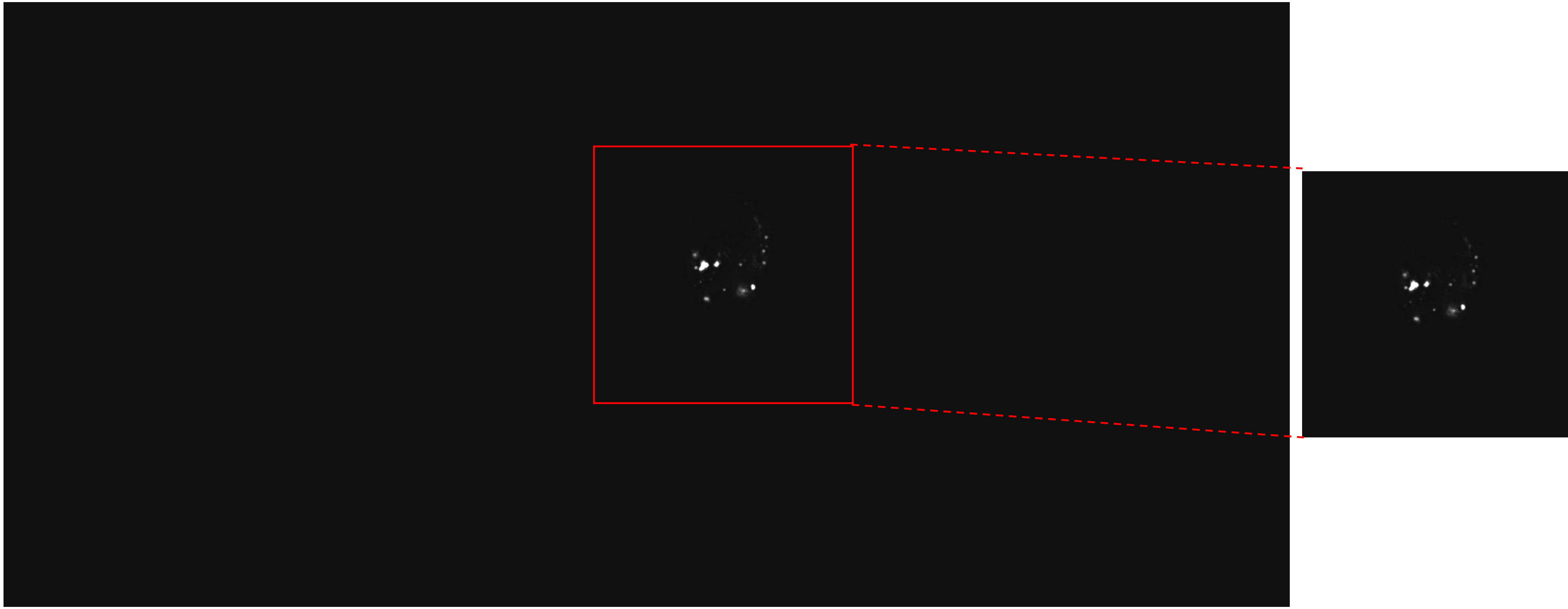

Figure 3-D
WT-LAMP2

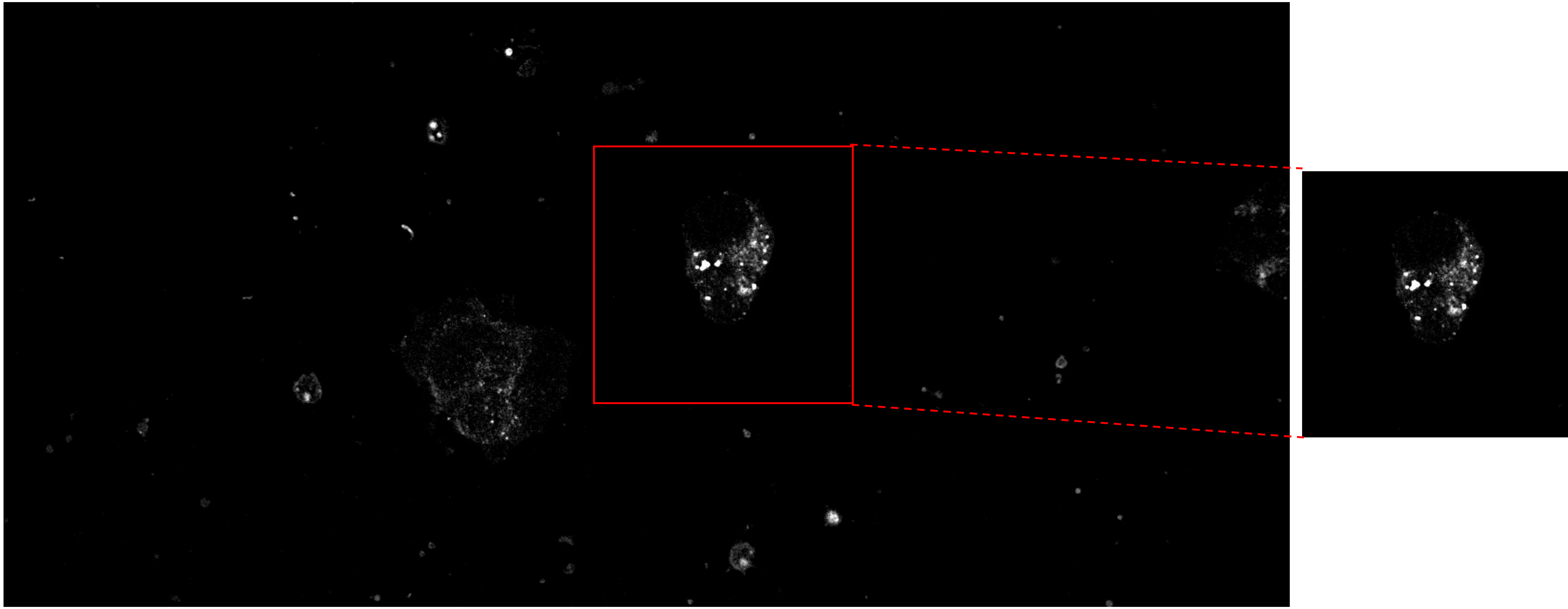

Figure 3-D
WT-DAPI

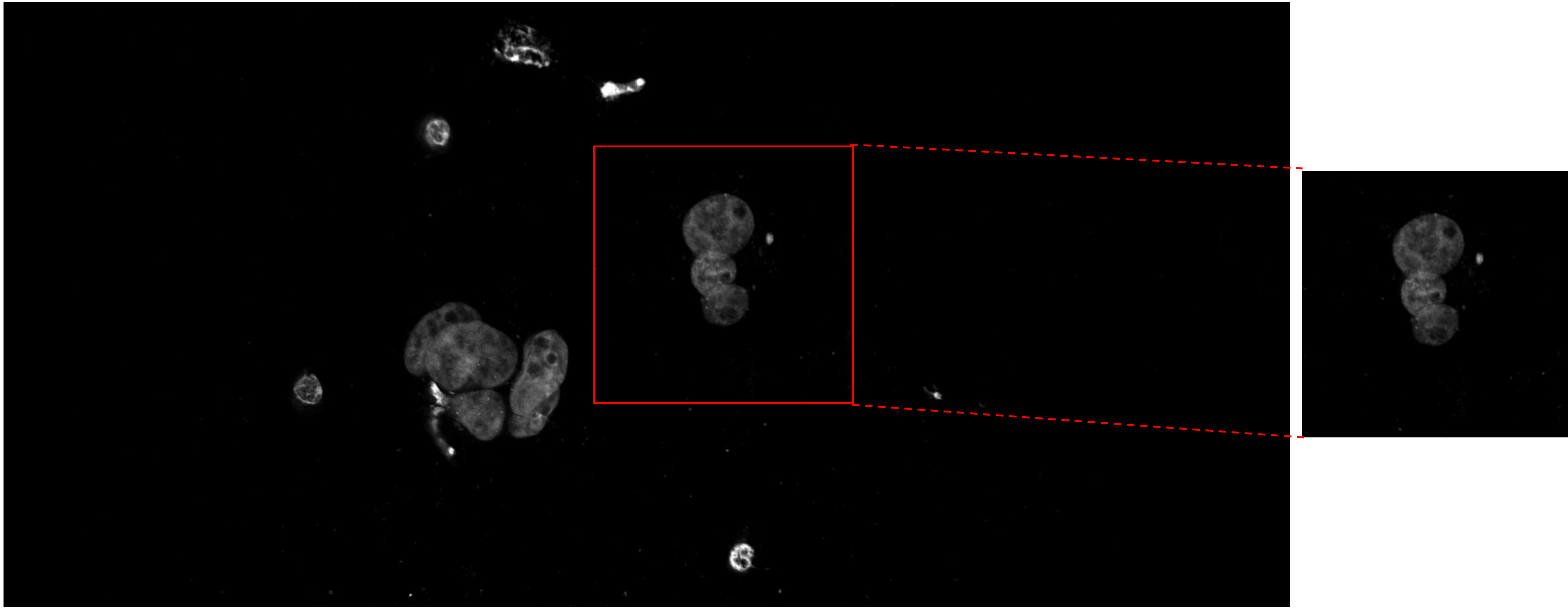

Figure 3-D
WT-Merge

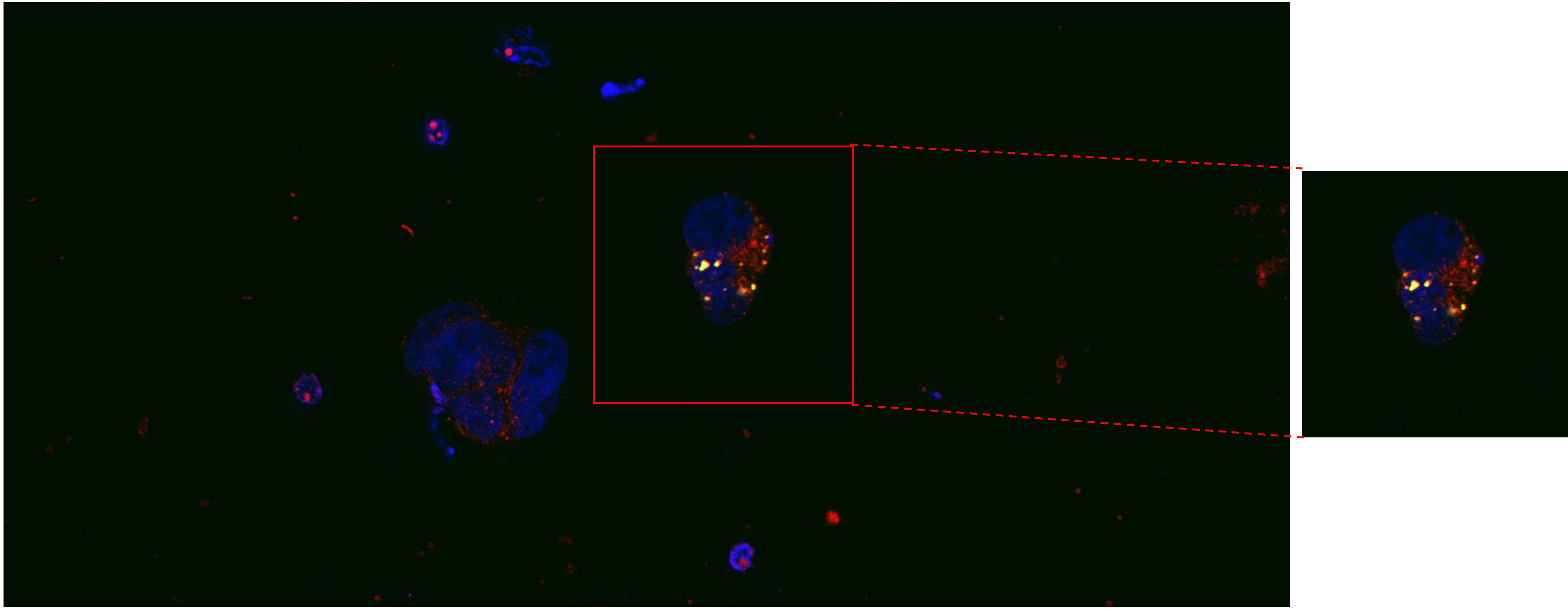

Figure 3-D
MT-GFP

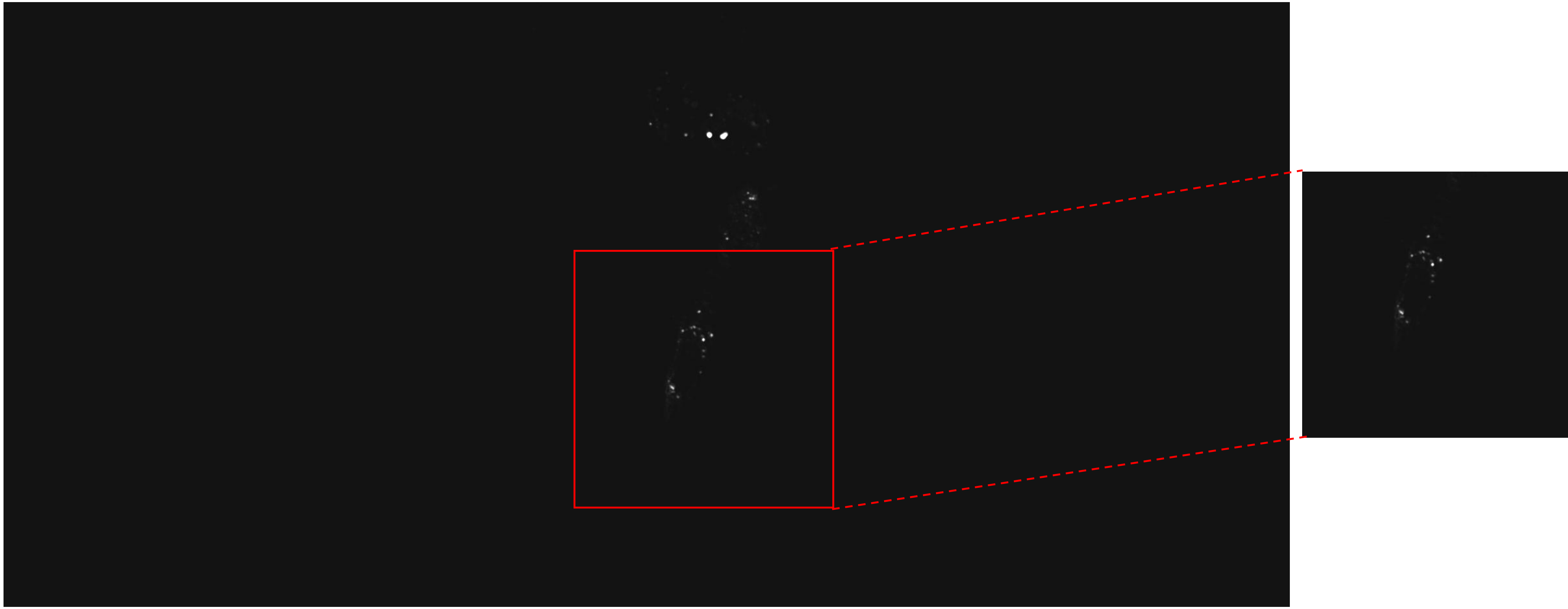

Figure 3-D
MT-LAMP2

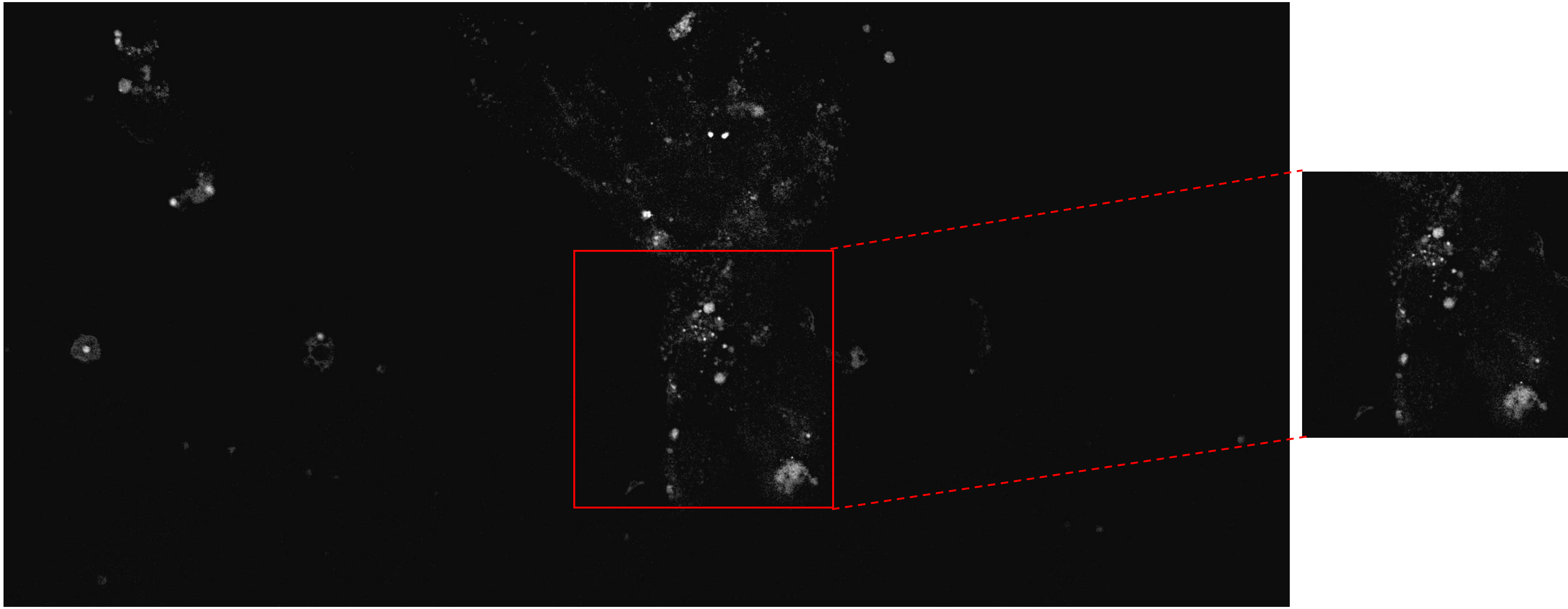

Figure 3-D
MT-DAPI

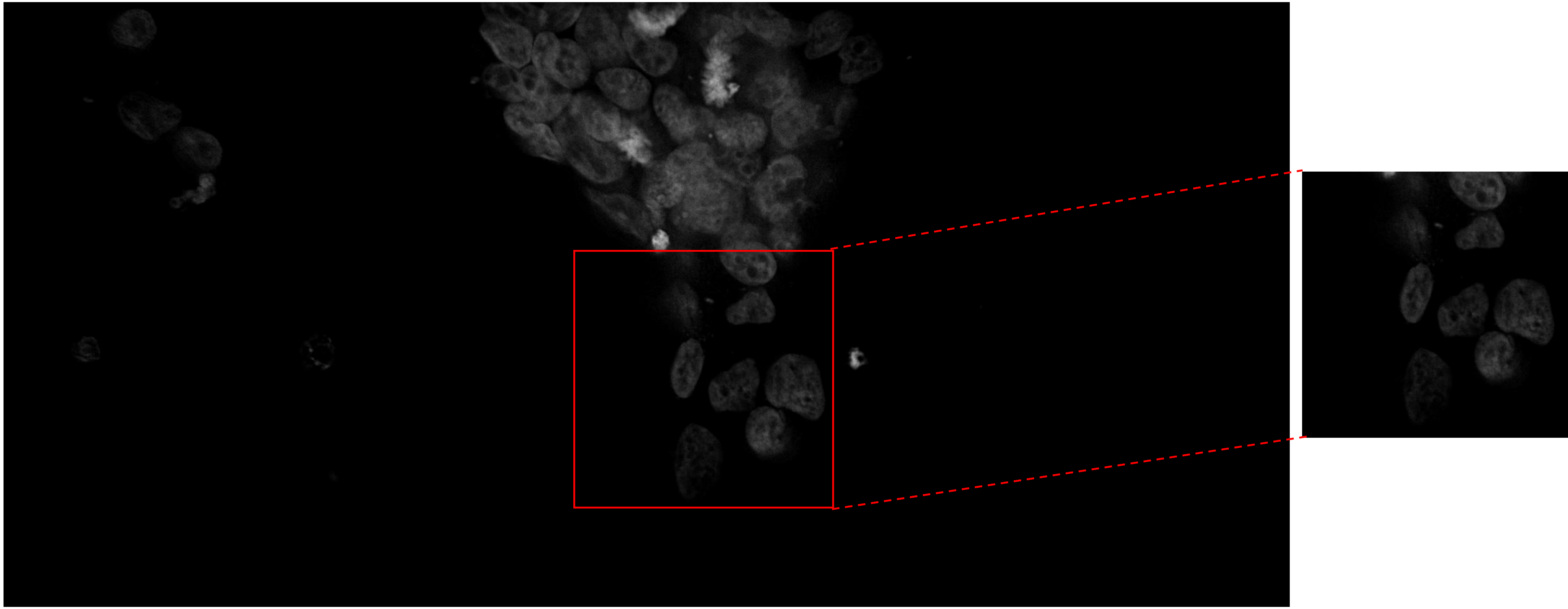

Figure 3-D
MT-Merge

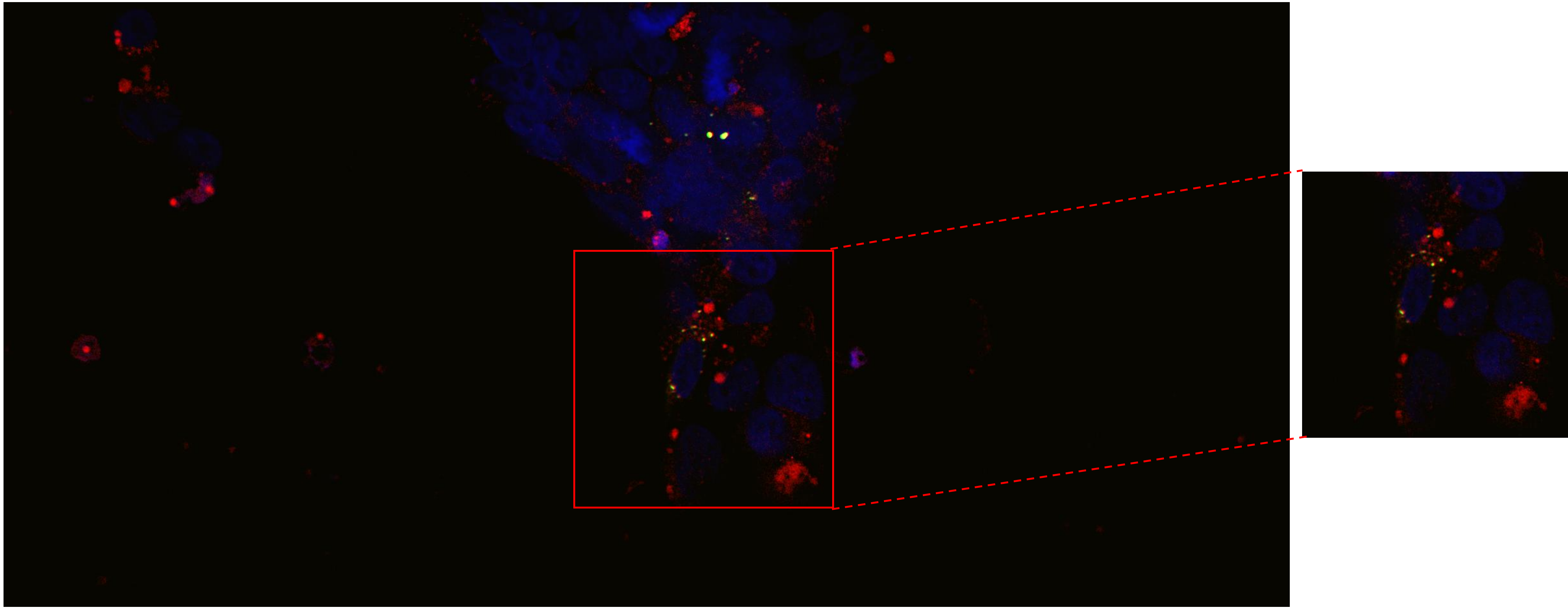

Figure 3-E
Ctrl-GFP

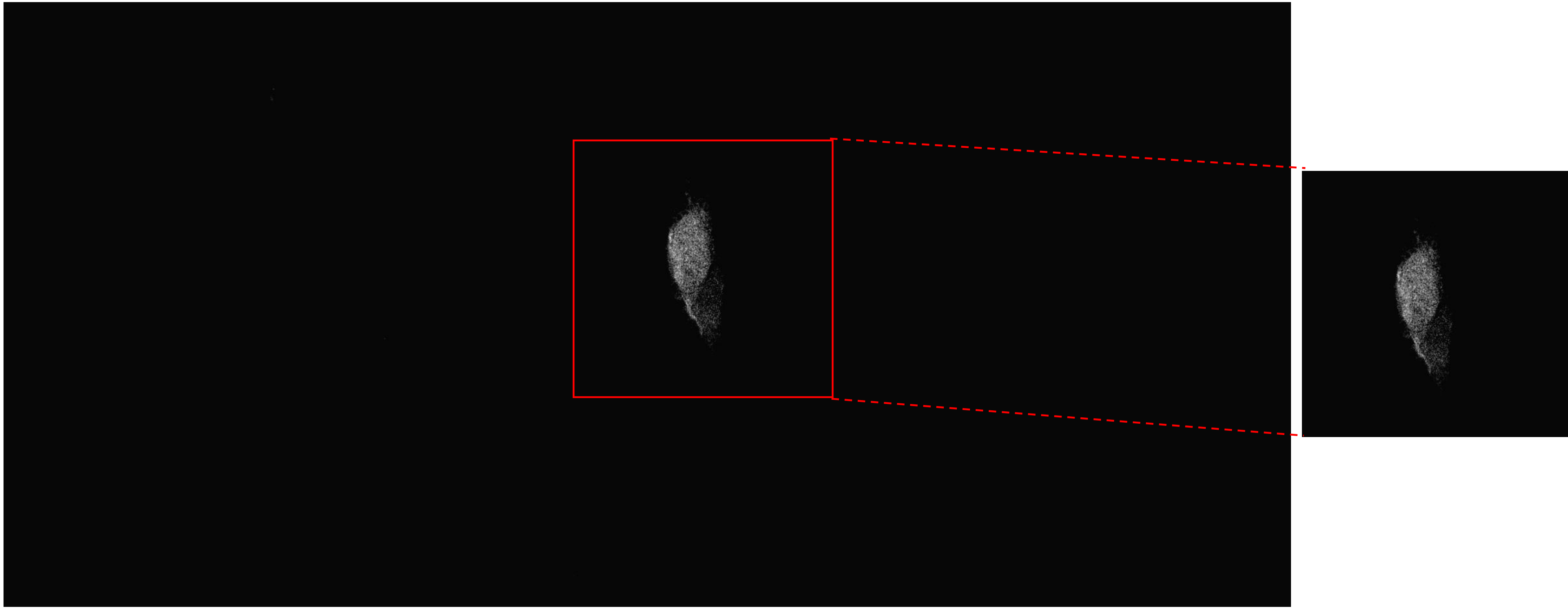

Figure 3-E
Ctrl-TOMM20

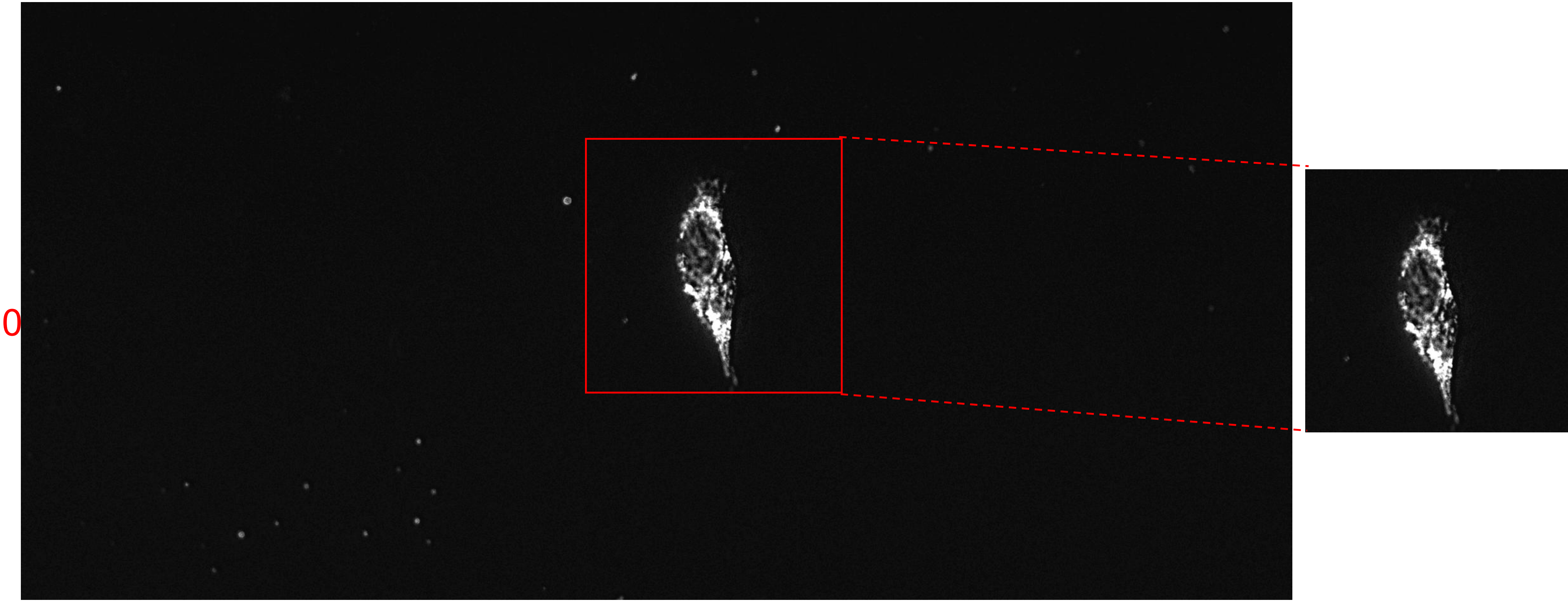

Figure 3-E
Ctrl-DAPI

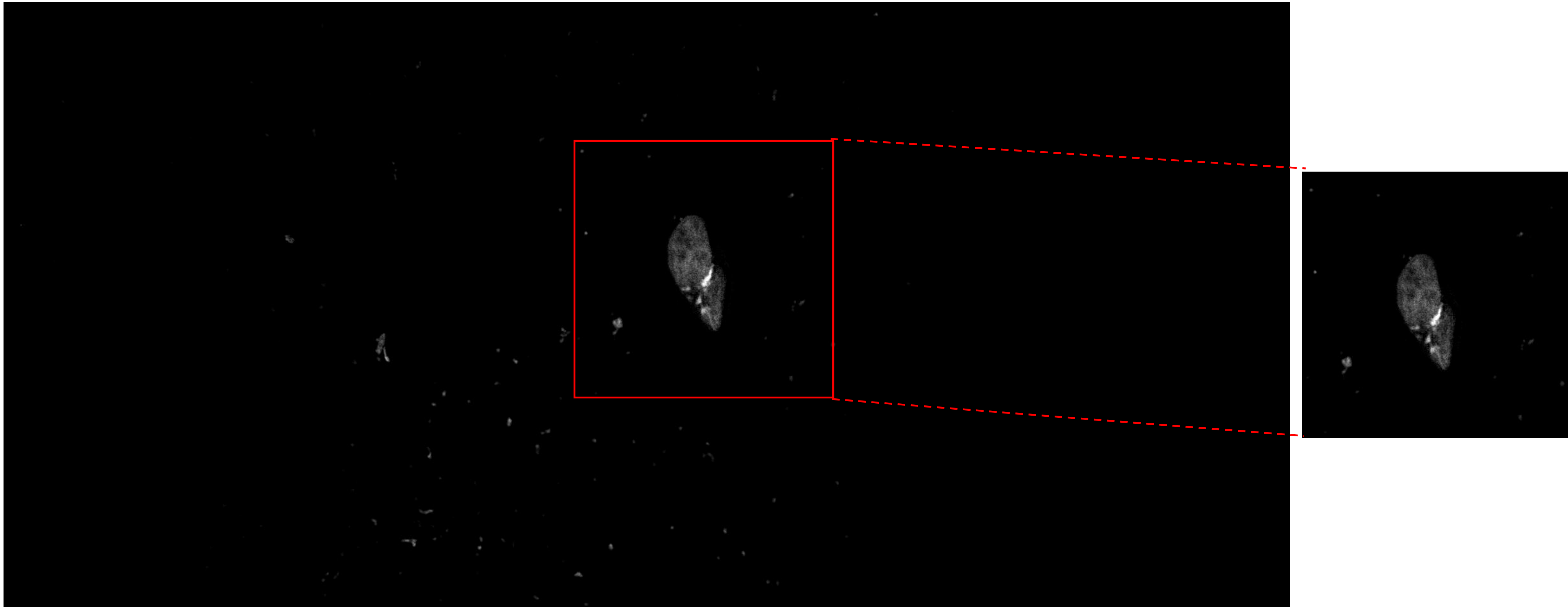

Figure 3-E
Ctrl-Merge

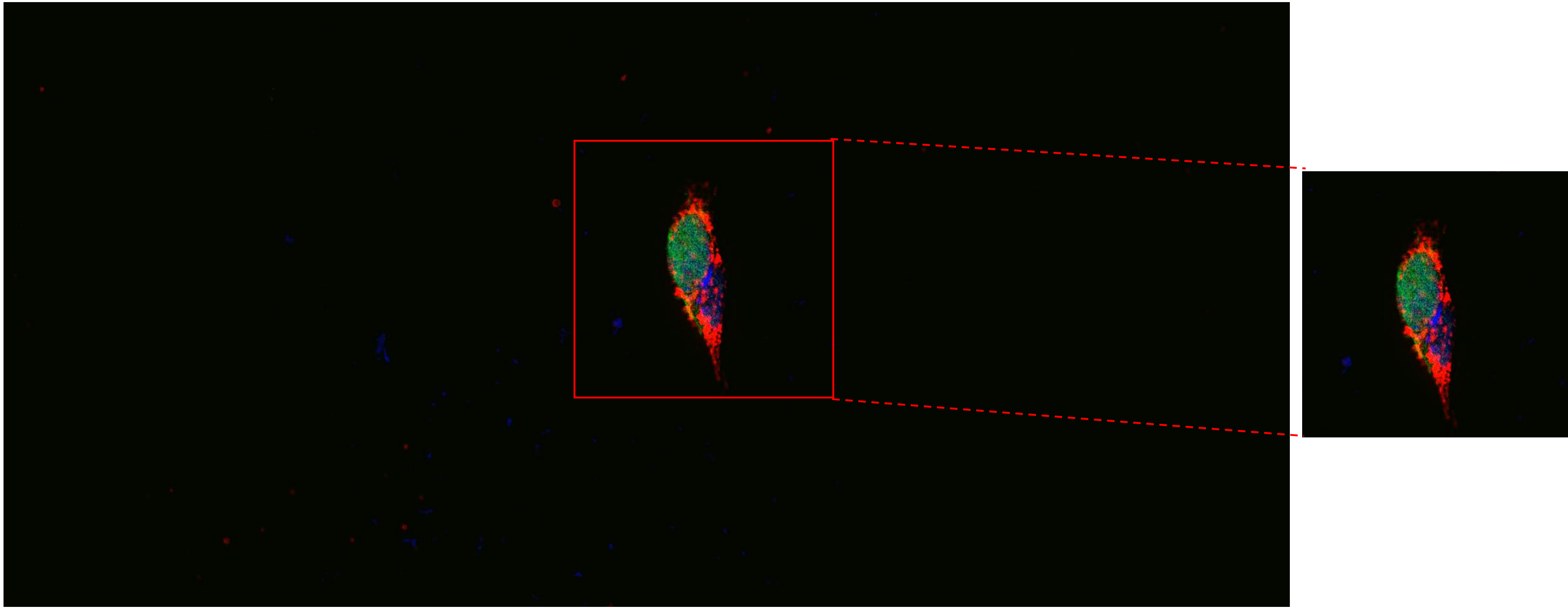

Figure 3-E
WT-GFP

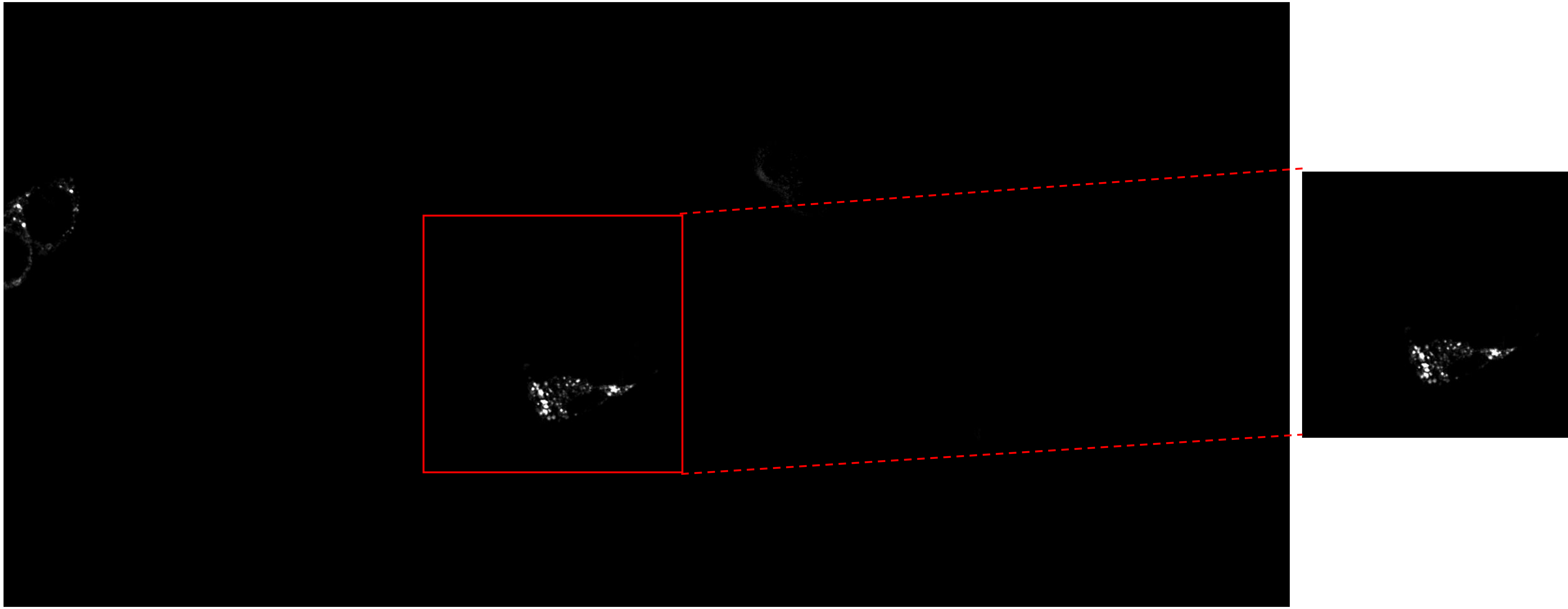

Figure 3-E
WT-TOMM20

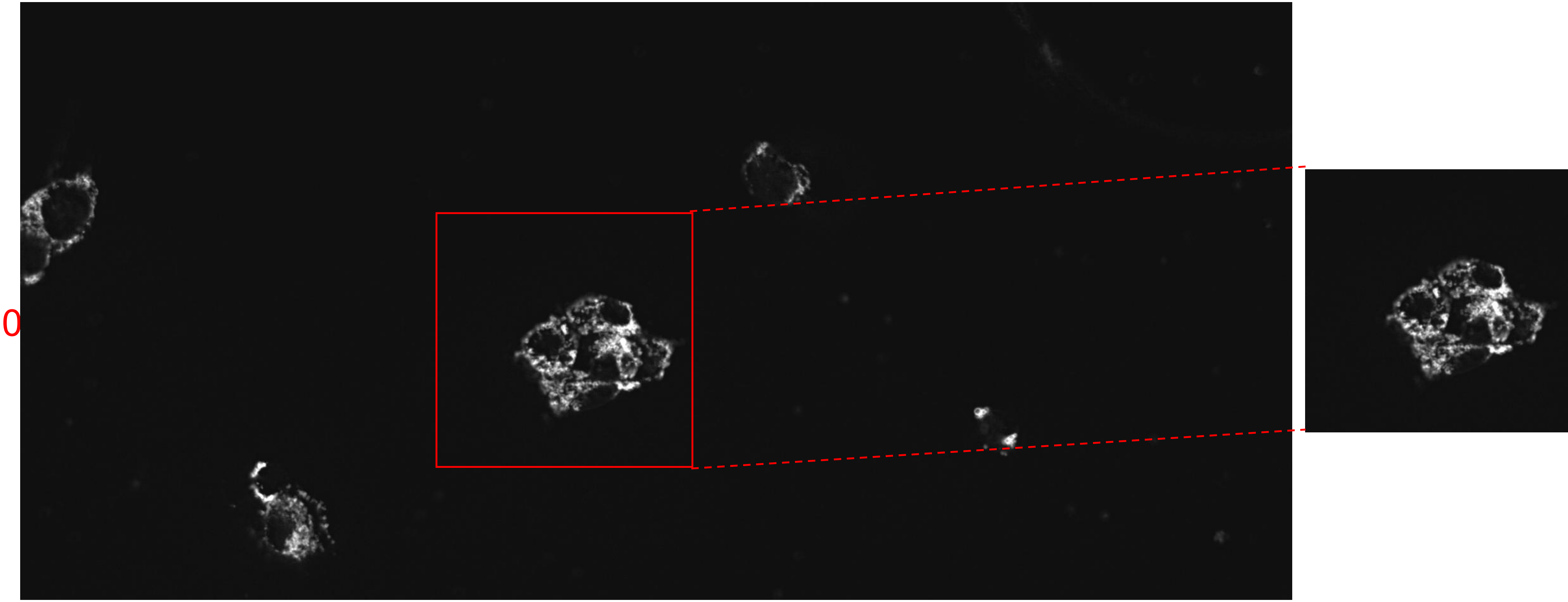

Figure 3-E
WT-DAPI

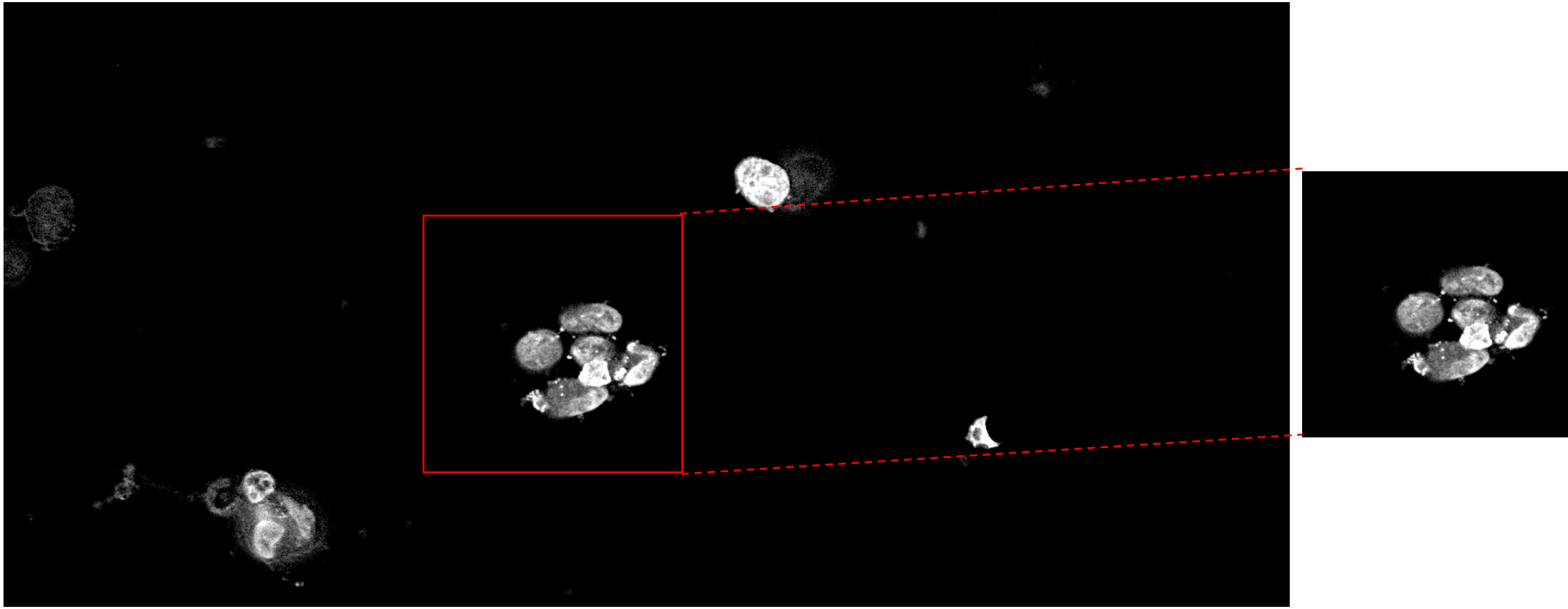

Figure 3-E
WT-Merge

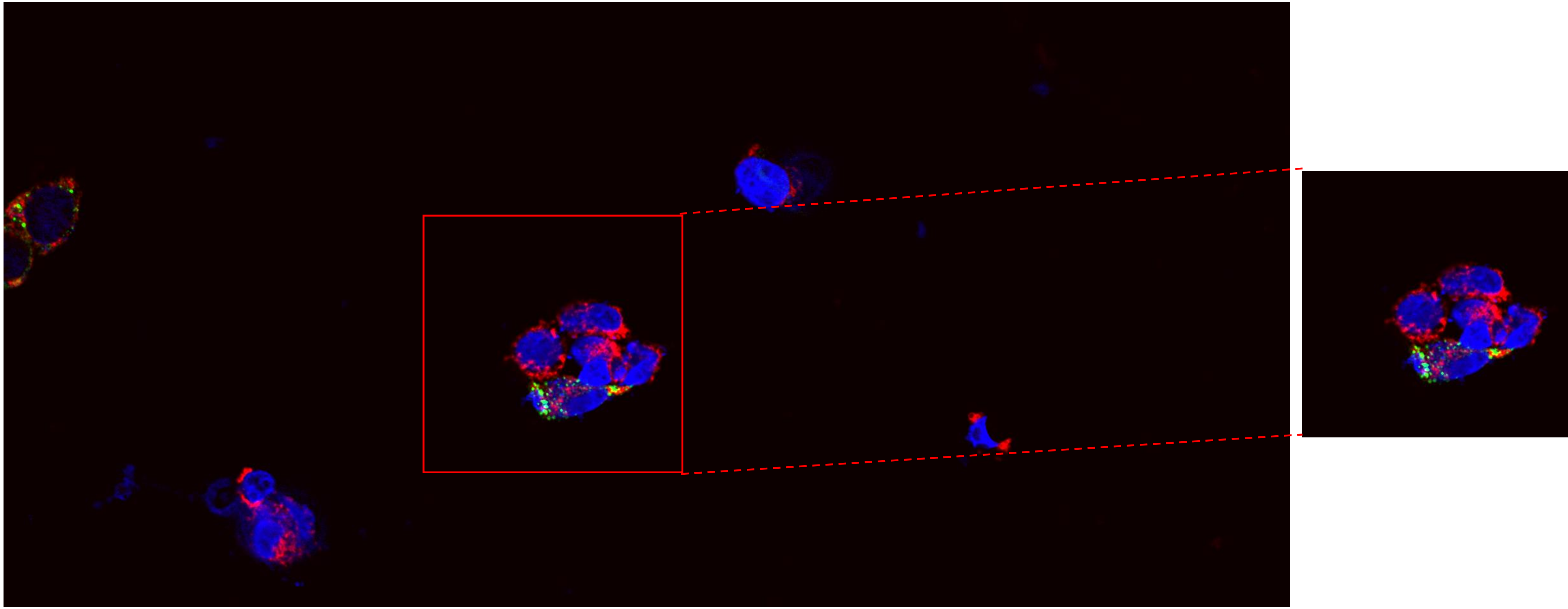

Figure 3-E
MT-GFP

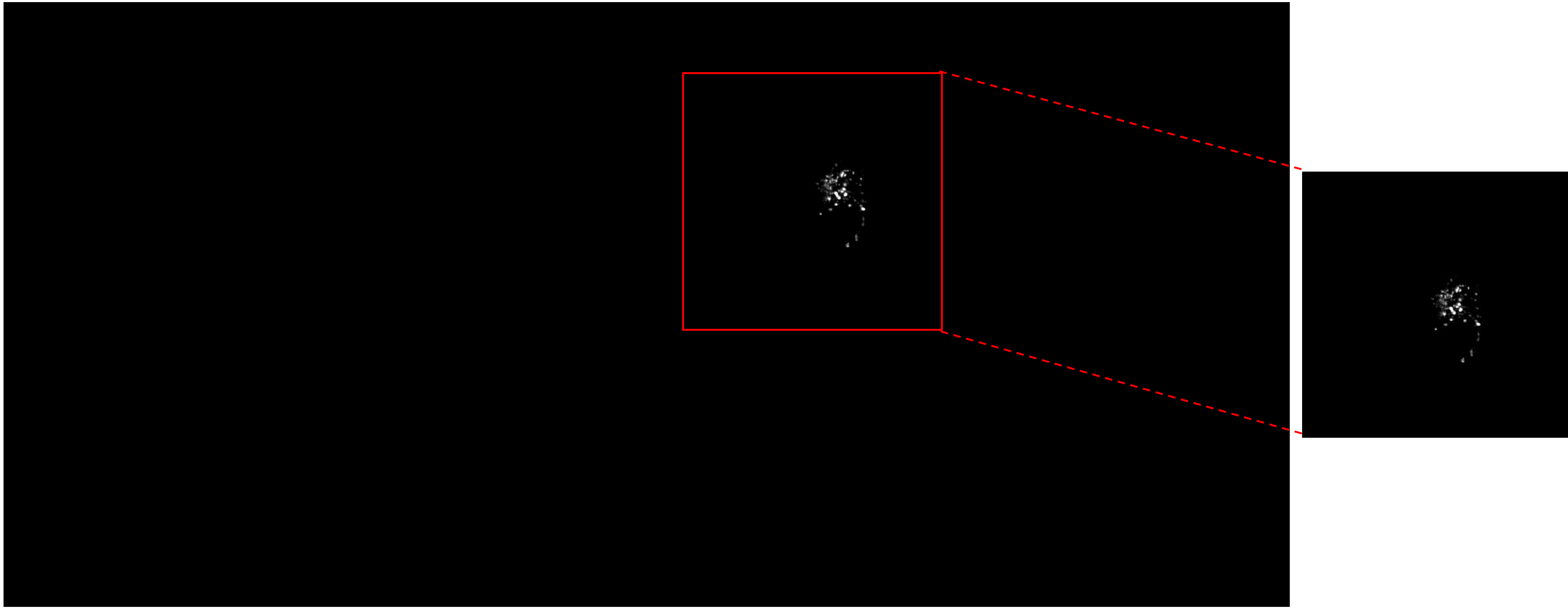

Figure 3-E
MT-TOMM20

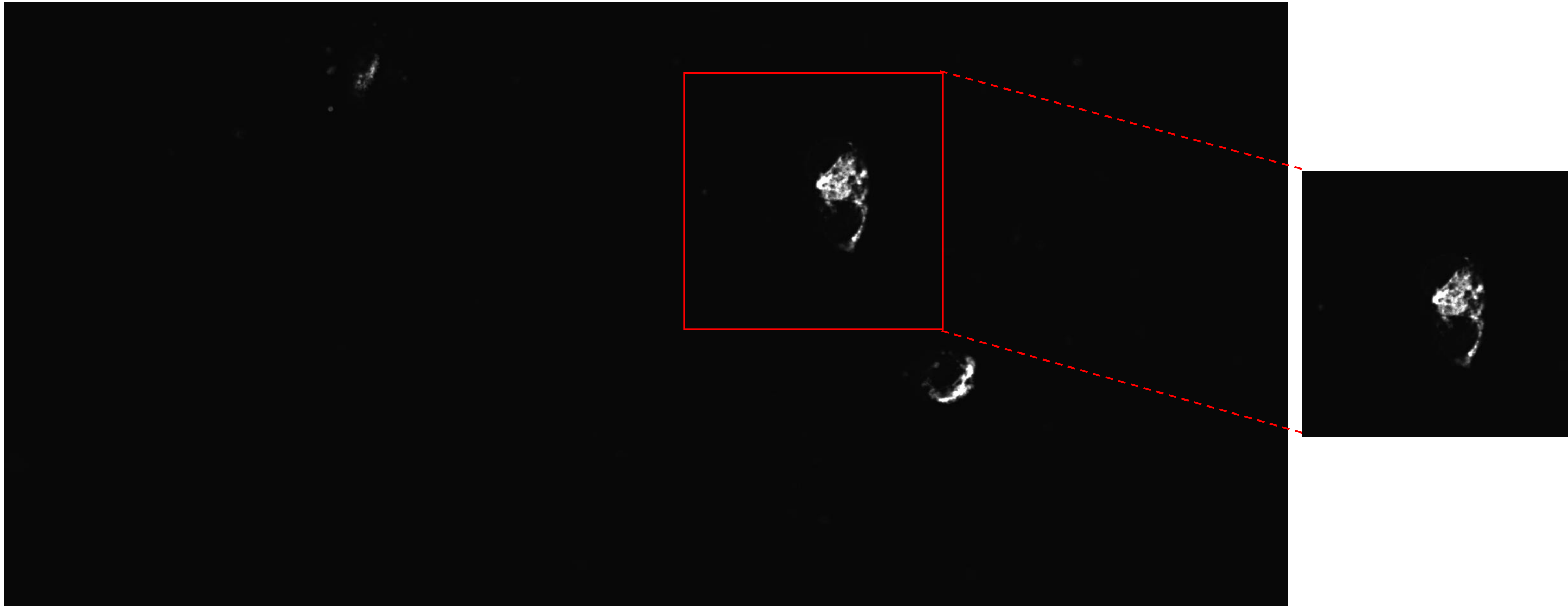

Figure 3-E
MT-DAPI

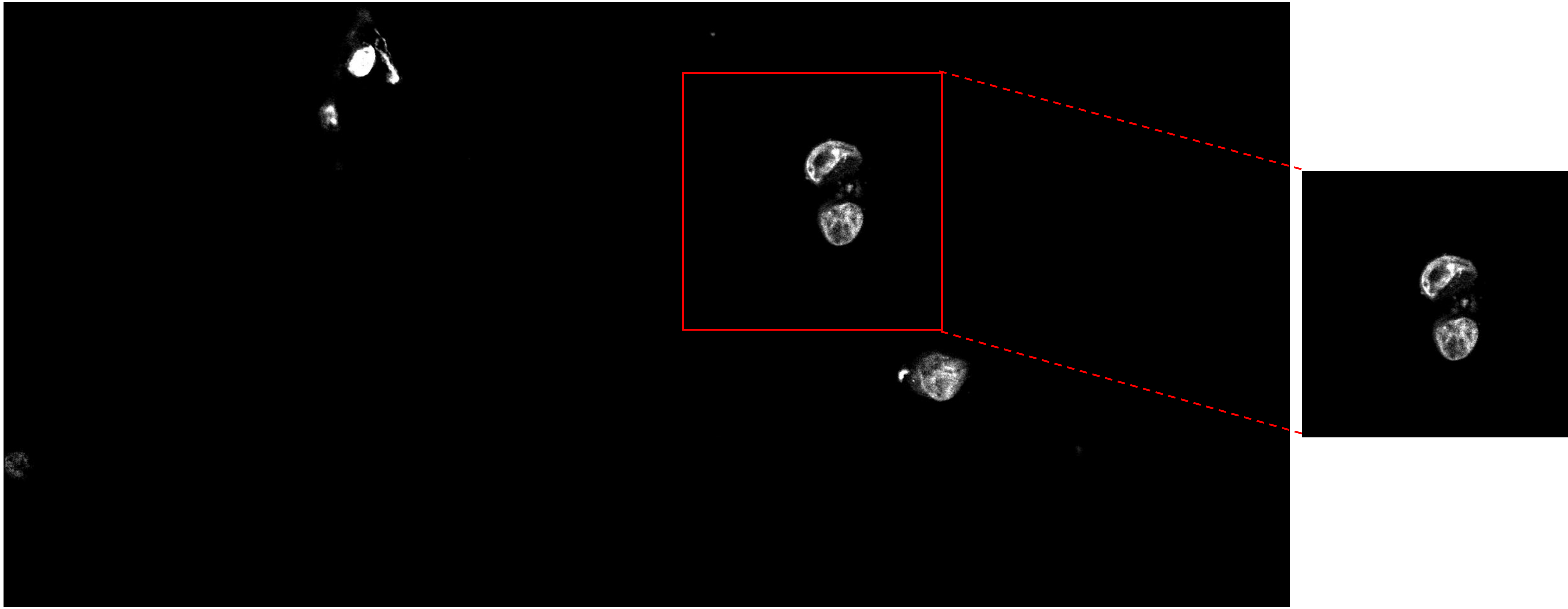

Figure 3-D
MT-Merge

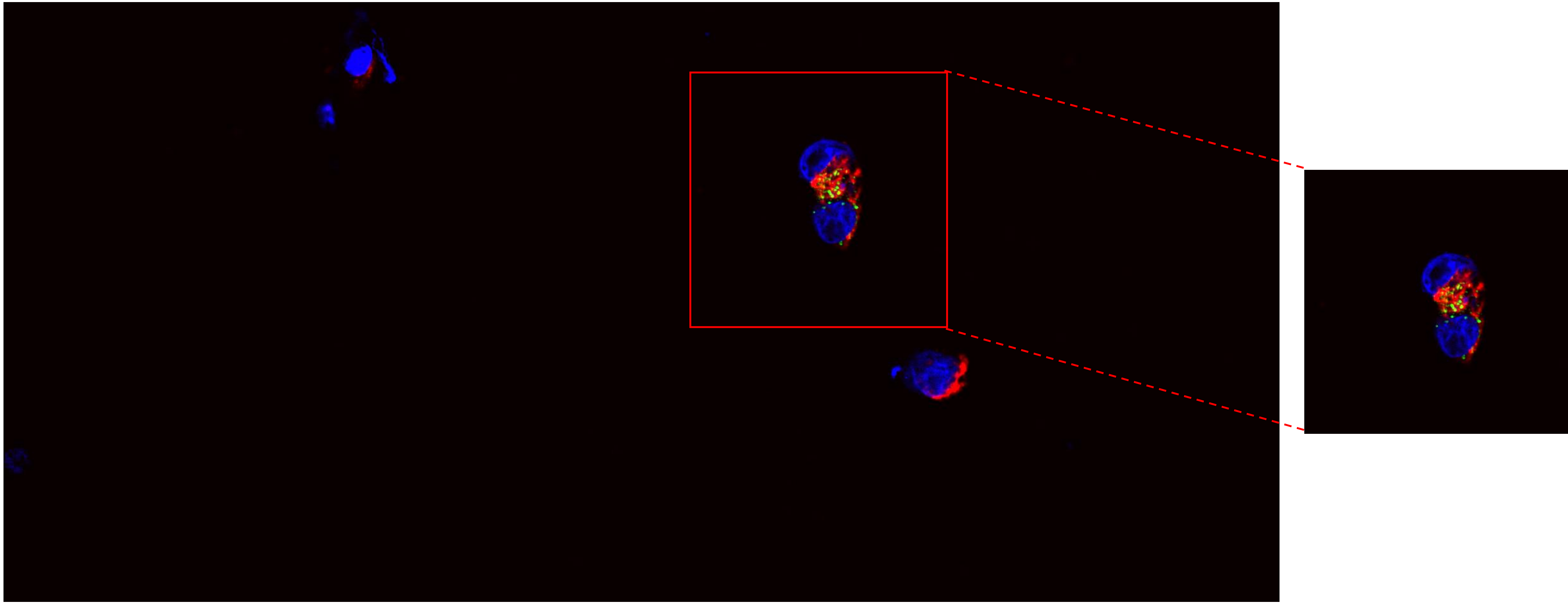

Supplement: Supplementary file 5 — Supplementary Information 5. [file 41598_2024_64943_MOESM5_ESM.pdf]
