# Supplementary figures and images for: M133S mutation possibly involve in the ER stress and mitophagy pathway in maintenance hemodialysis patients with occult hepatitis B infection
Source: Sci Rep. 2024 Jun 17;14:13981. doi: 10.1038/s41598-024-64943-3 (PMC11183135; doi:10.1038/s41598-024-64943-3)

Figure 4-F

PDI 55KD

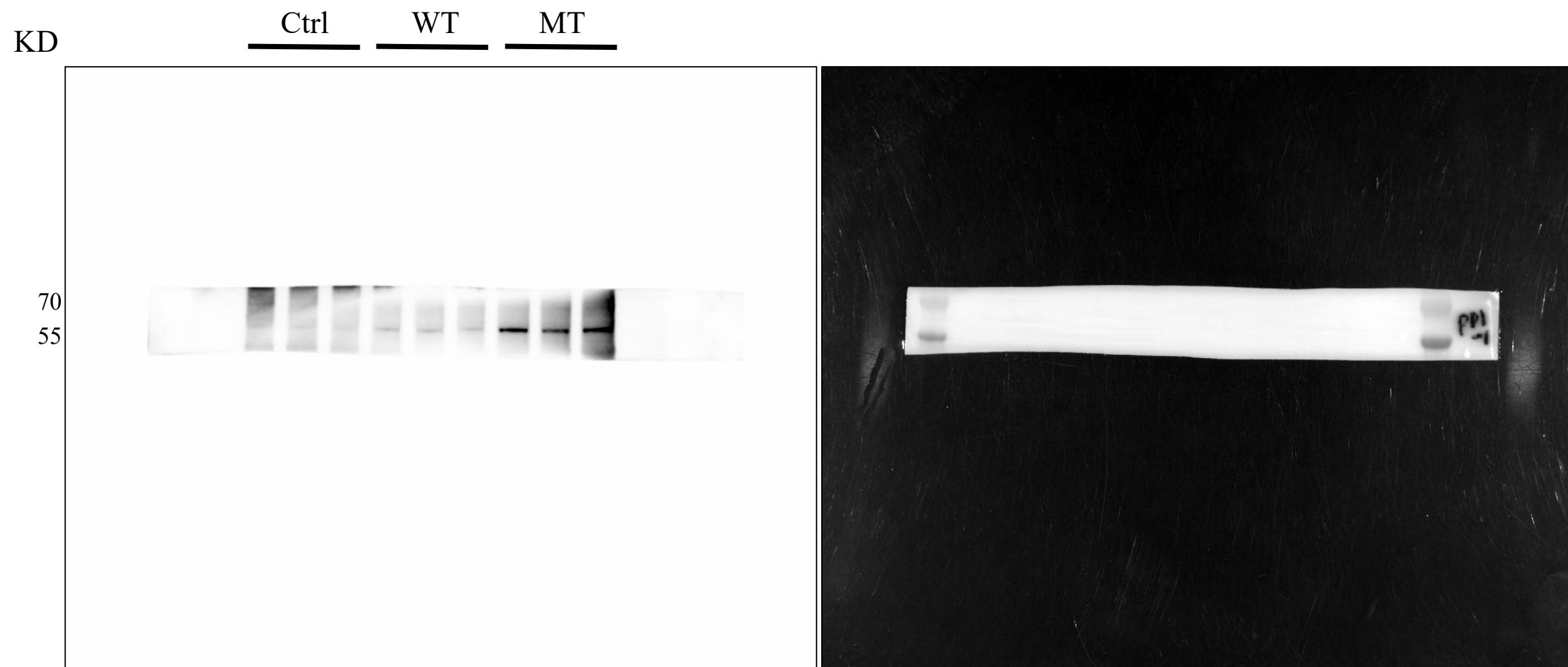

Figure 4F  
LC3 16-18KD

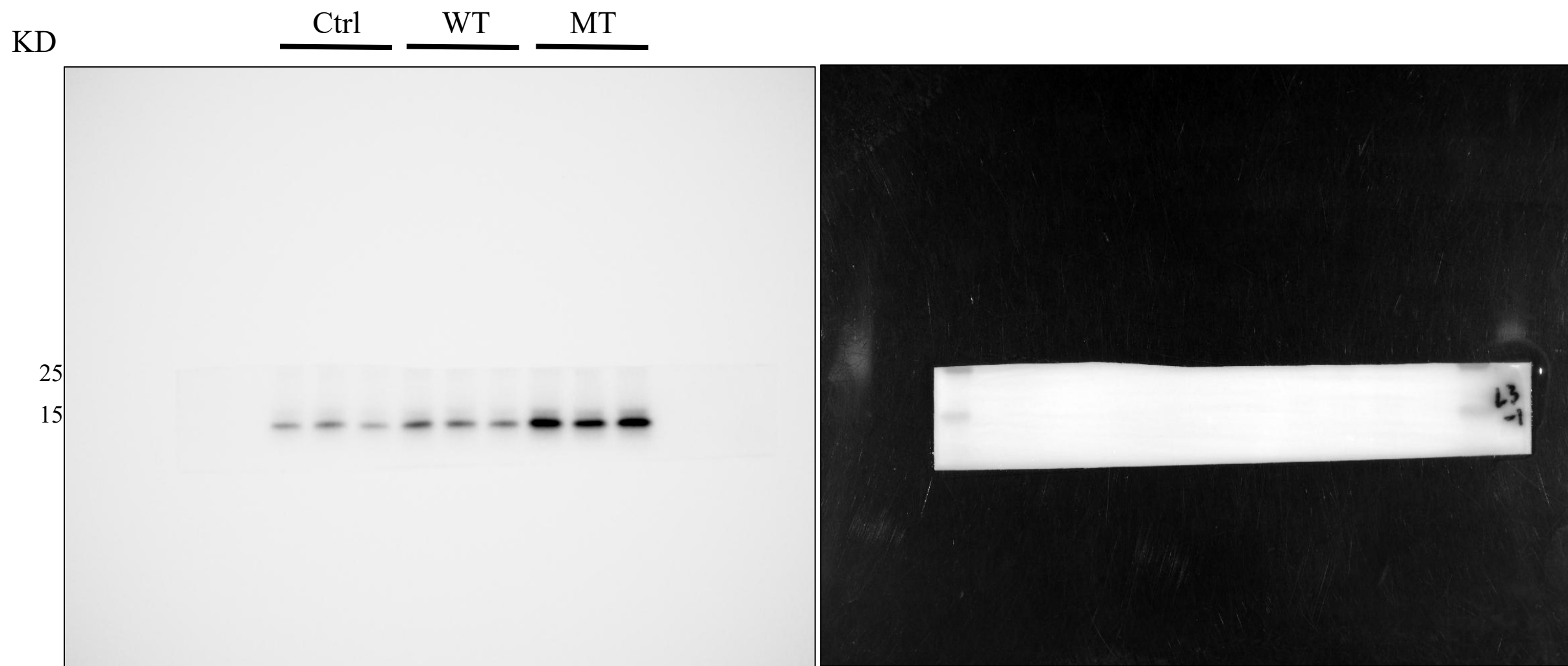

Figure 4F

LAMP2 100-130KD

KD

Ctrl    WT    MT

110

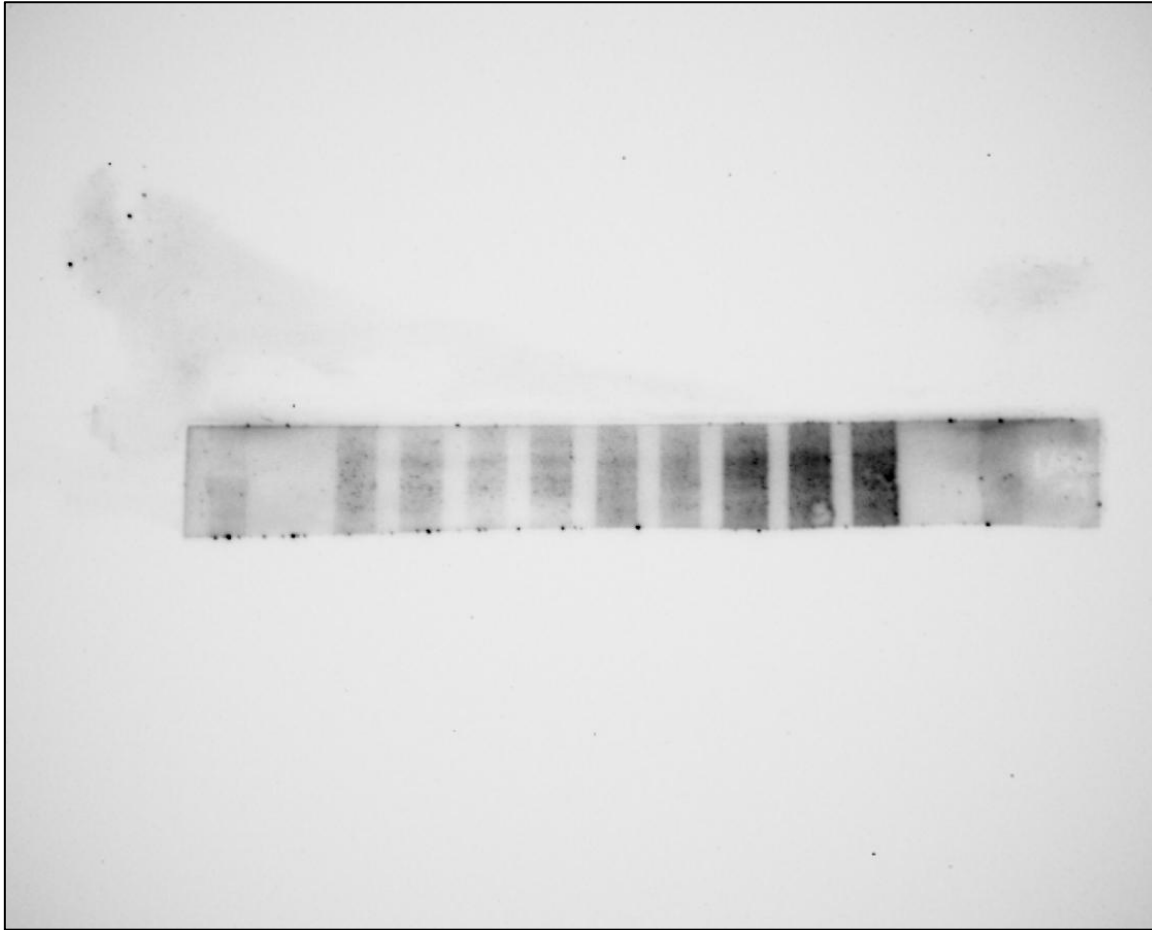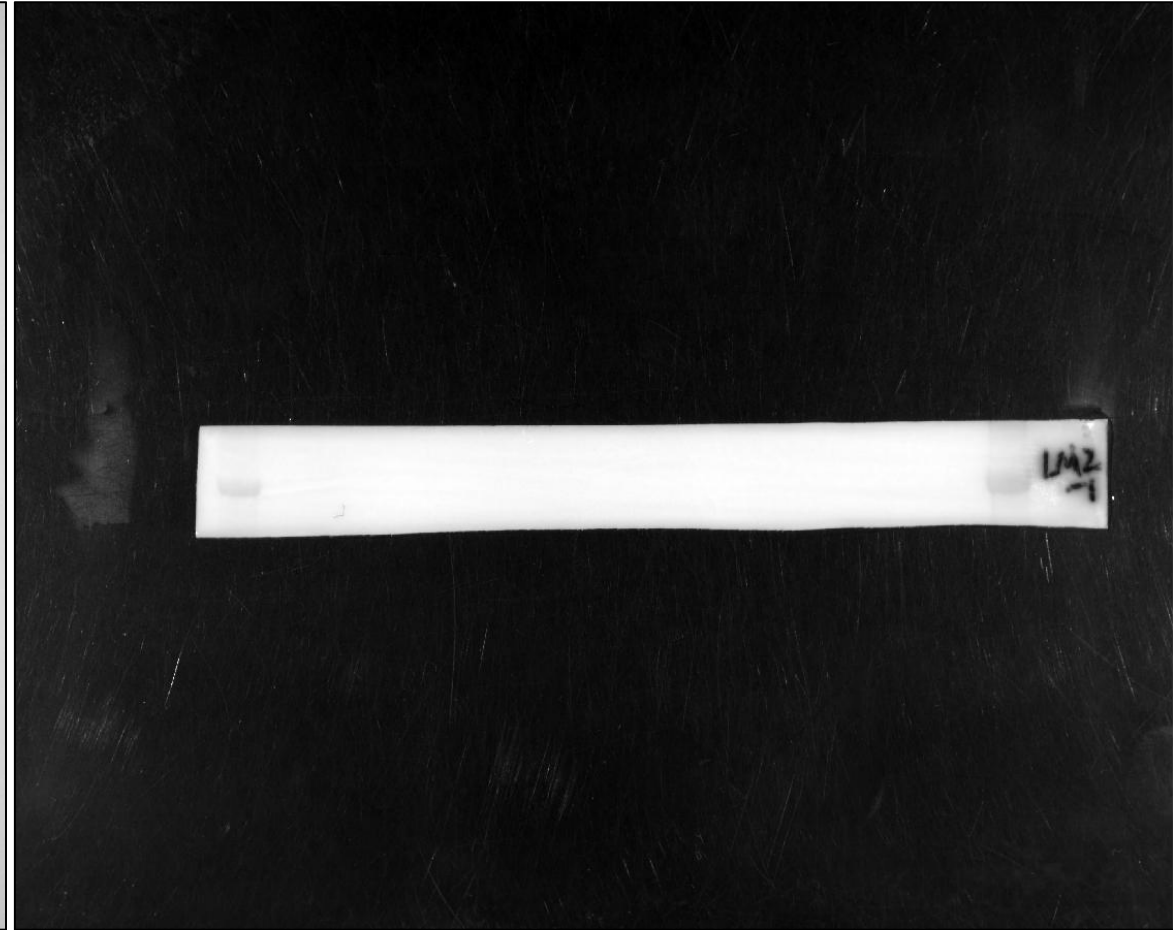

Figure 4F

TOMM20 16KD

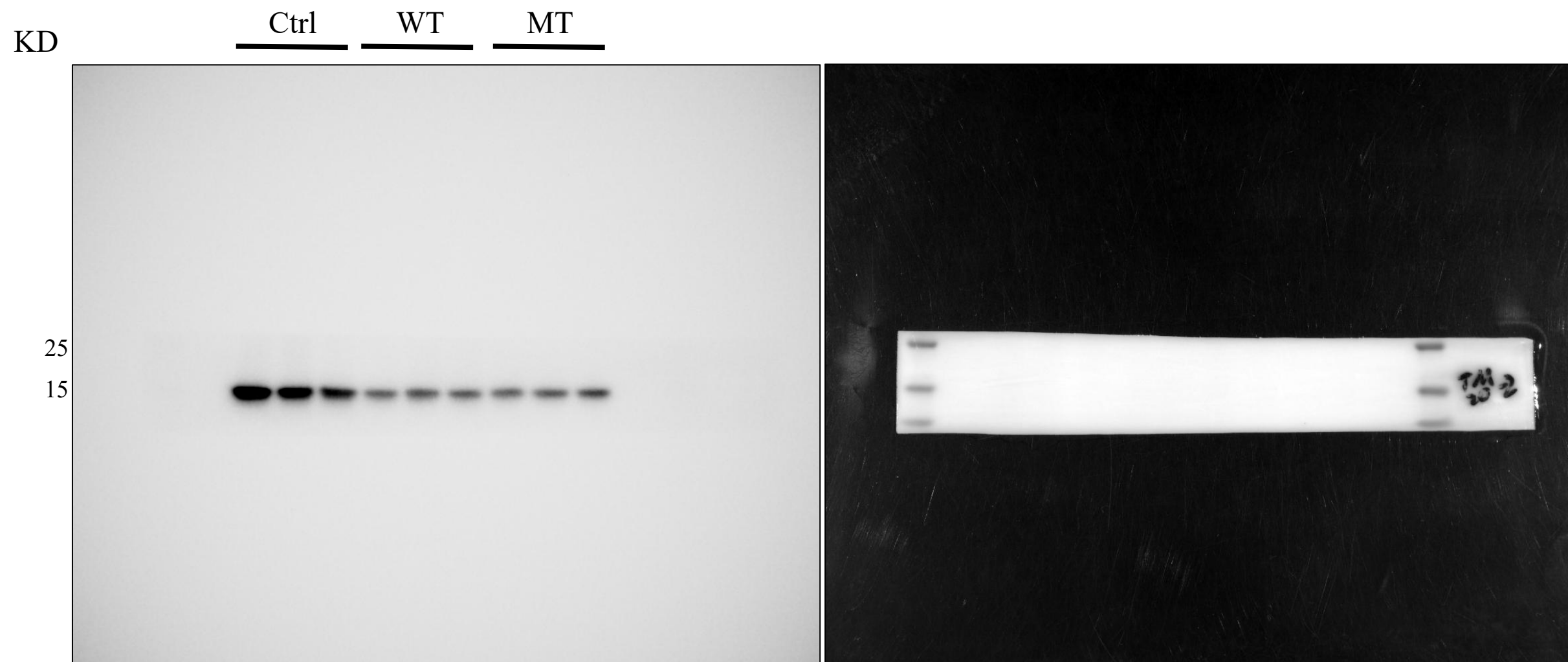

Figure 4F  
 $\beta$ -actin 42KD

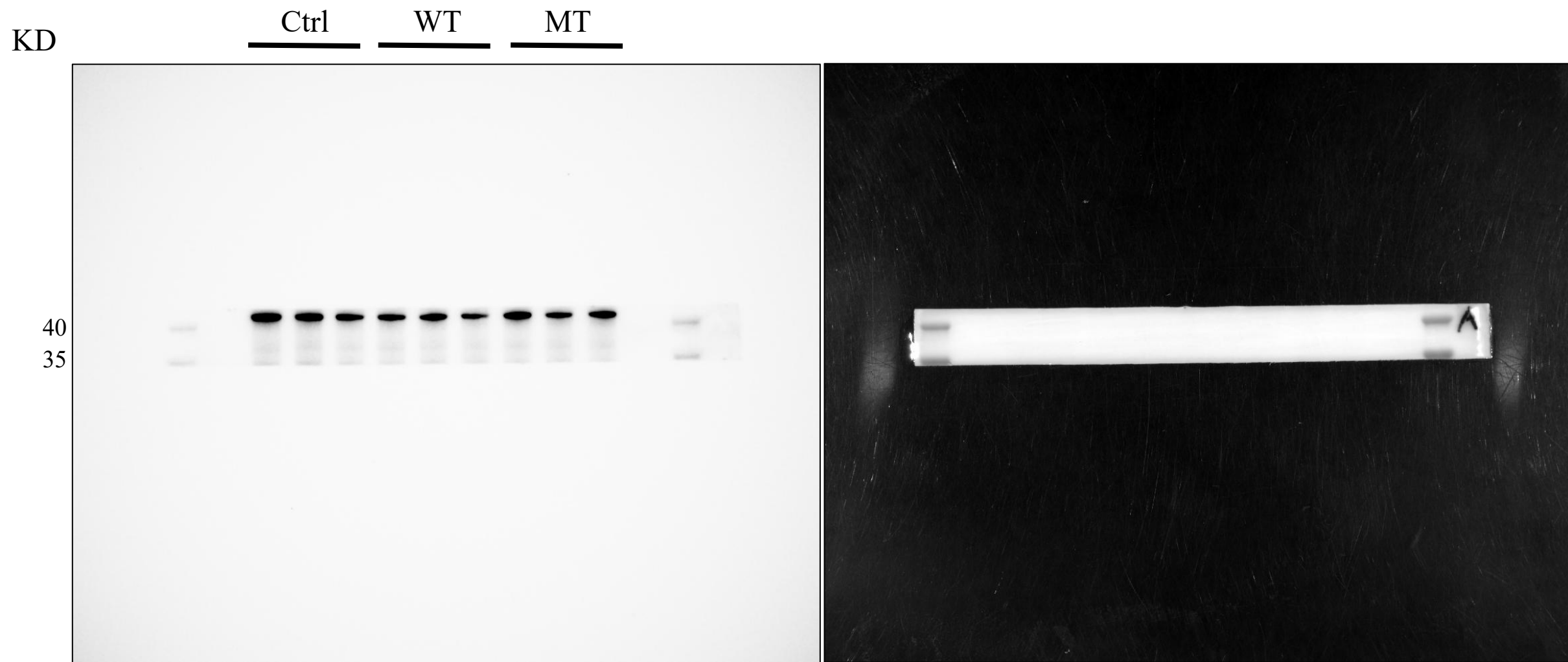

Supplement: Supplementary file 6 — Supplementary Information 6. [file 41598_2024_64943_MOESM6_ESM.pdf]
